# Self-perceived preparedness and training needs of healthcare personnel on humanitarian mission: a pre- and post-deployment survey

**DOI:** 10.1186/s13017-022-00417-z

**Published:** 2022-03-05

**Authors:** Frederike J. C. Haverkamp, Tristan A. J. van Leest, Måns Muhrbeck, Rigo Hoencamp, Andreas Wladis, Edward C. T. H. Tan

**Affiliations:** 1 Department of Surgery, Radboudumc, Internal Postal Code 618, P.O. Box 9101, 6500 HB Nijmegen, The Netherlands; 2 Department of Surgery, Radboudumc, Geert Grooteplein Zuid 10, 6525 GA Nijmegen, The Netherlands; 3 Department of Anaesthesiology, Radboudumc, Geert Grooteplein Zuid 10, 6525 GA Nijmegen, The Netherlands; 4 Department of Surgery in Norrköping, Department of Biomedical and Clinical Sciences and Center for Disaster Medicine and Traumatology, Linköping University, 603 79 Norrköping, Sweden; 5 Defence Healthcare Organization, Ministry of Defence, Herculeslaan 1, 3584 AB Utrecht, The Netherlands; 6 Department of Surgery, Alrijne Medical Center Leiderdorp, Simon Smitweg 1, 2353 GA Leiderdorp, The Netherlands; 7 Department of Surgery, Leiden University Medical Center, Albinusdreef 2, 2333 ZA Leiden, The Netherlands; 8 Department of Surgery, Erasmus Medical Center, Doctor Molewaterplein 40, 3015 GD Rotterdam, The Netherlands; 9 Department of Biomedical and Clinical Sciences and Center for Disaster Medicine and Traumatology, Linköping University, 583 30 Linköping, Sweden

**Keywords:** Deployment; humanitarian aid; mission; training; preparedness; armed conflict

## Abstract

**Background:**

Humanitarian healthcare workers are indispensable for treating weapon-wounded patients in armed conflict, and the international humanitarian community should ensure adequate preparedness for this task. This study aims to assess deployed humanitarian healthcare workers’ self-perceived preparedness, training requirements and mental support needs.

**Methods:**

Medical professionals deployed with the International Committee of the Red Cross (ICRC) between October 2018 and June 2020 were invited to participate in this longitudinal questionnaire. Two separate questionnaires were conducted pre- and post-deployment to assess respondents’ self-perceived preparedness, preparation efforts, deployment experiences and deployment influence on personal and professional development.

**Results:**

Response rates for the pre- and post-deployment questionnaires were 52.5% (114/217) and 26.7% (58/217), respectively. Eighty-five respondents (85/114; 74.6%) reported feeling sufficiently prepared to treat adult trauma patients, reflected by predeployment ratings of 3 or higher on a scale from 1 (low) to 5 (high). Significantly lower ratings were found among nurses compared to physicians. Work experience in a high-volume trauma centre before deployment was associated with a greater feeling of preparedness (mean rank 46.98 vs. 36.89; *p* = 0.045). Topics most frequently requested to be included in future training were neurosurgery, maxillofacial surgery, reconstructive surgery, ultrasound, tropical diseases, triage, burns and newborn noncommunicable disease management. Moreover, 51.7% (30/58) of the respondents regarded the availability of a mental health professional during deployment as helpful to deal with stress.

**Conclusion:**

Overall, deployed ICRC medical personnel felt sufficiently prepared for their missions, although nurses reported lower preparedness levels than physicians. Recommendations were made concerning topics to be covered in future training and additional preparation strategies to gain relevant clinical experience. Future preparatory efforts should focus on all medical professions, and their training needs should be continuously monitored to ensure the alignment of preparation strategies with preparation needs.

**Supplementary Information:**

The online version contains supplementary material available at 10.1186/s13017-022-00417-z.

## Background

The devastating consequences of armed conflicts are markedly reflected by their many civilian casualties who simultaneously are deprived of access to adequate healthcare. A breakdown in the existing healthcare systems caused by conflict raises a pressing need for healthcare delivery by foreign medical teams [1–3]. Over the years, the international community has increasingly stressed the importance of professionalisation and appropriate preparation for deployed emergency medical teams. Unprepared humanitarian efforts may even harm the health system and patient population [4].

The World Health Organization has defined minimum standards for clinical care delivered by emergency medical teams [5, 6]. Suitable training methods are essential to living up to these minimum standards because basic education and residency programmes insufficiently prepare teams for the caseload expected during deployment [7–9]. The types of procedures performed in civilian daily practice and the work conditions substantially contrast with those during deployment to conflict zones.

Predeployment training is essential to fill the gap between skills acquired during primary medical training and skills required during deployment to a conflict area. Many training opportunities currently exist, but geographical, logistical and financial limitations can make it challenging for humanitarian organisations to implement mandatory standardised predeployment training [10–12]. Moreover, the training needs vary widely between individuals based on their previous education and experience. Job titles can be similar, whereas actual competencies are not, due to international differences in the postgraduate educational structure. For example, general surgeons perform fracture surgery in some countries, while in other countries, trauma or orthopaedic surgeons perform this surgery, resulting in different experience levels among these specialities. Therefore, resources should be allocated carefully to select training methods that meet the current needs.

Literature on self-perceived preparedness and training requirements for humanitarian healthcare workers deployed to conflict areas is scarce. However, this information is vital to facilitate deployment preparation efforts for humanitarian organisations, such as the International Committee of the Red Cross (ICRC), which is one of the largest humanitarian aid organisations that specifically operates to alleviate the suffering of victims of armed conflict and provides healthcare for the weapon-wounded. Worldwide, 599 hospitals were supported by the ICRC in 2020 [13].

To better fulfil the training needs of deployed medical teams, a questionnaire was conducted with 153 medical professionals working for the ICRC in 2017 [14]. This first assessment demonstrated the need for additional training in paediatric care, fracture surgery and burn treatment and indicated a lower feeling of preparedness among nurses compared to physicians. The ICRC has since implemented a mandatory onboarding mission prior to the first deployment. An onboarding mission is intended to prepare nurses and physicians for the work environment and injury types they typically encounter while on the mission. During an onboarding mission, nurses and physicians, in addition to the regular staff, work under ICRC supervision.

Persisting training needs should be reassessed regularly to expose trends and developments in deployment preparation strategies, such as the effectiveness of onboarding mission implementation on the preparedness level. Therefore, we conducted a follow-up questionnaire with customised pre- and post-deployment measurements, in contrast to the previous cross-sectional survey. The primary aim was to assess ICRC medical professionals’ self-perceived preparedness and training requirements regarding trauma and emergency care delivered during deployment to conflict zones. The secondary aims were to identify factors associated with a higher feeling of preparedness and assess the need for mental support during and after deployment. Humanitarian organisations or individual healthcare workers can use study findings to optimise their preparation strategies prior to deployment.

## Methods

This survey study consists of two time measurements (pre- and post-deployment) using a digital questionnaire. All enlisted ICRC medical professionals deployed between 1 October 2018 and 30 June 2020 were invited to participate. Potential participants included nurses, physiotherapists and physicians from any speciality. Project information with a personal invitation link was sent by email several weeks before and after deployment. Reminders were automatically sent two weeks after the initial survey invitation. Participants could complete the questionnaires until 31 July 2020. Participation in the survey was voluntary, with respondents consenting to participate by submitting their responses. The pre- and post-deployment responses were paired and rendered anonymous. All data were stored on encrypted servers of the Radboudumc, which are only accessible by research team members.

The survey design was similar to a previous survey among ICRC medical professionals [14], which was based on a review of existing questionnaires and expert opinions [15–20]. The predeployment survey contained questions regarding respondents’ background characteristics, self-perceived preparedness and predeployment preparations (see Additional file 1). The post-deployment survey contains questions about self-perceived preparedness regarding the encountered injuries, deployment experiences, (human) resources during deployment, and deployment influence on personal and professional development (see Additional file 1). When asking about work experience in a high-volume trauma centre, this was defined as a hospital with high rates of severe trauma injuries with pathologies expected to mimic those during deployment.

Descriptive statistics are displayed as numbers with percentages or medians with interquartile ranges. Due to the quantity of the data, part of the data are displayed in the additional material (Additional file 2). The main results are presented in this manuscript. The primary outcome measure was the rating of self-perceived preparedness to treat paediatric and adult trauma patients. Comparative analyses were performed to compare the rating of self-assessed preparedness for multiple independent variables, including profession (physician or nurse, with subanalyses among surgeons, anaesthesiologists and nurses), previous deployments, years of clinical experience, predeployment training (clinical experience and onboarding mission) and country of basic medical education (low-, middle- or high-income countries). Additional subanalyses were performed with independent variables to assess the potential relationship with the satisfaction rating of the available equipment, need for psychological support, and deployment influence on personal and professional development. Respondents with a matched pre- and post-deployment response were compared to respondents with a one-time response based on their background characteristics and self-perceived predeployment preparedness, to assess whether dropouts significantly differ from respondents included in follow-up.

### Statistical methods

Chi-square testing was used to compare categorical variables between groups unless the sample size was less than 10 observations per group, for which a Fisher’s exact test was used. The Mann–Whitney U and Kruskal Wallis tests were used to compare continuous variables between two or more than two groups, respectively. Mean ranks are displayed for variables that were compared using these nonparametric tests. The mean rank is calculated by ranking all absolute values included in the analysis from the smallest (1) to the largest value (*n* = the total number of values included). Then, for each group included in the comparison, the mean is calculated of these ranks and it is tested whether these mean ranks differ between groups. The Wilcoxon signed-rank test was used to compare the paired pre- and post-deployment ratings of self-perceived preparedness.

Missing data were regarded as missing at random, and missing variables were excluded per analysis. An α level of 0.05 or lower was regarded as significant. All analyses were performed using IBM SPSS Statistics (version 25, IBM Corporation, Armonk, New York).

## Results

Predeployment questionnaires were completed by 40.1% (87/217) of the invitees and were partially filled out by an additional 12.4% (27/217) of the invited study population. Post-deployment questionnaires were completely and partially filled out by 25.3% (55/217) and 1.4% (3/217) of invitees, respectively. There were 42 matched pre- and post-deployment responses (19.4%; 42/217).

Respondents who submitted only one response (dropouts) were previously deployed with Médecins Sans Frontières for a longer period than respondents with a matched pre- and post-deployment response (mean rank 25.3 vs. 15.7; *p* = 0.010). No other differences were found between these groups in the background characteristics or self-perceived predeployment preparedness.

### Background characteristics

Table 1 lists the background characteristics of the respondents. Over 60% (69/114) of respondents had been deployed with the ICRC at least two times prior. The number of weeks of deployment with the ICRC did not differ significantly between physicians and nurses (mean rank 42.9 vs. 40.0; *p* = 0.605). In addition, 57% of respondents (65/114) completed their primary education in a high-income country, 36.0% (41/114) in a middle-income country, and 7.0% (8/114) in a low-income country. Most medical professionals completed their education in Europe (51/114; 44.7%) or Africa (27/114; 23.7%).

**Table 1:** Background characteristics

Variable	Value (*n* = 114)
*Sex (N, %)*	
Male	76 (66.7%)
Female	36 (31.6%)
Missing	2 (1.8%)
*Age (years)*	
Median, IQR	45.5 (18.8)
Missing	2 (1.8%)
*Continent of primary medical education*	
Africa	27 (23.7%)
Asia	15 (13.2%)
Europe	51 (44.7%)
Oceania	4 (3.5%)
North America	7 (6.1%)
South America	10 (8.8%)
Missing	0 (0.0%)
*Country of primary medical education*^*a*^	
Low income	8 (7.0%)
Middle income	41 (36.0%)
High income	65 (57.0%)
Missing	0 (0.0%)
Profession (N, %)	*n* = 130^b^
Anaesthesiologist	27 (20.8%)
Surgeon	47 (36.2%)
Emergency Room physician	5 (3.8%)
Nurse	48 (36.9%)
Other	1 (0.8%)
Missing	2 (1.5%)
*Years since registration as a nurse*	
Median, IQR	13 (9.2)
Missing	8 (20.0%)
*Years since board registration as a medical specialist*	
Median, IQR	16 (18.0)
Missing	3 (4.2%)
*Years of clinical experience*	
Median, IQR	15 (13.8)
Missing	6 (5.3%)
*Years between most recent pre-deployment course and next deployment*	
Median, IQR	1.0 (1.0)
Missing	40 (35.1%)
*Years between onboarding mission and next deployment*	
Median, IQR	1.0 (4.0)
Missing	56 (49.1%)
*Number of previous deployments with the ICRC (N, %)*	
0	24 (21.1%)
1	15 (13.2%)
≥ 2	69 (60.5%)
Missing	6 (5.3%)
*Number of previous deployments with MSF (N, %)*	
0	68 (59.6%)
1	7 (6.1%)
≥ 2	32 (28.1%)
Missing	7 (6.1%)
*Number of previous deployments with armed forces (N, %)*	
0	88 (77.2%)
1	7 (6.1%)
≥ 2	13 (11.4%)
Missing	6 (5.3%)
*Worked in hospital in home country during the last year prior to deployment (N, %)*	
Yes	66 (57.9%)
No	21 (18.4%)
Missing	27 (23.7%)
*Experience with paediatric trauma during the last two years before deployment (N, %)*	
No	8 (7.0%)
Sporadically/monthly	63 (55.3%)
Frequently/weekly	37 (32.5%)
Missing	6 (5.3%)

^a^According to the World Bank classification [21]

^b^n = 130 because profession is the only background characteristic that was also filled out in the postdeployment questionnaire

Regarding the respondents’ motivation to work with the ICRC, the three most frequently reported reasons were ‘wanting to help people afflicted by war and disaster’ (91/114; 79.8%), ‘wanting to help people less fortunate in healthcare options’ (81/114; 71.1%) and ‘the opportunity to work together with colleagues from different nationalities’ (66/114; 57.9%) [21].

### Self-perceived preparedness

Table 2 presents the median pre- and post-deployment ratings of self-perceived preparedness to treat adult and paediatric trauma patients. Among predeployment ratings, 85 respondents (85/114; 74.6%) reported feeling sufficiently prepared (rating of 3 or higher on the 1–5 scale) to treat adult trauma patients, and 81 respondents (81/114; 71.1%) reported sufficient self-perceived preparedness to treat paediatric patients. Nurses reported a lower feeling of preparedness than physicians for managing paediatric and adult trauma patients (Table 2). The median rating of self-perceived preparedness to treat paediatric patients was slightly higher (median 4.5) post-deployment as rated by physicians and slightly lower (median 3.0) as rated by nurses, compared to predeployment ratings (physicians’ median 4.0; nurses’ median 4.0). These differences were not statistically significant (comparison among physicians at *p* = 0.739 and nurses at *p* = 0.527).

**Table 2:** Pre- and post-deployment rating of self-perceived preparedness

Self-perceived preparedness	Physicians	Nurses	*p* value for comparison between physicians and nurses
*Pre-deployment*			
Paediatric trauma			
Median (IQR)	4.0 (1.0)	4.0 (1.0)	0.024
Mean rank	47.78	35.13	
Missing (N, %)	11 (15.3%)	14 (35.0%)	
Adult trauma			
Median (IQR)	5.0 (1.0)	4.0 (1.0)	0.023
Mean rank	47.43	35.94	
Missing (N, %)	11 (15.3%)	14 (35.0%)	
*Post-deployment*			
Paediatric trauma			
Median (IQR)	4.5 (1.0)	3.0 (2.0)	0.004
Mean rank	31.47	19.73	
Missing (N, %)	2 (2.7%)	3 (7.5%)	
Adult trauma			
Median (IQR)	5.0 (0.0)	4.0 (2.0)	0.002
Mean rank	32.16	20.77	
Missing (N, %)	3 (4.2%)	1 (2.5%)	

Rating scale 1–5 (1 very unprepared—5 more than sufficiently prepared)

IQR interquartile range

Respondents who gained additional work experience in a high-volume trauma centre prior to deployment (*n* = 33) felt better prepared than medical professionals who did not, as rated predeployment for paediatric trauma (mean rank 46.98 vs mean rank 36.89; *p* = 0.045). Subanalyses did not reveal any statistically significant correlation between the feeling of preparedness and the previous deployment, onboarding mission, country of primary medical education or years of clinical experience.

After deployment, respondents were asked to rate their training, knowledge and skills regarding the injuries they treated during deployment. Physicians rated this significantly higher (median 5.0; IQR 1.0; mean rank 32.03) than nurses (median 3.0; IQR 3.0; mean rank 20.43; *p* = 0.003). The medical training, knowledge and skills of direct colleagues during deployment were also rated. Again, physicians rated this higher (median 5.0; IQR 1.0; mean rank 30.45) than nurses did (median 4.0; IQR 2.0; mean rank 22.50; *p* = 0.041). When balancing the feeling of preparedness to treat paediatric patients against preparedness to treat adult patients, most respondents reported feeling equally confident (26/58; 44.8%).

### Predeployment preparations

Respondents attended various courses as part of their predeployment preparations, and the most frequently attended courses for physicians were the Advanced Trauma Life Support (ATLS) course (surgeons 29/47; 61.7% and anaesthesiologists 9/27; 33.3%) and ICRC War Surgery Seminar (surgeons 23/47; 48.9% and anaesthesiologists 9/27; 33.3%). Nurses most frequently attended the Advanced Cardiovascular Life Support course (9/40; 22.5%). Two surgeons and two nurses did not attend any courses prior to deployment. Ten surgeons (10/47; 21.3%), five anaesthesiologists (5/27; 18.5%) and five nurses (5/48; 10.4%) went on an onboarding mission.

The most important activity as part of predeployment preparations was considered previous emergency care experience (56/114; 49.1%). The second-most important were previous deployments (47/114; 41.2%), and third was the basic education programme for their current profession (34/114; 29.8%). As part of the deployment preparation, 33 respondents (33/114; 28.9%) gained additional work experience in a high-volume trauma centre, and 38 respondents (38/114; 33.3%) were able to practice with the equipment they would have at their disposal during deployment. Overall, respondents rated predeployment information about various aspects of their mission as sufficient, with median ratings of 4.0 and 5.0 on a 1–5 scale (1 indicating not at all adequately informed and 5 indicating more than sufficiently informed).

The five topics most frequently proposed to be included in future predeployment training are listed per speciality in Table 3. Subanalyses revealed various factors correlated with the number of topics requested for additional training. Surgeons (mean rank 66.19) requested more topics for additional training than anaesthesiologists (mean rank 39.74; *p* = 0.002). Respondents who had been on previous deployments (mean rank 52.51) proposed fewer topics for additional training than those who had not (mean rank 84.40; *p* = 0.023), whereas respondents who went on an onboarding mission (mean rank 67.52) requested more topics for additional training than those who did not (mean rank 53.92; *p* = 0.050).

**Table 3:** Topics requested for additional training and underlying reasons, stratified by profession

	Topic	Requested (*N*, %)^a^
*Surgeons (N = 42)*		
1	Neurosurgery	21 (50.0%)
2	Maxillofacial surgery	20 (47.6%)
3	Plastic (reconstructive) surgery	20 (47.6%)
4	Sonography/ultrasound skills	17 (40.5%)
5	Vascular surgery	16 (38.1%)
	No need/none^b^	1 (2.4%)
*Anesthesiologists (N = 25)*		
1	Sonography/ultrasound skills	10 (40.0%)
2	Tropical diseases	6 (24.0%)
3	Triage skills	5 (20.0%)
4	Antibiotic management	4 (16.0%)
5	Intensive care	4 (16.0%)
	No need/none^b^	1 (4.0%)
*Nurses (N = 40)*		
1	Triage and mass casualty management	14 (35.0%)
2	Burns patients	11 (27.5%)
3	New-born non-communicable disease management	11 (27.5%)
4	Tropical disease management	9 (22.5%)
5	Paediatric patients	8 (20.0%)
	Intensive care	8 (20.0%)
	Wound care	8 (20.0%)
	No need/none^b^	1 (2.5%)

^a^Missing: surgeons 3 (7.1%); anaesthesiologists 7 (28.0%); nurses 18 (45.0%)

^b^Missing ‘no need for training’: surgeons 2 (4.8%); anaesthesiologists 6 (24.0%); nurses 17 (42.5%)

Humanitarian military cooperation in predeployment training was regarded as advantageous by 39 (39/114; 34.2%) respondents, whereas 9 respondents (9/114; 7.9%) reported they were against such cooperation and 39 respondents (39/114; 34.2%) reported they did not have any opinion about this statement. Sharing knowledge and experiences was mentioned multiple times as a reason to vote in favour of such cooperation. The lack of impartiality and neutrality were the main reasons respondents voted against humanitarian military cooperation.

### Deployment experiences

More than one-third (22/58; 37.9%) reported being on call 24 h per day, and almost half (27/58; 46.6%) reported being on call seven days per week. The caseload mostly ranged from 1 to 20 cases per week for surgeons (11/23; 47.8%), anaesthesiologists (3/7; 42.9%) and nurses (9/25; 36.0%). The frequency at which injuries outside the respondent’s field of expertise were encountered varied widely. Surgeons most often reported encountering such injuries a few times a month (7/23; 30.4%), whereas anaesthesiologists reported this to happen less than once a month (4/7; 57.1%). Nurses mostly reported that this happened a few times a week (6/25; 24.0%). Regarding communication during deployment, 23 respondents (23/58; 41.8%) reported that no structured methods of communication were used during the handover of patient information.

### (Human) resources during deployment

The equipment medical professionals had at their disposal during deployment was rated on a 1 to 5 scale (1 very dissatisfied to 5 very satisfied; see Additional file 2, Section 1.17). Subanalyses indicated that physicians (mean rank 27.96) were more satisfied than nurses (mean rank 19.65; *p* = 0.028) with the equipment for adult patients in the operation room. Respondents who completed their medical education in a high-income country were less satisfied with the equipment for adults (mean rank 14.90) and paediatric patients (mean rank 14.18) in the operation room than respondents who completed their education in middle-income countries (adults: mean rank 23.11 with *p* = 0.049; paediatric patients: mean rank 23.35 with *p* = 0.030).

Thirty-two respondents (32/58; 55.2%) reported that more experienced colleagues were available for consultation at the deployment site. A referral centre was available for paediatric and adult patients for 24.1% of the respondents (14/58). A substantial proportion of respondents reported that it took more than two hours to reach a referral centre for paediatric patients (14/58; 24.1%) and adult patients (15/58; 25.9%).

### Deployment influence on personal and professional development

Respondents’ ratings of how their deployment affected their professional skills are displayed in Table 4. Overall, deployment influence on respondents’ personal development was rated at a median of 5.0 (IQR 1.3) on a 1 to 5 scale (1 = major negative effect, 3 = neutral and 5 = major positive effect), and the influence on respondents’ private situations was rated at a median of 3.0 (IQR 1.0). A substantial need for peer-to-peer support and debriefing during deployment was reported, whereas respondents experienced a lower need for professional psychological help during their last deployment (Table 4).

**Table 4:** Deployment effect on personal and professional development

	Median (IQR) *N* (%)
*Deployment effect on*	
Trauma management skills^a^	4.0 (IQR 2.0)
Missing	3 (5.2%)
Skills of primary specialism^a^	4.0 (IQR 2.0)
Missing	8 (13.8%)
Personal development^b^	5.0 (IQR 1.3)
Missing	4 (6.9%)
Private situation^b^	3.0 (IQR 1.0)
Missing	5 (8.6%)
*Need for*	
Peer-to-peer contact^c^	4.0 (IQR 2.0)
Missing	4 (6.9%)
Debriefing^c^	4.0 (IQR 1.0)
Missing	3 (5.2%)
Professional psychological help^c^	1.0 (IQR 2.0)
Missing	5 (8.6%)

*IQR* interquartile range

^a^Rating scale 1–5 (1 much deteriorated—3 neutral—5 much improved)

^b^Rating scale 1–5 (1 major negative—3 neutral—5 major positive)

^c^Rating scale 1–5 (1 not at all—5 very much)

Respondents who gained additional work experience in a high-volume trauma centre prior to deployment reported less need for professional help (mean rank 19.82) than respondents who did not (mean rank 29.58; *p* = 0.027). It was also reported how many respondents received peer-to-peer contact (38/58; 65.6%) and psychological help (1/58; 1.7%) and attended a debriefing session (45/58; 77.6%).

Various activities were regarded to help deal with stress during deployment, and the most frequently indicated suggestion was ‘availability of a mental health professional during deployment’ (30/58; 51.7%). The importance of formal debriefing was rated at a median of 4.0 (IQR 2.0) on a scale from 1 to 5 (1 = not important at all and 5 = absolutely essential), and informal debriefing was rated at a median of 5.0 (IQR 2.0).

An open question was posed to respondents about what training they would recommend for colleagues preparing for their first mission in a conflict zone. Several topics and themes were mentioned: psychological preparedness, stress management, multicultural preparedness, communication and teamwork, emergency preparedness, war surgery preparedness, information on mission logistics, attendance of the ATLS course, and security and safety training.

## Discussion

This follow-up survey demonstrated that healthcare workers deployed with the ICRC generally rated their preparedness level to treat trauma patients as sufficient, although nurses reported a significantly lower feeling of preparedness than physicians. Additional work experience in a high-volume trauma centre before deployment was associated with a higher feeling of preparedness to treat paediatric trauma patients and a lower need for professional psychological help. Half of the respondents suggested that the availability of a mental health professional would be helpful to deal with stress during deployment. Topics most frequently requested for inclusion in future training were neurosurgery, maxillofacial surgery, reconstructive surgery, tropical diseases, newborn noncommunicable disease management, burns, triage and ultrasound.

Strikingly, despite the sufficient median rating of preparedness, the proportion of respondents who rated their predeployment preparedness level as sufficient (rating of 3 or higher on a 1–5 scale) decreased to 75% in this survey compared to 93% in the survey in 2017 [14]. However, no substantial differences were observed in the respondents’ background characteristics or previous work experience. The potential influence of some contextual changes between the two study periods is discussed further.

One development was the broad implementation of an onboarding mission within the ICRC in 2017. In addition to the knowledge and skills gained, onboarding missions could be essential in preparing healthcare workers for the challenges they face when working in resource-restrained conflict settings. An observed decrease in self-perceived preparedness after deployment compared to the predeployment rating by nurses and anaesthesiologists supports this notion [22]. Furthermore, respondents who had been on an onboarding mission requested additional training on a greater number of topics than those who did not. These findings suggest that onboarding missions increase the awareness of the potential skill gaps among deployed healthcare workers and potentially stimulate efforts to fill these gaps for forthcoming missions.

Another noted trend was that the distribution of ICRC healthcare workers’ nationalities has substantially changed compared to a previous study among ICRC humanitarian aid workers, in which 21% comprised medical personnel [23]. In 2009, the nationality of 80.1% of the humanitarian aid workers was European and 3.5% was African, whereas, respectively, these percentages are likely to be substantially lower and higher in the study period between 2018 and 2020, based on the continents where ICRC medical personnel completed their primary medical education (44.7% in Europe and 23.7% in Africa). Although healthcare workers from different settings are expected to have different skillsets and gaps, it is complicated to predict whether different nationalities are associated with various preparedness levels. Medical personnel from low- and middle-income countries could be better adapted to work in resource-constrained conflict settings given the broader skillset needed to work in a Level I hospital in a low-income country compared to a high-income country [24]. For instance, penetrating trauma is frequently encountered in armed conflicts and is much more common in low-income countries than high-income countries [25, 26]. Conversely, specialised physicians from high-income countries often have specific in-depth knowledge and access to more specialised preparatory training opportunities. Perhaps cross matching these different medical backgrounds within deployment teams would yield the optimal team composition.

Additionally, this study identified multiple job-specific subjects that humanitarian healthcare workers want included in future training. Compared to our study from 2017, a shift was observed from fundamental topics, such as paediatrics, fracture surgery and burn treatment, to more specialised topics, such as neurosurgery, maxillofacial surgery, plastic surgery, sonography and newborn noncommunicable disease management. A similar pattern was found when comparing caseload aboard the United States Navy hospital ship during humanitarian missions between 2015 and 2018 [27, 28]. This trend could result from a change in caseload or the greater availability of more advanced equipment at ICRC’s healthcare facilities, allowing for more advanced care. In line with this notion, several respondents requested sonography training, which has become available at most ICRC deployment sites during the study period. The lower request for additional training in paediatrics is also reflected by a greater proportion of respondents (71%) who indicated feeling sufficiently prepared to treat paediatric patients compared to the proportion in 2017 (62%), suggesting that preparation strategies have improved for this patient category [14].

Besides preparedness in terms of medical knowledge and skills, psychological preparedness for deployment should not be underestimated. Humanitarian aid workers are exposed to significant stress through demanding working conditions, ethical and moral dilemmas, and confrontation with human suffering and violence [23, 29]. A survey among humanitarian workers found that 30% of the recently returned staff experienced significant symptoms of post-traumatic stress disorder [29]. Remarkably, in our questionnaire administered to ICRC medical personnel, most respondents reported that they experienced a low need for professional psychological support during their last deployment, and only one person received such help. Moreover, more than half of our respondents reported that, for future deployments, the availability of a mental health professional would be helpful to deal with stress. Perhaps the effectiveness of such an intervention was only realised in hindsight.

Other studies endorse the necessity of having a mental health professional available on site [30, 31], but even peer-to-peer contact resulted in a lower report of exhaustion during deployment [23, 32]. The British armed forces implemented a peer-to-peer Trauma Risk Management (TRiM) system, in which nonmedical but military-affiliated personnel are trained to screen service members on their risk of developing mental health conditions after traumatic incidents and provide customised support [33]. Furthermore, Surya et al. outlined other evidence-based strategies to reduce distress among deployed healthcare workers, such as team building, mindfulness, physical exercise, event-triggered counselling, eye movement desensitisation and reprocessing, and adequate predeployment training [34]. Following these recommendations, humanitarian aid organisations should develop comprehensive prevention and management strategies for deployed healthcare workers to cope with stress and mental illness while considering the potential, delayed manifestations.

This study is not without limitations. Specifically, the post-deployment questionnaire yielded a low response rate, and several variables have missing values, which requires careful interpretation. A one-time measurement might have resulted in a higher overall response rate, but the two time measurements focused on different deployment phases and could have reduced recall bias. Moreover, the three main professions of ICRC surgical teams (i.e. nurses, anaesthesiologists and surgeons) are sufficiently represented. Furthermore, we tried to mitigate social desirability bias by ensuring an anonymous response. Finally, this study administered a self-assessment questionnaire resulting in subjective measurements of the preparedness level for deployment, which could deviate from the factual level of preparedness. However, after a literature review on the self-assessment of surgical skills, Rizan et al. concluded that surgeons generally could accurately assess their own skills [35].

Despite these limitations, recommendations can be made for future deployment preparations based on our study findings in the context of the preceding literature. First, predeployment training should focus on the entire spectrum of medical professionals [36]. Greater efforts should be made to meet nurses’ training needs because a lower preparedness rating was already demonstrated in the previous survey in 2017 and other research in the broader context of disaster preparedness [37–40]. Within this study, nurses indicated several core medical topics to be included in future training (e.g. triage, burn treatment, and management of noncommunicable diseases in newborns), but their needs also lie in the field of interprofessional skills (e.g. communication, resource management or public health interventions) [22, 36, 41, 42]. Examples of promising training programmes for humanitarian health workers have been presented and can be built upon to develop future disaster preparedness curricula [43–46].

Second, onboarding missions are of immense value to provide insight into what is expected in terms of knowledge and skills, and these should be provided for healthcare workers, specifically those without previous deployment experience. Alternatively, or complementarily to an onboarding mission, additional work experience in a high-volume trauma centre is regarded as helpful preparation for deployment to a conflict zone.

Third, medical professionals’ background in training and experience should be considered during the composition of medical teams for deployment, ensuring a representation of complementary skills. Competency lists have been established for deployed teams providing damage control surgery in austere environments [9, 47], and an essential addition was made by Stathakarou et al*.,* who included nontechnical competencies [48]. Application of a competency checklist before deployment facilitates identifying the need for additional customised training or simultaneous deployment of medical professionals with complementary skills. Of course, basic training requirements (e.g. ATLS course attendance for physicians) are preserved but can be complemented with more specialised trauma care courses, such as the ICRC war surgery seminar or the Surgical Training for Austere Environments (STAE) course. Whether an applicant for humanitarian deployment holds the required competencies could be based on self-assessment or local supervisor assessment, or competency-based assessments, such as Entrustable Professional Activities (EPAs), could be incorporated into onboarding missions. However, the latter can be challenging due to the unpredictable environment. Objective measurement of required competencies for surgical team members deployed to austere environments is an important focus for future research.

Finally, humanitarian organisations should strengthen the peer-to-peer counselling network or even provide professional counselling during deployment. Awareness should already be raised predeployment regarding the provided strategies to cope with deployment-related stress and mental illness. These recommendations should be widely considered within the international humanitarian community as it is common for humanitarian healthcare workers to deploy with multiple organisations, as demonstrated by the finding that 30% of the respondents had been previously deployed with Médecins Sans Frontières.

## Conclusion

Recently deployed ICRC medical personnel reported feeling sufficiently prepared for their tasks during missions. However, a lower feeling of preparedness was found compared to 2017, combined with a persistent lower feeling of preparedness among nurses compared to physicians, which reconfirms the need for additional customised training of the entire spectrum of humanitarian healthcare workers. Onboarding missions or additional work experience in a high-volume trauma centre were identified as essential components in predeployment preparations. Additionally, recommendations were made regarding the topics that should be included in future training. The humanitarian medical aid community should consider competency-based customisation of training and composition of deployment teams, guided by research efforts to objectively identify and measure the required competencies. Continuous monitoring of humanitarian healthcare workers’ training needs is essential because substantial changes can occur related to changes in the environment, caseload or resources. 

## Supplementary Information

**Additional file 1.** Pre- and post-deployment questionnaires.

**Additional file 2.** Statistical analysis. Description: extensive analysis of all available data.

## Data Availability

All data generated or analysed during this study are included in this published article and its additional information files.
