# Supplementary material for: Self-perceived preparedness and training needs of healthcare personnel on humanitarian mission: a pre- and post-deployment survey
Source: World J Emerg Surg. 2022 Mar 5;17:14. doi: 10.1186/s13017-022-00417-z (PMC8898429; doi:10.1186/s13017-022-00417-z)
Supplement: Supplementary file 1 — Additional file 1. Pre- and post-deployment questionnaires. [file 13017_2022_417_MOESM1_ESM.docx]

**Additional file 1 – pre- and post-deployment questionnaires**

**Table of content**

[1 Pre-deployment questionnaire 1](#_Toc93256865)

[**General information** 1](#_Toc93256866)

[**Pre-deployment preparations** 4](#_Toc93256867)

[2 Post-deployment questionnaire 11](#_Toc93256868)

[**Deployment experiences** 11](#_Toc93256869)

[**Personal impact** 16](#_Toc93256870)

# Pre-deployment questionnaire

## **General information**

1. Gender
   1. Male
   2. Female
2. Age (years):
3. Country where you have completed most of your education:
4. What is your motivation to work for the ICRC? (Multiple answers possible)
   1. Career opportunities
   2. I was looking for an adventure
   3. It gives me a sense of purpose
   4. It is my moral duty
   5. Religious beliefs
   6. The additional clinical experience
   7. The income
   8. The opportunity to work together with colleagues from different nationalities
   9. Wanting a change from daily work
   10. Wanting to help people afflicted by war and disaster
   11. Wanting to help people less fortunate in healthcare options
   12. Wanting to work abroad
   13. Other (please specify):

Explanation:

1. Highest level of training:
   1. Anesthesiologist
   2. General practitioner
   3. Emergency Room physician
   4. Infectiologist
   5. Military physician
   6. (Registered) nurse
      1. Department/position:
         1. Emergency Department nurse
         2. Intensive Care Unit nurse
         3. Military nurse
         4. Surgical ward nurse
         5. Operation theatre nurse
         6. Teaching nurse
         7. Ward nurse
         8. Other (specify)
   7. Physiotherapist
   8. Gynecologist/obstetrician
   9. Surgeon
      1. General surgeon
      2. Orthopedic surgeon
      3. Plastic surgeon
      4. Trauma surgeon
      5. Urologist
      6. Vascular surgeon
      7. Gastrointestinal surgeon
   10. Other (specify)
2. [NURSES] Date (year) of official registration as a nurse:
3. [PHYSIOTHERAPISTS] Date (year) of official registration as a physiotherapist:
4. [PHYSIOTHERAPISTS] Date (year) of master’s degree:
5. [PHYSICIANS] Date (year) of master’s degree:
6. [PHYSICIANS] Date (year) of board registration as a medical specialist (if applicable):
7. Years of clinical experience in your field of expertise since your official registration in your profession:
8. Experience with pediatric trauma during the last 2 years:
   1. Not involved in any pediatric surgical procedures
   2. Sporadically involved in pediatric surgical procedures
   3. Involved in pediatric surgical procedures on a monthly basis
   4. Involved in pediatric surgical procedures on a weekly basis
   5. Involved in pediatric surgical procedures on a daily basis
   6. Not applicable
9. How many deployments have you done with the ICRC?
   1. No deployments yet
   2. 1
   3. 2
   4. 3
   5. 4
   6. 5 +
10. To which mission will your upcoming ICRC deployment be?
11. How many deployments have you done with the armed forces?
    1. 0
    2. 1
    3. 2
    4. 3
    5. 4
    6. 5+
12. How many deployments have you done with Médecins Sans Frontières (MSF)?
    1. 0
    2. 1
    3. 2
    4. 3
    5. 4
    6. 5+
13. How many deployments have you done with other organizations than the ICRC, MSF or the armed forces?
    1. 0
    2. 1
    3. 2
    4. 3
    5. 4
    6. 5+

Please specify with which organizations you were deployed:

## **Pre-deployment preparations**

1. [NURSES] Which of the following courses did you attend as a participant (or instructor), and in which year did you attend it?
   1. Advanced Burn Life Support (ABLS)
   2. Advanced Cardiovascular Life Support (ACLS)
   3. Advanced Life Support (ALS)
   4. Advanced Medical Life Support (AMLS)
   5. Advanced Trauma Life Support (ATLS) as observer
   6. Anesthesia, Trauma and Critical Care (ATACC) course as observer
   7. Battlefield Advanced Trauma Life Support (BATLS) as observer
   8. Definitive Surgical Trauma Care (DSTC) Course
   9. Definitive Anaesthetic Trauma Care (DATC) Course
   10. Diploma of Tropical Nursing
   11. Emergency Nursing Pediatric Course (ENPC)
   12. European Paediatric Advanced Life Support (EPALS)
   13. European Trauma Course (ETC)
   14. ICRC Health Emergencies in Large Populations (HELP) course
   15. ICRC two-month onboarding mission
   16. ICRC War Surgery Seminar
   17. Immediate Life Support (ILS)
   18. Medical Response to Major Incidents & Disasters (MRMI-course)
   19. Military Operational Surgical Training (MOST)
   20. Prehospital Trauma Life Support (PHTLS)
   21. Trauma Nursing Core Course (TNCC)
   22. Wilderness Advanced Life Support (WALS)
   23. None
   24. Other (specify):
2. [PHYSIOTHERAPISTS] Which of the following courses did you attend as a participant (or instructor), and in which year did you attend it?
   1. UK Department for International Development (DFID) overseas rehabilitation course
   2. Preparation course via Médecins Sans Frontières (MSF)
   3. Handicap International
   4. Public Health modules (e.g. STPH, CERAH)
   5. ICRC HELP course
   6. ICRC two-month onboarding mission
   7. ICRC War Surgery Seminar
   8. None
   9. Other (specify):
3. [PHYSICIANS] Which of the following courses did you attend as a participant (or instructor), and in which year did you attend it?
   1. Advanced Burn Life Support (ABLS)
   2. Advanced Cardiovascular Life Support (ACLS)
   3. Advanced Life Support (ALS)
   4. Advanced Medical Life Support (AMLS)
   5. Advanced Paediatric Life Support (APLS)
   6. Advanced Surgical Skills for Exposure in Trauma (ASSET)
   7. Advanced Trauma Life Support (ATLS)
   8. Advanced Trauma Operative Management (ATOM)
   9. Anesthesia, Trauma and Critical Care (ATACC) course
   10. Battlefield Advanced Trauma Life Support (BATLS)
   11. Definitive Surgical Trauma Care (DSTC) Course
   12. Definitive Anaesthetic Trauma Care (DATC) Course
   13. Definitive Surgical Trauma Skills (DSTS)
   14. Emergency Management of the Severe Burns Course (EMSB)
   15. Emergency War Surgery Course (EWSC)
   16. European Paediatric Advanced Life Support (EPALS)
   17. European Trauma Course (ETC)
   18. Health Emergencies in Large Populations (HELP) course
   19. ICRC two-month onboarding mission
   20. ICRC War Surgery Seminar
   21. Immediate Life Support (ILS)
   22. Major Incident Medical Management and Support course (MIMMS)
   23. Military Operational Surgical Training (MOST)
   24. Medical Response to Major Incidents & Disasters (MRMI-course)
   25. Prehospital Trauma Life Support (PHTLS)
   26. Surgical Training for Austere Environments (STAE)
   27. Wilderness Advanced Life Support (WALS)
   28. None
   29. Other (specify):
4. How would you value each course you attended, with regard to your general preparation and your preparation for pediatric trauma (rehabilitation)?
   1. Rating for my general preparation:
      1. Not important at all
      2. -
      3. -
      4. -
      5. Absolutely essential
      6. No opinion
   2. Rating for my preparation for pediatric trauma (rehabilitation):
      1. Not important at all
      2. -
      3. -
      4. -
      5. Absolutely essential
      6. No opinion
5. Date (year) of most recent (refresher) course concerning trauma care, emergency care or advanced life support (if applicable):
6. Date (year) of your onboarding mission (if applicable):
7. Did you, as preparation for deployment, participate in a clinical placement in a trauma center in an area with high rates of severe trauma injuries (expected to be somewhat similar as injuries seen on deployment)?
   1. Yes
   2. No
   3. N/A

Explanation:

1. Did you, as preparation for deployment, get the chance to practice with the equipment you had to your disposal during deployment?
   1. Yes
   2. No
   3. N/A

Explanation:

1. What do you value most for your ICRC deployment preparations? (Please enter your top 3):
   1. The basic/minimal education program for my current profession
   2. Training on Crew Resource Management (CRM) / team dynamics
   3. Clinical placement in a trauma center in an area with high rates of severe trauma injuries (expected to be somewhat similar as injuries seen on deployment)
   4. Military training courses on trauma care, emergency care or advanced life support
   5. Other (civilian) training courses on trauma care, emergency care or advanced life support
   6. Previous emergency care experience
   7. Previous deployments
   8. ICRC onboarding mission
   9. Information on the practical aspects of deployment
   10. Getting familiar with the equipment you will have to your disposal during deployment
   11. Other (please specify):

Explanation:

*Example of answer options question 9:*

*Attended? Rank of importance (top 3)*

*a. yes/no No 3*

*c. yes/no No 2*

*d. yes/no No 1*

1. [NURSES] Please mark the topic(s) in which you would like to receive additional training prior to deployment, and please specify the reason why. You are allowed to mark multiple topics.
   1. Care of patients with traction devices or external fixators
   2. Care of the burns patient
   3. Care of the obstetric patient
   4. Care of the patient with neurotrauma
   5. Care of the pediatric patient
   6. ICU care
   7. Newborn NCD management
   8. Pain management
   9. Tourniquet application
   10. Triage and mass casualty management
   11. Tropical disease management
   12. Wound care
   13. Other (please specify):
   14. I did not feel the need for additional training

Explanation:

1. [PHYSIOTHERAPISTS] Please mark the topic(s) in which you would like to receive additional training prior to deployment, and please specify the reason why. You are allowed to mark multiple topics.
   1. Amputee rehabilitation
   2. Burns rehabilitation
   3. Neurorehabilitation
   4. Musculoskeletal rehabilitation
   5. Cardiorespiratory rehabilitation
   6. Maxillofacial surgery rehabilitation
   7. Pediatric rehabilitation of children
   8. Care of patients with traction devices or external fixators
   9. Pain management
   10. Triage and mass casualty management
   11. Wound related rehabilitation
   12. Other (please specify):
   13. I did not feel the need for additional training
2. [PHYSICIANS] Please mark the topic(s) in which you would like to receive additional training prior to deployment, and please specify the reason why. You are allowed to mark multiple topics.
   1. Amputation techniques
   2. Antibiotic selection/management
   3. Burn treatment
   4. Fracture surgery
   5. Gastrointestinal surgery
   6. Hand surgery
   7. ICU care
   8. Maxillofacial surgery
   9. Neurosurgery
   10. Nerve repair techniques
   11. Obstetrics/Gynecology
   12. Ophthalmic surgery
   13. Pediatrics
   14. Pediatric surgery
   15. Plastic (reconstructive) surgery
   16. Resuscitation
   17. Sonography/Ultrasound skills
   18. Soft tissue surgery
   19. (Surgical) decision making
   20. Triage skills
   21. Tropical diseases
   22. Thorax surgery
   23. Urology
   24. Vascular surgery
   25. Other (please specify):
   26. I did not feel the need for additional training

Why do you feel the need for additional training on this topic?

Because I do not feel optimally prepared for this topic on deployment

I do feel prepared for this topic on deployment, but I would find it an interesting topic for additional training

- 1. Other:

1. Prior to your deployment with the ICRC, were you adequately informed about:
   1. Your scope of practice
      1. Not at all
      2. -
      3. -
      4. -
      5. More than sufficient
      6. I do not remember/No opinion:
   2. The current situation in the mission area
      1. Not at all
      2. -
      3. -
      4. -
      5. More than sufficient
      6. I do not remember/No opinion:
   3. The local environmental challenges
   4. The local living conditions
   5. Your duties and responsibilities
   6. The field facilities and equipment available
   7. Means of contact with your home/family during deployment
   8. The other ICRC participants of the mission and their responsibilities
   9. The nature and severity of the injuries of patients you could face during deployment

Explanation:

1. Are you pro or con a cooperation with the armed forces in pre-deployment training?
   1. Pro, because:
   2. Con, because:
   3. No opinion
2. When was the last time you have worked in a regular hospital in your home country?
   1. Up until the moment of deployment
   2. Between 1 to 6 months before deployment
   3. Between 6 months to 1 year before deployment
   4. More than 1 year before deployment
   5. I have not worked in a regular hospital
3. Do you feel professionally prepared to treat/care for pediatric trauma patients during your upcoming deployment?
   1. Very unprepared
   2. -
   3. -
   4. -
   5. More than sufficient
   6. I do not remember/No opinion

Explanation:

1. Do you feel professionally prepared to treat/care for adult trauma patients during your upcoming deployment?
   1. Very unprepared
   2. -
   3. -
   4. -
   5. More than sufficient
   6. I do not remember/No opinion

Explanation:

# Post-deployment questionnaire

## **Deployment experiences**

The following questions refer to your last deployment with the ICRC.

1. How much time, on average, did you spend on call in one day?
   1. Less than 8 hours
   2. 8 to 10 hours
   3. 10 to 12 hours
   4. 12 to 14 hours
   5. More than 14 hours
   6. Constant (24 hours a day, 7 days a week)
   7. I do not remember

Explanation:

1. How many days, on average, did you spend on call in one week?
   1. Less than 5 days
   2. 5 days
   3. 6 days
   4. 7 days
   5. I do not remember

Explanation:

1. Caseload range during last ICRC deployment:
   1. None
   2. <1 per week
   3. 1-20 per week
   4. 21-40 per week
   5. 41-60 per week
   6. 61-80 per week
   7. 81-100 per week
   8. >100 per week
   9. I do not remember; N/A

Explanation:

1. How frequently were you exposed to pediatric trauma during your last ICRC deployment?
   1. Less than once a month
   2. Once a month
   3. A few times a month
   4. Once a week
   5. A few times a week
   6. Every day
   7. I do not remember

Explanation:

1. How frequently were you exposed to adult trauma during your last ICRC deployment?
   1. Less than once a month
   2. Once a month
   3. A few times a month
   4. Once a week
   5. A few times a week
   6. Every day
   7. I do not remember

Explanation:

1. How frequently were you involved in the management of injuries outside your field of specialization?
   1. Less than once a month
   2. Once a month
   3. A few times a month
   4. Once a week
   5. A few times a week
   6. Every day
   7. I do not remember; N/A

Explanation:

1. Were your pre-deployment training, knowledge and skills sufficient regarding the injuries you have treated during your last ICRC deployment?
   1. Very insufficient
   2. -
   3. -
   4. -
   5. More than sufficient
   6. I do not remember/No opinion

Explanation:

1. In general, how do you consider the medical training, knowledge and skills of your direct colleagues during your last ICRC deployment?
   1. Very poor
   2. -
   3. – (Average)
   4. -
   5. Excellent
   6. I do not remember/No opinion

Explanation:

1. How do you rate your confidence in your skills regarding the treatment of/care for pediatric patients compared to the treatment of/care for adult patients during your last ICRC deployment?
   1. Much more confident in treating adult patients
   2. -
   3. – (Equally confident)
   4. -
   5. Much more confident in treating pediatric patients
   6. I do not remember/No opinion

Explanation:

In the following locations, what did you think about the equipment you had to your disposal to treat/care for adult patients during your last ICRC deployment?

- 1. Prehospital:

Very dissatisfied

-

- (Neutral)

-

Very satisfied

- - 1. Not applicable
  1. In the emergency room
  2. In the operation room
  3. In the intensive care unit
  4. During follow-up

Explanation:

1. In the following locations, what did you think about the equipment you had to your disposal to treat/care for pediatric patients during your last ICRC deployment?
   1. Prehospital:

Very dissatisfied

-

- (Neutral)

-

Very satisfied

- - 1. Not applicable
  1. In the emergency room
  2. In the operation room
  3. In the intensive care unit
  4. During follow-up

Explanation:

1. Were you able to consult more experienced colleagues when needed? (Multiple answers possible)
   1. Yes, on site
   2. Yes, by phone
   3. Yes, by internet
   4. Yes, other (please specify):
   5. No, it was not possible at the right moments
   6. No, it was never possible
   7. I do not remember; N/A

Explanation:

1. Which structured means of communication were used to transfer information about patients from other medical services to the emergency ward personnel? (Multiple answers possible)
   1. MIST (Mechanism of injury, Injuries sustained, Signs, Treatment and Trends in the vital signs)
   2. SBAR (Situation, Background, Assessment, Recommendation)
   3. RSVP (Reason, Story, Vital signs, Plan)
   4. No systematic methods were used
   5. There was no communication with other medical services
   6. Other (specify):
   7. I do not remember

Explanation:

1. [PHYSIOTHERAPISTS] Was a higher level of care or tertiary center available for your patients? (Multiple answers possible)
   1. Yes, for pediatric patients
   2. Yes, for adult patients
   3. No
   4. I do not remember; N/A

Explanation:

1. [PHYSICIANS] Was a referral center available for your patients? (Multiple answers possible)
   1. Yes, for pediatric patients
   2. Yes, for adult patients
   3. No
   4. I do not remember; N/A

Explanation:

1. [PHYSIOTHERAPISTS] How fast could a patient arrive at the nearest referral center? (with the quickest mode of transportation)
   1. For pediatric patients:
      1. Less than one hour
      2. More than one hour
      3. More than two hours
      4. I do not remember; N/A

Explanation:

- 1. For adult patients:
     1. Less than one hour
     2. More than one hour
     3. More than two hours
     4. I do not remember; N/A

Explanation:

1. [PHYSICIANS] How fast could a patient arrive at the nearest referral center? (with the quickest mode of transportation)
   1. For pediatric patients:
      1. Less than one hour
      2. More than one hour
      3. More than two hours
      4. I do not remember; N/A

Explanation:

- 1. For adult patients:
     1. Less than one hour
     2. More than one hour
     3. More than two hours
     4. I do not remember; N/A

Explanation:

1. In hindsight, how would you rate your preparedness prior to deployment to treat/care for pediatric trauma patients?
   1. Very unprepared
   2. -
   3. -
   4. -
   5. More than sufficient
   6. I do not remember/No opinion

Explanation:

1. In hindsight, how would you rate your preparedness prior to deployment to treat/care for adult trauma patients?
   1. Very unprepared
   2. -
   3. -
   4. -
   5. More than sufficient
   6. I do not remember/No opinion

Explanation:

1. What impact did your last ICRC deployment have on your trauma management skills? Your trauma management skills:
   1. Much deteriorated
   2. -
   3. – (Did not change)
   4. -
   5. Much improved
   6. No opinion

Explanation:

1. What impact did your last ICRC deployment have on your skills in your primary specialism (in the non-deployed setting)? Your skills:
   1. Much deteriorated
   2. -
   3. – (Did not change)
   4. -
   5. Much improved
   6. No opinion

Explanation:

## **Personal impact**

The following questions refer to your last deployment with the ICRC.

1. Did you feel the need for a consistent contact person (peer-to-peer / colleague) on site, to talk about your experiences during your last ICRC deployment?
   1. Not at all
   2. -
   3. – (Undecided)
   4. -
   5. Very much
   6. I do not remember/No opinion

Explanation:

1. Did you, in fact, talk to a consistent contact person on site about your experiences during your last ICRC deployment?
   1. Yes
   2. No

Explanation:

1. Did you ever feel the need to debrief (in any form) within the team on your experiences during your last ICRC deployment?
   1. Not at all
   2. -
   3. – (Undecided)
   4. -
   5. Very much
   6. I do not remember/No opinion

Explanation:

1. Did you, in fact, debrief within the team on your experiences during your last ICRC deployment?
   1. Yes
   2. No

Explanation:

1. Did you ever feel the need for professional psychological help during your last ICRC deployment?
   1. Not at all
   2. -
   3. – (Undecided)
   4. -
   5. Very much
   6. I do not remember/No opinion

Explanation:

1. Did you, in fact, get professional psychological help during your last ICRC deployment?
   1. Yes
   2. No

Explanation:

1. Were you adequately informed about who to contact for professional psychological help, should you require it:
   - 1. Not at all
     2. -
     3. -
     4. -
     5. More than sufficient
     6. I do not remember/No opinion

Explanation:

1. What would be helpful to deal with the stress during a deployment? (Multiple answers possible)
   1. Stress management training beforehand
   2. A card/kit with stress management advice for members to carry
   3. Availability of a mental health professional during deployment
   4. Briefing about strategies relevant to deployment before departure
   5. Follow-up of individual ICRC team members
   6. Online social support network
   7. Other (please specify):

Explanation:

1. How do you value formal debriefing with the ICRC headquarter after deployment?
   1. Not important at all
   2. –
   3. – (Neutral)
   4. –
   5. Absolutely essential
   6. No opinion

Explanation:

1. How do you value informal debriefing after deployment (with colleagues, friends or family)?
   1. Not important at all
   2. –
   3. – (Neutral)
   4. –
   5. Absolutely essential
   6. No opinion

Explanation:

1. What effect did your last ICRC deployment have on your personal development?
   1. Major negative effect
   2. -
   3. – (Neutral)
   4. -
   5. Major positive effect
   6. No opinion

Explanation:

1. What effect did your last ICRC deployment have on your private situation at home?
   1. Major negative effect
   2. -
   3. – (Neutral)
   4. -
   5. Major positive effect
   6. No opinion

Explanation:

1. Regarding preparation (e.g. courses or training), what would you especially recommend for colleagues preparing for a mission in a conflict zone?
