# Supplementary material for: Self-perceived preparedness and training needs of healthcare personnel on humanitarian mission: a pre- and post-deployment survey
Source: World J Emerg Surg. 2022 Mar 5;17:14. doi: 10.1186/s13017-022-00417-z (PMC8898429; doi:10.1186/s13017-022-00417-z)
Supplement: Supplementary file 2 — Additional file 2. Statistical analysis. Description: extensive analysis of all available data. [file 13017_2022_417_MOESM2_ESM.docx]

**Additional file 2 - statistical analysis**

**Table of contents**

* indicating a statistically significant difference in this analysis

[1 DESCRIPTIVE STATISTICS 4](#_Toc95310167)

[1.1 Response rate 4](#_Toc95310168)

[1.2 Sub specialization of nurses and surgeons 5](#_Toc95310169)

[1.3 Number of previous deployments 6](#_Toc95310170)

[1.4 Country of primary medical education 7](#_Toc95310171)

[1.5 Last time worked in hospital in home country 9](#_Toc95310172)

[1.6 Experience with paediatric trauma during last two years 9](#_Toc95310173)

[1.7 Motivation to work with the ICRC 9](#_Toc95310174)

[1.8 Pre-deployment courses attended 10](#_Toc95310175)

[1.9 Topics requested for additional training 12](#_Toc95310176)

[1.10 Pre-deployment rating of self-perceived preparedness to treat paediatric and adult patients 14](#_Toc95310177)

[1.11 Rating of confidence in treating adult versus paediatric patients during deployment 14](#_Toc95310178)

[1.12 Rating of pre-deployment preparation activities 15](#_Toc95310179)

[1.13 Rating of pre-deployment information about the mission 16](#_Toc95310180)

[1.14 Post-deployment rating of self-perceived preparedness to treat paediatric and adult patients 17](#_Toc95310181)

[1.15 Time spent on call 18](#_Toc95310182)

[1.16 Injuries encountered during deployment 19](#_Toc95310183)

[1.17 Satisfaction with equipment during deployment 20](#_Toc95310184)

[1.18 Availability of more experienced colleagues 20](#_Toc95310185)

[1.19 Availability of referral centre 20](#_Toc95310186)

[1.20 Structured methods of communication during deployment 21](#_Toc95310187)

[1.21 Deployment impact on trauma management skills 22](#_Toc95310188)

[1.22 Deployment impact on skills in primary specialism 22](#_Toc95310189)

[1.23 Deployment impact on personal development 22](#_Toc95310190)

[1.24 Deployment impact on private situation 22](#_Toc95310191)

[1.25 Need for peer-to-peer contact during deployment 23](#_Toc95310192)

[1.26 Need for debriefing during deployment 23](#_Toc95310193)

[1.27 Need for professional psychological help during deployment 23](#_Toc95310194)

[1.28 Actual peer-to-peer contact, debriefing, and professional psychological help received 23](#_Toc95310195)

[1.29 Activities helpful to deal with stress during deployment 24](#_Toc95310196)

[2 COMPARATIVE STATISTICS 25](#_Toc95310197)

[**2.1** **Grouping variable: profession (physician/nurse)** 25](#_Toc95310198)

[2.1.1 Weeks of deployment with ICRC 25](#_Toc95310199)

[2.1.2 Years of clinical experience 26](#_Toc95310200)

[2.1.3 Pre-deployment rating of self-perceived preparedness to treat paediatric patients* 27](#_Toc95310201)

[2.1.4 Pre-deployment rating of self-perceived preparedness to treat adult patients* 28](#_Toc95310202)

[2.1.5 Number of topics requested for additional training 29](#_Toc95310203)

[2.1.6 Rating of pre-deployment training, knowledge and skills regarding injuries treated during deployment* 30](#_Toc95310204)

[2.1.7 Rating of pre-deployment training, knowledge and skills of colleagues* 31](#_Toc95310205)

[2.1.8 Rating of equipment to treat adult patients during deployment* 32](#_Toc95310206)

[2.1.9 Rating of equipment to treat paediatric patients during deployment 34](#_Toc95310207)

[2.1.10 Post-deployment rating of self-perceived preparedness to treat paediatric patients* 36](#_Toc95310208)

[2.1.11 Post-deployment rating of self-perceived preparedness to treat adult patients* 37](#_Toc95310209)

[2.1.12 Need for peer-to-peer contact 38](#_Toc95310210)

[2.1.13 Need for debriefing 39](#_Toc95310211)

[2.1.14 Need for professional psychological help 40](#_Toc95310212)

[2.1.15 Impact of deployment on personal development 41](#_Toc95310213)

[2.1.16 Impact of deployment on private situation 42](#_Toc95310214)

[**2.2** **Grouping variable: profession (surgeon/anesthesiologist/nurse)** 43](#_Toc95310215)

[2.2.1 Weeks of deployment with the ICRC 43](#_Toc95310216)

[2.2.2 Years of clinical experience 44](#_Toc95310217)

[2.2.3 Overview of pre- and post-deployment rating of self-perceived preparedness to treat paediatric and adult patients 45](#_Toc95310218)

[2.2.4 Pre-deployment rating of self-perceived preparedness to treat paediatric patients 46](#_Toc95310219)

[2.2.5 Pre-deployment rating of self-perceived preparedness to treat adult patients 47](#_Toc95310220)

[2.2.6 Number of topics requested for additional training* 48](#_Toc95310221)

[2.2.7 Rating of pre-deployment training, knowledge and skills regarding injuries treated during deployment* 50](#_Toc95310222)

[2.2.8 Rating of pre-deployment training, knowledge and skills of colleagues 52](#_Toc95310223)

[2.2.9 Rating of equipment to treat adult patients during deployment 53](#_Toc95310224)

[2.2.10 Rating of equipment to treat paediatric patients during deployment 59](#_Toc95310225)

[2.2.11 Post-deployment rating of self-perceived preparedness to treat paediatric and adult patients 65](#_Toc95310226)

[2.2.12 Need for peer-to-peer contact, debriefing or professional psychological help during deployment 68](#_Toc95310227)

[2.2.13 Impact of deployment on personal development and private situation 72](#_Toc95310228)

[**2.3** **Grouping variable: clinical placement (yes/no)** 75](#_Toc95310229)

[2.3.1 Pre-deployment rating of self-perceived preparedness to treat paediatric and adult patients* 75](#_Toc95310230)

[2.3.2 Post-deployment rating of self-perceived preparedness to treat paediatric and adult patients 77](#_Toc95310231)

[2.3.3 Need for peer-to-peer contact, debriefing, and professional psychological help during deployment* 79](#_Toc95310232)

[**2.4** **Grouping variable: previous deployments (yes/no)** 81](#_Toc95310233)

[2.4.1 Pre-deployment rating of self-perceived preparedness to treat paediatric and adult patients 81](#_Toc95310234)

[2.4.2 Post-deployment rating of self-perceived preparedness to treat paediatric and adult patients 82](#_Toc95310235)

[2.4.3 Number of topics requested for additional training* 83](#_Toc95310236)

[**2.5** **Grouping variable: onboarding mission (yes/no)** 84](#_Toc95310237)

[2.5.1 Pre-deployment rating of self-perceived preparedness to treat paediatric and adult patients 84](#_Toc95310238)

[2.5.2 Post-deployment rating of self-perceived preparedness to treat paediatric and adult patients 85](#_Toc95310239)

[2.5.3 Number of topics requested for additional training* 86](#_Toc95310240)

[**2.6** **Grouping variable: country of education (low/middle/high income)** 87](#_Toc95310241)

[2.6.1 Pre-deployment rating of self-perceived preparedness to treat paediatric and adult patients 87](#_Toc95310242)

[2.6.2 Post-deployment rating of self-perceived preparedness to treat paediatric and adult patients 89](#_Toc95310243)

[2.6.3 Rating of equipment to treat adult and paediatric patients during deployment* 91](#_Toc95310244)

[2.6.4 Rating of equipment to treat paediatric patients during deployment* 97](#_Toc95310245)

[**2.7** **Grouping variable: matched or unmatched responses (dropouts)** 104](#_Toc95310246)

[2.7.1 Continuous variables* 104](#_Toc95310247)

[2.7.2 Gender 108](#_Toc95310248)

[2.7.3 Country of primary medical education 109](#_Toc95310249)

[2.7.4 Profession 111](#_Toc95310250)

[2.7.5 Previous deployments 112](#_Toc95310251)

[2.7.6 Deployment preparations 117](#_Toc95310252)

[2.7.7 Last time worked in regular hospital 118](#_Toc95310253)

[**2.8** **Correlation with: years of clinical experience** 119](#_Toc95310254)

[2.8.1 Pre-deployment rating of self-perceived preparedness to treat paediatric and adult patients 119](#_Toc95310255)

[2.8.2 Post-deployment rating of self-perceived preparedness to treat paediatric and adult patients 119](#_Toc95310256)

# DESCRIPTIVE STATISTICS

## Response rate

Table 1. Response rate

| **Group** | **N (%)** |
| --- | --- |
| Pre-deployment questionnaires  Completely filled out  Partially filled out  Total | 87 (40.1%)  27 (12.4%)  114 (52.5%) |
| Pre-deployment questionnaires  Physicians  Nurses  Missing | 72 (63.2%)  40 (35.1%)  2 (1.8%) |
| Pre-deployment questionnaires  Surgeons  Anesthesiologists  Nurses  Other  Missing | 42 (19.4%)  25 (11.5%)  40 (18.4%)  5 (2.3%)  2 (0.9%) |
| Post-deployment questionnaires  Completely filled out  Partially filled out  Total | 55 (25.3%)  3 (1.4%)  58 (26.7%) |
| Post-deployment questionnaires  Physician  Nurse  Missing | 32 (55.2%)  25 (43.1%)  1 (1.7%) |
| Post-deployment questionnaires  Surgeons  Anesthesiologists  Nurses  Other  Profession missing | 23 (10.6%)  7 (3.2%)  25 (11.5%)  3 (1.4%)  0 (0.0%) |

## Sub specialization of nurses and surgeons

Table 2. Sub specialization nurses

| **Sub specialization** | **N (%)** |
| --- | --- |
| ED nurse | 5 (12.5%) |
| ICU nurse | 4 (10.0%) |
| OT nurse | 9 (22.5%) |
| Surgical ward nurse | 2 (5.0%) |
| Teaching nurse | 2 (5.0%) |
| Ward nurse | 7 (17.5%) |
| Other | 3 (7.5%) |
| Missing | 8 (20.0%) |

*ED Emergency Department; ICU Intensive Care Unit; OT Operation Theatre*

Table 3. Sub specialization surgeons

| **Sub specialization** | **N (%)** |
| --- | --- |
| General surgeon | 29 (69.0%) |
| Orthopaedic surgeon | 3 (7.1%) |
| Trauma surgeon | 3 (7.1%) |
| Vascular surgeon | 1 (2.4%) |
| Paediatric surgeon | 2 (4.8%) |
| GI and general surgeon | 1 (2.4%) |
| Trauma and orthopaedic surgeon | 1 (2.4%) |
| Other (surgeon with certification in trauma surgery) | 1 (2.4%) |
| Missing | 1 (2.4%) |

## Number of previous deployments

Table 4. Number of previous deployments

| **Deployment organization** | **Value** |
| --- | --- |
| ICRC, weeks of deployment (median, IQR)  Missing | 40 (107.0)  33 (28.9%) |
| ICRC, number of deployments (N, %)  0  1  2  3  4  5 or more  Missing | 24 (21.1%)  15 (13.2%)  14 (12.3%)  12 (10.5%)  7 (6.1%)  36 (31.6%)  6 (5.3%) |
| MSF, weeks of deployment (median, IQR)  Missing | 33.5 (106.2)  76 (66.7%) |
| MSF, number of deployments (N, %)  0  1  2  3  4  5 or more  Missing | 68 (59.6%)  7 (6.1%)  6 (5.3%)  7 (6.1%)  2 (1.8%)  17 (14.9%)  7 (6.1%) |
| Armed forces, weeks of deployment (median, IQR)  Missing | 55 (101.7)  94 (82.5%) |
| Armed forces, number of deployments (N, %)  0  1  2  3  4  5 or more  Missing | 88 (77.2%)  7 (6.1%)  4 (3.5%)  1 (0.9%)  0 (0.0%)  8 (7.0%)  6 (5.3%) |
| Other, weeks of deployment (median, IQR)  Missing | 40 (80.0)  59 (51.8%) |
| Other, number of deployments (N, %)  0  1  2  3  4  5 or more  Missing | 50 (43.9%)  23 (20.2%)  12 (10.0%)  2 (1.8%)  3 (2.6%)  17 (14.9%)  7 (6.1%) |

*ICRC International Committee of the Red Cross*

*MSF Médecins Sans Frontières*

*IQR Interquartile range*

## Country of primary medical education

Table 5. Country of primary medical education

| **Country** | **Frequency** |
| --- | --- |
| Algeria ( | 4 (3.5%) |
| Argentina | 2 (1.8%) |
| Australia | 3 (2.6%) |
| Brazil (Brasil) | 2 (1.8%) |
| Burkina Faso | 1 (0.9%) |
| Burundi (Uburundi) | 2 (1.8%) |
| Canada | 5 (4.4%) |
| Congo (DRC) (Jamhuri ya Kidemokrasia ya Kongo) | 1 (0.9%) |
| Cuba | 1 (0.9%) |
| Denmark (Danmark) | 1 (0.9%) |
| Egypt ( | 4 (3.5%) |
| El Salvador | 1 (0.9%) |
| Ethiopia | 2 (1.8%) |
| Finland (Suomi) | 1 (0.9%) |
| France | 6 (5.3%) |
| Germany (Deutschland) | 4 (3.5%) |
| Ghana (Gaana) | 1 (0.9%) |
| Haiti | 1 (0.9%) |
| Hong Kong (香港) | 2 (1.8%) |
| India (भारत) | 3 (2.6%) |
| Israel ( | 1 (0.9%) |
| Italy (Italia) | 6 (5.3%) |
| Japan (日本) | 6 (5.3%) |
| Kenya | 6 (5.3%) |
| Mexico (México) | 3 (2.6%) |
| Netherlands (Nederland) | 7 (6.1%) |
| New Zealand | 1 (0.9%) |
| Nigeria | 1 (0.9%) |
| Norway (Norge) | 2 (1.8%) |
| Philippines | 2 (1.8% |
| Poland (Polska) | 1 (0.9%) |
| Portugal | 3 (2.6%) |
| Romania (România) | 1 (0.9%) |
| Russia (Россия) | 3 (2.6%) |
| Senegal (Sénégal) | 3 (2.6%) |
| Serbia (Србија) | 2 (1.8%) |
| Spain (España) | 1 (0.9%) |
| Switzerland (Schweiz) | 5 (4.4%) |
| Turkey (Türkiye) | 1 (0.9%) |
| Uganda | 1 (0.9%) |
| Ukraine (Україна) | 1 (0.9%) |
| United Kingdom | 7 (6.1%) |
| United States | 2 (1.8%) |
| Zimbabwe | 1 (0.9%) |
| Missing | 0 (0.0%) |

Table 6.Continent of primary medical education

| **Continent** | **N (%)** |
| --- | --- |
| Africa | 27 (20.8%) |
| Asia | 15 (11.5%) |
| Europe | 51 (39.2%) |
| Oceania | 4 (3.1%) |
| North America | 7 (5.4%) |
| South America | 10 (7.7%) |
| Missing | 16 (12.3%) |

## Last time worked in hospital in home country

Table 7. Last time worked in hospital in home country

| **Last time worked in hospital in home country** | **N (%)** |
| --- | --- |
| Up until the moment of deployment | 51 (44.7%) |
| Between 1 to 6 months before deployment | 10 (8.8%) |
| Between 6 months to 1 year before deployment | 5 (4.4%) |
| More than 1 year before deployment | 18 (15.8%) |
| I have not worked in a regular hospital | 3 (2.6%) |
| Missing | 27 (23.7%) |

## Experience with paediatric trauma during last two years

Table 8. Experience with paediatric trauma during last two years

| **Experience with paediatric trauma during the last two years** | **N (%)** |
| --- | --- |
| Not involved in any paediatric surgical procedures | 8 (7.0%) |
| Sporadically involved in paediatric surgical procedures | 41 (36.0%) |
| Involved in paediatric surgical procedures on a monthly basis | 22 (19.3% |
| Involved in paediatric surgical procedures on a weekly basis | 24 (21.1%) |
| Involved in paediatric surgical procedures on a daily basis | 13 (11.4%) |
| Missing | 6 (5.3%) |

## Motivation to work with the ICRC

Table 9. Motivation to work with the ICRC

| **Reason** | **N (%)** |
| --- | --- |
| Wanting to help people afflicted by war and disaster | 91 (79.8%) |
| Wanting to help people less fortunate in healthcare options | 81 (71.1%) |
| The opportunity to work together with colleagues from different nationalities | 66 (57.9%) |
| It gives me a sense of purpose | 51 (44.7%) |
| The additional clinical experience | 45 (39.5%) |
| Career opportunities | 35 (30.7%) |
| It is my moral duty | 26 (22.8%) |
| Wanting to work abroad | 23 (20.2%) |
| Wanting a change from daily work | 19 (16.7%) |
| The income | 17 (14.9%) |
| I was looking for an adventure | 8 (7.0%) |
| Religious beliefs | 3 (2.6%) |
| Missing | 0 (0.0%) |

## Pre-deployment courses attended

Table 10. Attended pre-deployment courses, stratified per profession

| **Course^$^** | **Attended**  **(N, %)^a^** | **N/A**  **(N, %)** | **Median rating for general preparation (IQR)** | **Missing** | **Median rating for preparation for paediatric trauma (IQR)** | **Missing** |
| --- | --- | --- | --- | --- | --- | --- |
| **SURGEONS (N=42)** | | | | | | |
| ABLS | 4 (9.5%) | 4 (9.5%) | 5.0 (0.8) | 38 (90.5%) | 5.0 (0.8) | 38 (90.5%) |
| ACLS | 6 (14.3%) | 4 (9.5%) | 4.5 (1.8) | 38 (90.5%) | 3.0 (3.0) | 37 (88.1%) |
| ALS | 4 (9.5%) | 4 (9.5%) | 3.5 (2.5) | 38 (90.5%) | 2.5 (3.8) | 38 (90.5%) |
| APLS | 1 (2.4%) | 4 (9.5%) | 3.0 (0.0) | 41 (97.6%) | 4.0 (0.0) | 41 (97.6%) |
| ASSET | 2 (4.8%) | 4 (9.5%) | 4.5 (0.5) | 40 (95.2%) | 4.0 (0.0) | 40 (95.2%) |
| ATLS | 29 (69.0%) | 4 (9.5%) | 5.0 (1.0) | 13 (31.0%) | 4.0 (2.0) | 14 (33.3%) |
| ATOM | 2 (4.8%) | 4 (9.5%) | 5.0 (0.0) | 40 (95.2%) | 5.0 (0.0) | 40 (95.2%) |
| BATLS | 4 (9.5%) | 4 (9.5%) | 4.0 (1.5) | 38 (90.5%) | 3.0 (N/A) | 39 (92.9%) |
| DSTC | 7 (16.7%) | 4 (9.5%) | 5.0 (0.0) | 35 (83.3%) | 4.0 (2.0) | 37 (88.1%) |
| DSTS | 2 (4.8%) | 4 (9.5%) | 4.0 (0.0) | 41 (97.6%) | 4.0 (0.0) | 41 (97.6%) |
| EMSB | 1 (2.4%) | 4 (9.5%) | 4.0 (0.0) | 41 (97.6%) | 2.0 (0.0) | 41 (97.6%) |
| EWSC | 2 (4.8%) | 4 (9.5%) | 5.0 (0.0) | 41 (97.6%) | 5.0 (0.0) | 41 (97.6%) |
| ETC | 4 (9.5%) | 4 (9.5%) | 5.0 (0.8) | 38 (90.5%) | 4.0 (3.0) | 38 (90.5%) |
| HELP | 3 (7.1%) | 4 (9.5%) | 5.0 (N/A) | 39 (92.9%) | 5.0 (N/A) | 39 (92.9%) |
| Onboarding | 10 (23.8%) | 4 (9.5%) | 5.0 (0.5) | 33 (78.6%) | 5.0 (2.0) | 33 (78.6%) |
| ICRC WSS | 23 (54.8%) | 4 (9.5%) | 5.0 (1.0) | 19 (45.2%) | 5.0 (3.0) | 21 (50.0%) |
| MIMMS | 1 (2.4%) | 4 (9.5%) | 3.0 (0.0) | 41 (97.6%) | 1.0 (0.0) | 41 (97.6%) |
| MOST | 3 (7.1%) | 4 (9.5%) | 5.0 (N/A) | 39 (92.9%) | 4.0 (N/A) | 40 (95.2%) |
| MRMI-course | 1 (2.4%) | 4 (9.5%) | 5.0 (0.0) | 41 (97.6%) | - | 42 (100%) |
| PHTLS | 3 (7.1%) | 4 (9.5%) | 5.0 (N/A) | 39 (92.9%) | 5.0 (N/A) | 39 (92.9%) |
| STAE | 6 (14.3%) | 4 (9.5%) | 5.0 (0.3) | 36 (85.7%) | 4.0 (3.3) | 36 (85.7%) |
| WALS | 1 (2.4%) | 4 (9.5%) | 3.0 (0.0) | 41 (97.6%) | 1.0 (0.0) | 41 (97.6%) |
| None | 2 (4.8%) | 2 (4.8%) | - | - | - | - |
| Other ^b^ | 9 (21.4%) | - | - | - | - | - |
| **ANESTHESIOLOGISTS (N=25)** | | | | | | |
| ABLS | 1 (4.0%) | 6 (24.0%) | 3.0 (0.0) | 24 (96.0%) | 4.0 (0.0) | 24 (96.0%) |
| ACLS | 7 (28.0%) | 6 (24.0%) | 5.0 (0.5) | 19 (76.0%) | 4.5 (2.5) | 19 (76.0%) |
| ALS | 6 (24.0%) | 6 (24.0%) | 5.0 (0.8) | 21 (84.0%) | 4.0 (N/A) | 22 (88.0%) |
| APLS | 4 (16.0%) | 6 (24.0%) | 4.5 (1.0) | 21 (84.0%) | 4.5 (1.0) | 21 (84.0%) |
| ATLS | 9 (36.0%) | 6 (24.0%) | 5.0 (2.5) | 16 (64.0%) | 5.0 (3.0) | 18 (72.0%) |
| ATACC | 2 (8.0%) | 6 (24.0%) | 4.0 (N/A) | 23 (92.0%) | 3.5 (N/A) | 23 (92.0%) |
| BATLS | 2 (8.0%) | 6 (24.0%) | 4.0 (0.0) | 23 (92.0%) | 3.5 (N/A) | 23 (92.0%) |
| DATC | 2 (8.0%) | 6 (24.0%) | 5.0 (0.0) | 23 (92.0%) | 4.5 (N/A) | 23 (92.0%) |
| DSTS | 1 (4.0%) | 6 (24.0%) | 5.0 (0.0) | 24 (96.0%) | 4.0 (0.0) | 24 (96.0%) |
| EPALS | 2 (8.0%) | 6 (24.0%) | 4.5 (N/A) | 23 (92.0%) | 4.5 (N/A) | 23 (92.0%) |
| ETC | 1 (4.0%) | 6 (24.0%) | 5.0 (0.0) | 24 (96.0%) | 4.0 (0.0) | 24 (96.0%) |
| HELP | 2 (8.0%) | 6 (24.0%) | 3.5 (N/A) | 23 (92.0%) | 1.0 (0.0) | 23(92.0%) |
| ICRC onboarding | 5 (20.0%) | 6 (24.0%) | 5.0 (1.0) | 20 (80.0%) | 3.0 (N/A) | 22 (88.0%) |
| ICRC WSS | 9 (36.0%) | 6 (24.0%) | 5.0 (1.8) | 17 (68.0%) | 3.5 (1.8) | 19 (76.0%) |
| ILS | 1 (4.0%) | 6 (24.0%) | 4.0 (0.0) | 24 (96.0%) | 4.0 (0.0) | 24 (96.0%) |
| MIMMS | 1 (4.0%) | 6 (24.0%) | 4.0 (0.0) | 24 (96.0%) | 4.0 (0.0) | 24 (96.0%) |
| MOST | 1 (4.0%) | 6 (24.0%) | 4.0 (0.0) | 24 (96.0%) | 4.0 (0.0) | 24 (96.0%) |
| MRMI-course | 1 (4.0%) | 6 (24.0%) | 4.0 (0.0) | 24 (96.0%) | 4.0 (0.0) | 24 (96.0%) |
| PHTLS | 1 (4.0%) | 6 (24.0%) | 5.0 (0.0) | 24 (96.0%) | 5.0 (0.0) | 24 (96.0%) |
| None | 0 (0.0%) | 6 (24.0%) | - | - | - | - |
| Other ^C^ | 3 (12.0%) | - | - | - | - | - |
| **NURSES (N=40)** | | | | | | |
| ACLS | 9 (22.5%) | 19 (47.5%) | 5.0 (1.5) | 31 (77.5%) | 5.0 (2.0) | 31 (77.5%) |
| ALS | 8 (20.0%) | 19 (47.5%) | 5.0 (0.8) | 32 (80.0%) | 3.5 (2.5) | 32 (80.0%) |
| ATLS | 6 (15.0%) | 19 (47.5%) | 5.0 (1.0) | 34 (85.0%) | 4.5 (1.3) | 34 (85.0%) |
| DSTC | 1 (2.5%) | 19 (47.5%) | 4.0 (0.0) | 39 (97.5%) | 2.0 (0.0) | 39 (97.5%) |
| DTN | 5 (12.5%) | 19 (47.5%) | 4.0 (1.5) | 35 (87.5%) | 4.0 (2.0) | 35 (87.5%) |
| ENPC | 3 (7.5%) | 19 (47.5%) | 5.0 (N/A) | 37 (92.5%) | 4.0 (N/A) | 37 (92.5%) |
| EPALS | 2 (5.0%) | 19 (47.5%) | 4.5 (N/A) | 38 (95.0%) | 5.0 (0.0) | 39 (97.5%) |
| ETC | 1 (2.5%) | 19 (47.5%) | 5.0 (0.0) | 39 (97.5%) | 3.0 (0.0) | 39 (97.5%) |
| HELP | 4 (10.0%) | 19 (47.5%) | 4.0 (2.0) | 36 (90.0%) | 4.0 (3.5) | 36 (90.0%) |
| Onboarding | 5 (12.5%) | 19 (47.5%) | 5.0 (0.8) | 36 (90.0%) | 4.5 (1.8) | 36 (90.0%) |
| ICRC WSS | 4 (10.0%) | 19 (47.5%) | 4.5 (1.8) | 36 (90.0%) | 3.5 (1.8) | 36 (90.0%) |
| ILS | 8 (20.0%) | 19 (47.5%) | 5.0 (0.8) | 32 (80.0%) | 4.5 (2.5) | 32 (80.0%) |
| PHTLS | 1 (2.5%) | 19 (47.5%) | 5.0 (0.0) | 39 (97.5%) | 5.0 (0.0) | 39 (97.5%) |
| TNCC | 4 (10.0%) | 19 (47.5%) | 5.0 (0.8) | 36 (90.0%) | 3.5 (1.8) | 36 (90.0%) |
| None | 2 (5.0%) | 17 (42.5%) | - | - | - | - |
| Other ^d^ | 5 (12.5%) | - | - | - | - | - |

*N/A Not applicable; IQR Interquartile range
^a^ Missing: surgeons 0 (valid 42); anaesthesiologists 0 (valid 25); nurses 0 (valid 40);
^b^ ‘Other’ courses: Damage Control Orthopaedic trauma Surgery (N=1); Humanitarian Surgery in austere environments (HSAE) (N=1); IMPACT (N=1); ERU course (N=1); International Surgical Workshop by MSF (N=3); WSES Mass Casualty Incident Management (N=1); Ultrasound in Emergency and Trauma (USET) (N=1)
^C^ ‘Other’ courses: FDM (N=1); FCCS (N=1); Primary trauma care (N=1)
^d^ ‘Other’ courses: ATK (N=1); Emergo Train system senior instructor course (N=1); ERTC (ICRC)
Master in Global Health (N=1); MSF Paediatric hospital care (N=1); Paediatric Advanced life support (N=1)*

*^$^ Abbreviation list:*

*ABLS Advanced Burn Life Support; ACLS Advanced Cardiovascular Life Support; ALS Advanced Life Support; APLS Advanced Paediatric Life Support; ASSET Advanced Surgical Skills for Exposure in Trauma; ATLS Advanced Trauma Life Support; ATOM Advanced Trauma Operative Management; ATACC Anaesthesia, Trauma and Critical Care; BATLS Battlefield Advanced Trauma Life Support; DATC Definitive Anaesthetic Trauma Care; DSTC Definitive Surgical Trauma Care; DSTS Definitive Surgical Trauma Skills; DTN Diploma Tropical Nursing; EMSB Emergency Management of the Severe Burns Course; ENPC Emergency Nursing Paediatric Course; EPALS European Paediatric Advanced Life Support; EWSC Emergency War Surgery Course; ETC European Trauma Course; HELP Health Emergencies in Large Populations; ICRC WSS International Committee of the Red Cross War Surgery Seminar; ILS Immediate Life Support; MIMMS Major Incident Medical Management and Support; MOST Military Operational Surgical Training; MRMI-course Medical Response to Major Incidents & Disasters; PHTLS Prehospital Trauma Life Support; STAE Surgical Training for Austere Environments; TNCC Trauma Nursing Core Course; WALS Wilderness Advanced Life Support;*

## Topics requested for additional training

Table 11. Topics requested for additional training and underlying reasons, stratified by profession

| **Topic** | **Requested**  **(N, %)^a^** | **Because respondent did not feel optimally prepared (N, %)** | | **Because respondent found topic interesting**  **(N, %)** | **Other reason**  **(N, %)** |
| --- | --- | --- | --- | --- | --- |
| **SURGEONS (N=42)** | | | | | |
| Amputation techniques | 3 (7.1%) | | 1 (2.4%) | 2 (4.8%) | 0 (0.0%) |
| Antibiotic management | 1 (2.4%) | | 0 (0.0%) | 1 (2.4%) | 0 (0.0%) |
| Burn treatment | 6 (14.3%) | | 2 (4.8%) | 4 (9.5%) | 0 (0.0%) |
| Fracture surgery | 12 (28.6%) | | 6 (14.3%) | 5 (11.9%) | 1 (2.4%) |
| Gastro-intestinal surgery | 0 (0.0%) | | 0 (0.0%) | 0 (0.0%) | 0 (0.0%) |
| Hand surgery | 8 (19.0%) | | 4 (9.5%) | 4 (9.5%) | 0 (0.0%) |
| ICU care | 6 (14.3%) | | 3 (7.1%) | 3 (7.1%) | 0 (0.0%) |
| Maxillofacial surgery | 20 (47.6%) | | 12 (28.6%) | 8 (19.0%) | 0 (0.0%) |
| Neurosurgery | 21 (50.0%) | | 16 (38.1%) | 5 (11.9%) | 0 (0.0%) |
| Nerve repair techniques | 9 (21.4%) | | 6 (14.3%) | 3 (7.1%) | 0 (0.0%) |
| Obstetrics/Gynaecology | 8 (19.0%) | | 5 (11.9%) | 2 (4.8%) | 1 (2.4%) |
| Ophthalmic surgery | 5 (11.9%) | | 5 (11.9%) | 0 (0.0%) | 0 (0.0%) |
| Paediatrics | 2 (4.8%) | | 2 (4.8%) | 0 (0.0%) | 0 (0.0%) |
| Paediatric surgery | 7 (16.7%) | | 4 (9.5%) | 3 (7.1%) | 0 (0.0%) |
| Plastic (reconstructive) surgery | 20 (47.6%) | | 13 (31.0%) | 7 (16.7%) | 0 (0.0%) |
| Resuscitation | 2 (4.8%) | | 0 (0.0%) | 2 (4.8%) | 0 (0.0%) |
| Sonography/ultrasound skills | 17 (40.5%) | | 11 (26.2%) | 5 (11.9%) | 1 (2.4%) |
| Soft tissue surgery | 0 (0.0%) | | 0 (0.0%) | 0 (0.0%) | 0 (0.0%) |
| (Surgical) decision making | 3 (7.1%) | | 0 (0.0%) | 3 (7.1%) | 0 (0.0%) |
| Triage skills | 4 (9.5%) | | 0 (0.0%) | 4 (9.5%) | 0 (0.0%) |
| Tropical diseases | 5 (11.9%) | | 5 (11.9%) |  | 0 (0.0%) |
| Thorax surgery | 12 (28.6%) | | 6 (14.3%) | 6 (14.3%) | 0 (0.0%) |
| Urology | 4 (9.5%) | | 1 (2.2%) | 3 (7.1%) | 0 (0.0%) |
| Vascular surgery | 16 (38.1%) | | 8 (19.0%) | 8 (19.0%) | 0 (0.0%) |
| Other | 0 (0.0%) | | 0 (0.0%) | 0 (0.0%) | 0 (0.0%) |
| No need/none^b^ | 1 (2.4%) | | 0 (0.0%) | 0 (0.0%) | 0 (0.0%) |
| **ANESTHESIOLOGISTS (N=25)** | | | | | |
| Amputation techniques | 1 (4.0%) | | 1 (4.0%) | 0 (0.0%) | 0 (0.0%) |
| Antibiotic management | 4 (16.0%) | | 1 (4.0%) | 3 (12.0%) | 0 (0.0%) |
| Burn treatment | 2 (8.0%) | | 0 (0.0%) | 2 (8.0%) | 0 (0.0%) |
| Fracture surgery | 0 (0.0%) | | - | - | - |
| Gastro-intestinal surgery | 0 (0.0%) | | - | - | - |
| Hand surgery | 0 (0.0%) | | - | - | - |
| ICU care | 4 (16.0%) | | 0 (0.0%) | 3 (12.0%) | 1 (4.0%) |
| Maxillofacial surgery | 0 (0.0%) | | - | - | - |
| Neurosurgery | 0 (0.0%) | | - | - | - |
| Nerve repair techniques | 0 (0.0%) | | - | - | - |
| Obstetrics/Gynaecology | 0 (0.0%) | | - | - | - |
| Ophthalmic surgery | 1 (4.0%) | | 1 (4.0%) | 0 (0.0%) | 0 (0.0%) |
| Paediatrics | 0 (0.0%) | | - | - | - |
| Paediatric surgery | 2 (8.0%) | | 1 (4.0%) | 1 (4.0%) | 0 (0.0%) |
| Plastic (reconstructive) surgery | 0 (0.0%) | | - | - | - |
| Resuscitation | 0 (0.0%) | | - | - | - |
| Sonography/ultrasound skills | 10 (40.0%) | | 1 (4.0%) | 7 (28.0%) | 2 (8.0%) |
| Soft tissue surgery | 0 (0.0%) | | - | - | - |
| (Surgical) decision making | 2 (8.0%) | | 1 (4.0%) | 1 (4.0%) | 0 (0.0%) |
| Triage skills | 5 (20.0%) | | 1 (4.0%) | 4 (16.0%) | 0 (0.0%) |
| Tropical diseases | 6 (24.0%) | | 5 (20.0%) | 1 (4.0%) | 0 (0.0%) |
| Thorax surgery | 0 (0.0%) | | - | - | - |
| Urology | 0 (0.0%) | | - | - | - |
| Vascular surgery | 0 (0.0%) | | - | - | - |
| Other  “Paediatric anaesthesia” | 1 (4.0%) | | 1 (4.0%) | 0 (0.0%) | 0 (0.0%) |
| No need/none^b^ | 1 (4.0%) | | - | - | - |
| **NURSES (N=40)** | | | | | |
| Care of patients with traction devices or external fixators | 6 (15.0%) | | 3 (7.5%) | 3 (7.5%) | 0 (0.0%) |
| Burns patients | 11 (27.5%) | | 6 (15.0%) | 4 (10.0%) | 1 (2.5%) |
| Obstetric patients | 7 (17.5%) | | 6 (15.0%) | 1 (2.5%) | 0 (0.0%) |
| Neurotrauma | 7 (17.5%) | | 3 (7.5%) | 3 (7.5%) | 1 (2.5%) |
| Paediatric patients | 8 (20.0%) | | 3 (7.5%) | 4 (10.0%) | 1 (2.5%) |
| ICU care | 8 (20.0%) | | 3 (7.5%) | 4 (10.0%) | 1 (2.5%) |
| New-born NCD management | 11 (27.5%) | | 5 (12.5%) | 4 (10.0%) | 2 (5.0%) |
| Pain management | 7 (17.5%) | | 0 (0.0%) | 5 (12.5%) | 2 (5.0%) |
| Tourniquet application | 3 (7.5%) | | 1 (2.5%) | 2 (5.0%) | 0 (0.0%) |
| Triage and mass casualty management | 14 (35.0%) | | 4 (10.0%) | 9 (22.5%) | 1 (2.5%) |
| Tropical disease management | 9 (22.5%) | | 5 (12.5%) | 2 (5.0%) | 2 (5.0%) |
| Wound care | 8 (20.0%) | | 1 (2.5%) | 5 (12.5%) | 2 (5.0%) |
| Other  Not specified | 1 (2.5%) | | 0 (0.0%) | 0 (0.0%) | 1 (2.5%) |
| No need/none^b^ | 1 (2.5%) | | - | - | - |

*^a^ Missing: surgeons 3 (7.1%); anaesthesiologists 7 (28.0%); nurses 18 (45.0%)
^b^ Missing ‘no need for training’: surgeons 2 (4.8%); anaesthesiologists 6 (24.0%); nurses 17 (42.5%)*

## Pre-deployment rating of self-perceived preparedness to treat paediatric and adult patients

Table 12. Pre-deployment self-perceived preparedness to treat paediatric patients

| **Level of preparedness** | **N (%)** |
| --- | --- |
| 1 Very unprepared | 0 (0.0%) |
| 2 | 6 (5.3%) |
| 3 | 20 (17.5%) |
| 4 | 33 (28.9%) |
| 5 More than sufficiently prepared | 28 (24.6%) |
| Missing | 27 (23.7%) |

Table 13. Pre-deployment self-perceived preparedness to treat adult patients

| **Level of preparedness** | **N (%)** |
| --- | --- |
| 1 Very unprepared | 0 (0.0%) |
| 2 | 2 (1.8%) |
| 3 | 3 (2.6%) |
| 4 | 28 (24.6%) |
| 5 More than sufficiently prepared | 54 (47.4%) |
| Missing | 27 (23.7%) |

## Rating of confidence in treating adult versus paediatric patients during deployment

Table 14. Level of confidence in treating adult versus paediatric patients

| **Level of confidence** | **N (%)** |
| --- | --- |
| Much more confident in treating adult patients | 9 (7.9%) |
| Slightly more confident in treating adult patients | 10 (8.8%) |
| Equally confident | 26 (22.8%) |
| Slightly more confident in treating paediatric patients | 2 (1.8%) |
| Much more confident in treating paediatric patients | 8 (7.0%) |
| Missing | 3 (2.6%) |

## Rating of pre-deployment preparation activities

Table 15. Rating of pre-deployment preparation activities

| **Pre-deployment preparation activity** | **N (%)^a^** | **Rank^b^** |
| --- | --- | --- |
| The basic/minimal education program for my current profession | 34 (29.8%) | Third most important |
| Training on Crew Resource Management (CRM) / team dynamics | 4 (3.5%) |  |
| Clinical placement in a trauma centre in an area with high rates of severe trauma injuries (expected to be somewhat similar as injuries seen on deployment | 35 (30.7%) |  |
| Military training courses on trauma care, emergency care or advanced life support | 14 (12.3%) |  |
| Other (civilian) training courses on trauma care, emergency care or advanced life support | 41 (36.0%) |  |
| Previous emergency care experience | 56 (49.1%) | First most important |
| Previous deployments | 47 (41.2%) | Second most important |
| ICRC onboarding mission | 30 (26.3%) |  |
| Information on the practical aspects of deployment | 25 (21.9%) |  |
| Getting familiar with the equipment you will have to your disposal during deployment | 18 (15.8%) |  |
| Other   - ATK - Management and Health Care in Tropical Countries - No course, daily business - None | 4 (3.5%) |  |
| None | 2 (1.8%) |  |
| Missing | 0 (0.0%) | 44 (38.6%) for Rank 1;  48 (42.1%) for Rank 2;  55 (48.2%) for Rank 3 |

## Rating of pre-deployment information about the mission

Table 16. Rating of pre-deployment information about the mission

| **Subject** | **Rating (median, IQR)** | **Missing (N, %)** |
| --- | --- | --- |
| Current situation in mission area | 4.0 (2.0) | 29 (25.4%) |
| Local environmental challenges | 4.0 (2.0) | 31 (27.2%) |
| Local living conditions | 4.0 (2.0) | 32 (28.1%) |
| Duties and responsibilities | 4.0 (1.0) | 28 (24.6%) |
| Field facilities and equipment available | 4.0 (2.0) | 31 (27.2%) |
| Means of contact with home/family during deployment | 5.0 (2.0) | 30 (26.3%) |
| Other ICRC participants of the mission and their responsibilities | 4.0 (2.0) | 29 (25.4%) |
| The nature and severity of local patients’ injuries | 4.0 (2.0) | 31 (27.2%) |

Scale 1-5 (1 Not at all adequately informed – 5 More than sufficiently informed)

## Post-deployment rating of self-perceived preparedness to treat paediatric and adult patients

Table 17. Post-deployment self-perceived preparedness to treat paediatric patients

| **Level of preparedness** | **N (%)** |
| --- | --- |
| 1 Very unprepared | 2 (3.4%) |
| 2 | 6 (10.3%) |
| 3 | 11 (19.0%) |
| 4 | 15 (25.9%) |
| 5 More than sufficiently prepared | 19 (32.8%) |
| Missing | 5 (8.6%) |

Table 18. Post-deployment self-perceived preparedness to treat adult patients

| **Level of preparedness** | **N (%)** |
| --- | --- |
| 1 Very unprepared | 1 (1.7%) |
| 2 | 3 (5.2%) |
| 3 | 8 (13.8%) |
| 4 | 8 (13.8%) |
| 5 More than sufficiently prepared | 34 (58.6%) |
| Missing | 4 (6.9%) |

## Time spent on call

Table 19. Time spent on call during deployment

|  | **N (%)** |
| --- | --- |
| Time spent on call per day  <8 hours  8-10 hours  10-12 hours  12-14 hours  >14 hours  Constant 24/7  I do not remember  Missing | 5 (8.6%)  8 (13.8%)  9 (15.5%)  3 (5.2%)  4 (6.9%)  22 (37.9%)  1 (1.7%)  3 (5.2%) |
| Days on call per week  <5 days  5 days  6 days  7 days  I do not remember  Missing | 9 (15.5%)  9 (15.5%)  6 (10.3%)  27 (46.6%)  1 (1.7%)  3 (5.2%) |

Table 20. Caseload during deployment

| **Caseload during deployment per week** | **N (%)** |
| --- | --- |
| None  <1  1-20  21-40  41-60  61-80  81-100  >100  I do not remember  Missing | 2 (3.4%)  0 (0.0%)  23 (39.7%)  15 (25.9%)  6 (10.3%)  2 (3.4%)  1 (1.7%)  0 (0.0%)  3 (5.2%)  4 (6.9%) |

## Injuries encountered during deployment

Table 21. Type of injuries encountered during deployment

| **Frequency of injuries encountered** | **N (%)** |
| --- | --- |
| Paediatric trauma  Less than once a month  Once a month  Few times a month  Once a week  Few times a week  Every day  I do not remember  Missing | 5 (8.6%)  4 (6.9%)  19 (32.8%)  6 (10.3%)  14 (24.1%)  3 (5.2%)  2 (3.4%)  3 (5.2%) |
| Adult trauma  Less than once a month  Once a month  Few times a month  Once a week  Few times a week  Every day  I do not remember  Missing | 0 (0.0%)  0 (0.0%)  7 (12.1%)  2 (3.4%)  9 (15.5%)  34 (58.6%)  0 (0.0%)  3 (5.2%) |
| Injuries outside field of specialization  Less than once a month  Once a month  Few times a month  Once a week  Few times a week  Every day  I do not remember  Missing | 10 (17.2%)  4 (6.9%)  13 (22.4%)  1 (1.7%)  10 (17.2%)  7 (12.1%)  7 (12.1%)  3 (5.2%) |

## Satisfaction with equipment during deployment

Table 22. Satisfaction with equipment during deployment

| **Treatment location** | **Equipment satisfaction for adult patients** | **Equipment satisfaction for paediatric patients** |
| --- | --- | --- |
| Prehospital (median, IQR)  Missing | 3.0 (2.3)  16 (29.1%) | 3.0 (3.0)  16 (29.1%) |
| Emergency Department (median, IQR)  Missing | 3.0 (1.0)  13 (23.6%) | 3.0 (2.0)  13 (23.6%) |
| Operation room (median, IQR)  Missing | 4.0 (2.0)  9 (16.4%) | 3.0 (1.8)  10 (18.2%) |
| Intensive Care Unit (median, IQR)  Missing | 3.0 (3.0)  15 (27.3%) | 3.0 (2.0)  18 (32.7%) |
| Follow-up (median, IQR)  Missing | 3.0 (2.0)  11 (20.0%) | 3.0 (2.0)  11 (20.0%) |

*Scale 1-5 (1 Very dissatisfied – 5 Very satisfied)*

## Availability of more experienced colleagues

Table 23. Availability of more experienced colleagues during deployment

| **Availability of experienced colleagues for consultation** | **N (%)** |
| --- | --- |
| Yes, on site | 32 (58.2%) |
| Yes, by phone | 20 (36.4%) |
| Yes, by internet | 27 (49.1%) |
| Yes, by other means | 3 (5.5%) |
| No, not possible at the right moments | 2 (3.6%) |
| No, never possible | 1 (1.8%) |
| I do not remember | 4 (7.3%) |
| Missing | 3 (5.5%) |

## Availability of referral centre

Table 24. Availability of referral centre during deployment

| **Availability of referral centre** | **N (%)** |
| --- | --- |
| Yes, for paediatric patients | 14 (25.5%) |
| Yes, for adult patients | 14 (25.5%) |
| No | 16 (29.1%) |
| I do not remember (N/A) | 23 (41.8%) |
| Missing | 3 (5.5%) |

Table 25. Time to reach referral centre during deployment

| **Time to reach referral centre** | **Adult patients (N, %)** | **Paediatric patients (N, %)** |
| --- | --- | --- |
| <1 hour | 8 (14.5%) | 8 (14.5%) |
| >1 hour | 3 (5.5%) | 2 (3.6%) |
| >2 hours | 15 (27.3%) | 14 (25.5%) |
| I do not remember | 8 (14.5%) | 10 (18.2%) |
| Missing | 24 (43.6%) | 24 (43.6%) |

## Structured methods of communication during deployment

Table 26. Structured methods of communication during handover of patient information during deployment

| **Communication method** | **Applied (N, %)** |
| --- | --- |
| MIST | 17 (30.9%) |
| SBAR | 12 (21.8%) |
| RSVP | 8 (14.5%) |
| None | 23 (41.8%) |
| There was no communication with other services | 7 (12.7%) |
| Other | 2 (3.6%) |
| I do not remember | 4 (7.3%) |
| Missing | 3 (5.5%) |

## Deployment impact on trauma management skills

Table 27. Deployment impact on trauma management skills

|  | **N (%)** |
| --- | --- |
| Much deteriorated | 0 |
| Slightly deteriorated | 0 |
| Did not change | 18 |
| Slightly improved | 15 |
| Much improved | 22 |
| Missing | 3 |

Median 4.0 (IQR 2.0)

## Deployment impact on skills in primary specialism

Table 28. Deployment impact on skills in primary specialism

|  | **N (%)** |
| --- | --- |
| Much deteriorated | 1 |
| Slightly deteriorated | 1 |
| Did not change | 22 |
| Slightly improved | 9 |
| Much improved | 17 |
| Missing | 8 |

Median 4.0 (IQR 2.0)

## Deployment impact on personal development

Table 29. Deployment impact on personal development

|  | **N (%)** |
| --- | --- |
| Major negative | 1 |
| Minor negative | 3 |
| Neutral | 9 |
| Minor positive | 8 |
| Major positive | 33 |
| Missing | 4 |

Median 5.0 (IQR 1.3)

## Deployment impact on private situation

Table 30. Deployment impact on private situation

|  | **N (%)** |
| --- | --- |
| Major negative | 2 |
| Minor negative | 5 |
| Neutral | 25 |
| Minor positive | 10 |
| Major positive | 11 |
| Missing | 5 |

Median 3.0 (IQR 1.0)

## Need for peer-to-peer contact during deployment

Table 31. Need for peer-to-peer contact

|  | **N (%)** |
| --- | --- |
| Not at all | 7 |
| Very little | 4 |
| Undecided | 9 |
| Somewhat | 20 |
| Very much | 14 |
| Missing | 4 |

Median 4.0 IQR (2.0)

## Need for debriefing during deployment

Table 32. Need for debriefing

|  | **N (%)** |
| --- | --- |
| Not at all | 4 |
| Very little | 1 |
| Undecided | 6 |
| Somewhat | 22 |
| Very much | 22 |
| Missing | 3 |

Median 4.0 (IQR 1.0)

## Need for professional psychological help during deployment

Table 33. Need for professional psychological help

|  | **N (%)** |
| --- | --- |
| Not at all | 28 |
| Very little | 9 |
| Undecided | 8 |
| Somewhat | 5 |
| Very much | 3 |
| Missing | 5 |

Median 1.0 (IQR 2.0)

## Actual peer-to-peer contact, debriefing, and professional psychological help received

Table 34. Whether actual peer-to-peer contact, debriefing or psychological help was provided during deployment

|  | **Physician**  **(N=32)** | **Nurses**  **(N=25)** | **Total**  **(N=58)** |
| --- | --- | --- | --- |
| Actual peer-to-peer contact | 20 (62.5%) | 17 (68.0%) | 38 (65.5%) |
| Actual debriefing | 26 (81.3%) | 19 (76.0%) | 45 (77.6%) |
| Actual professional psychological help | 1 (3.1%) | 0 (0.0%) | 1 (1.7%) |
| Missing | 2 (6.3%) | 1 (4.0%) | 3 (5.2%) |

## Activities helpful to deal with stress during deployment

Table 35. Activities helpful to deal with stress during deployment

| **Activity** | **N (%)** |
| --- | --- |
| Stress management training beforehand | 17 (29.3%) |
| A card/kit with stress management advice for members to carry | 7 (12.1%) |
| Availability of a mental health professional during deployment | 30 (51.7%) |
| Briefing about strategies relevant to deployment before departure | 28 (48.3%) |
| Follow-up of individual ICRC team members | 29 (50.0%) |
| Online social support network | 18 (31.0%) |
| Other | 5 (8.6%) |
| Missing | 3 (5.2%) |

# COMPARATIVE STATISTICS

## **Grouping variable: profession (physician/nurse)**

### Weeks of deployment with ICRC

| **Descriptive Statistics** | | | | | | | | |
| --- | --- | --- | --- | --- | --- | --- | --- | --- |
|  | N | Mean | Std. Deviation | Minimum | Maximum | Percentiles | | |
|  |  |  |  |  |  | 25th | 50th (Median) | 75th |
| In total, how many weeks did you spend in the field with this organization? | 81 | 101,91 | 146,283 | 0 | 720 | 21,00 | 40,00 | 128,00 |
| 1=Doctor; 2=Nurse | 112 | 1,36 | ,481 | 1 | 2 | 1,00 | 1,00 | 2,00 |

| **Ranks** | | | | |
| --- | --- | --- | --- | --- |
|  | 1=Doctor; 2=Nurse | N | Mean Rank | Sum of Ranks |
| In total, how many weeks did you spend in the field with this organization? | Doctor | 53 | 40,02 | 2121,00 |
|  | Nurse | 28 | 42,86 | 1200,00 |
|  | Total | 81 |  |  |

| **Test Statistics^a^** | |
| --- | --- |
|  | In total, how many weeks did you spend in the field with this organization? |
| Mann-Whitney U | 690,000 |
| Wilcoxon W | 2121,000 |
| Z | -,517 |
| Asymp. Sig. (2-tailed) | ,605 |
| a. Grouping Variable: 1=Doctor; 2=Nurse | |

### Years of clinical experience

| **Descriptive Statistics** | | | | | | | | |
| --- | --- | --- | --- | --- | --- | --- | --- | --- |
|  | N | Mean | Std. Deviation | Minimum | Maximum | Percentiles | | |
|  |  |  |  |  |  | 25th | 50th (Median) | 75th |
| Years of clinical experience in your field of expertise since your official registration in your specialty: | 108 | 16,86 | 9,499 | 3 | 39 | 9,00 | 15,00 | 22,75 |
| 1=Doctor; 2=Nurse | 112 | 1,36 | ,481 | 1 | 2 | 1,00 | 1,00 | 2,00 |

| **Ranks** | | | | |
| --- | --- | --- | --- | --- |
|  | 1=Doctor; 2=Nurse | N | Mean Rank | Sum of Ranks |
| Years of clinical experience in your field of expertise since your official registration in your specialty: | Doctor | 69 | 56,07 | 3869,00 |
|  | Nurse | 39 | 51,72 | 2017,00 |
|  | Total | 108 |  |  |

| **Test Statistics^a^** | |
| --- | --- |
|  | Years of clinical experience in your field of expertise since your official registration in your specialty: |
| Mann-Whitney U | 1237,000 |
| Wilcoxon W | 2017,000 |
| Z | -,695 |
| Asymp. Sig. (2-tailed) | ,487 |
| a. Grouping Variable: 1=Doctor; 2=Nurse | |

### Pre-deployment rating of self-perceived preparedness to treat paediatric patients*

| **Descriptive Statistics** | | | | | | | | |
| --- | --- | --- | --- | --- | --- | --- | --- | --- |
|  | N | Mean | Std. Deviation | Minimum | Maximum | Percentiles | | |
|  |  |  |  |  |  | 25th | 50th (Median) | 75th |
| Do you feel professionally prepared to treat/care for paediatric trauma patients during your upcoming deployment? [I feel...] | 87 | 3,95 | ,914 | 2 | 5 | 3,00 | 4,00 | 5,00 |
| 1=Doctor; 2=Nurse | 112 | 1,36 | ,481 | 1 | 2 | 1,00 | 1,00 | 2,00 |

| **Ranks** | | | | |
| --- | --- | --- | --- | --- |
|  | 1=Doctor; 2=Nurse | N | Mean Rank | Sum of Ranks |
| Do you feel professionally prepared to treat/care for paediatric trauma patients during your upcoming deployment? [I feel...] | Doctor | 61 | 47,78 | 2914,50 |
|  | Nurse | 26 | 35,13 | 913,50 |
|  | Total | 87 |  |  |

| **Test Statistics^a^** | |
| --- | --- |
|  | Do you feel professionally prepared to treat/care for paediatric trauma patients during your upcoming deployment? [I feel...] |
| Mann-Whitney U | 562,500 |
| Wilcoxon W | 913,500 |
| Z | -2,253 |
| Asymp. Sig. (2-tailed) | ,024 |
| a. Grouping Variable: 1=Doctor; 2=Nurse | |

### Pre-deployment rating of self-perceived preparedness to treat adult patients*

| **Descriptive Statistics** | | | | | | | | |
| --- | --- | --- | --- | --- | --- | --- | --- | --- |
|  | N | Mean | Std. Deviation | Minimum | Maximum | Percentiles | | |
|  |  |  |  |  |  | 25th | 50th (Median) | 75th |
| Do you feel professionally prepared to treat/care for adult trauma patients during your upcoming deployment? [I feel ...] | 87 | 4,54 | ,679 | 2 | 5 | 4,00 | 5,00 | 5,00 |
| 1=Doctor; 2=Nurse | 112 | 1,36 | ,481 | 1 | 2 | 1,00 | 1,00 | 2,00 |

| **Ranks** | | | | |
| --- | --- | --- | --- | --- |
|  | 1=Doctor; 2=Nurse | N | Mean Rank | Sum of Ranks |
| Do you feel professionally prepared to treat/care for adult trauma patients during your upcoming deployment? [I feel ...] | Doctor | 61 | 47,43 | 2893,50 |
|  | Nurse | 26 | 35,94 | 934,50 |
|  | Total | 87 |  |  |

| **Test Statistics^a^** | |
| --- | --- |
|  | Do you feel professionally prepared to treat/care for adult trauma patients during your upcoming deployment? [I feel ...] |
| Mann-Whitney U | 583,500 |
| Wilcoxon W | 934,500 |
| Z | -2,277 |
| Asymp. Sig. (2-tailed) | ,023 |
| a. Grouping Variable: 1=Doctor; 2=Nurse | |

### Number of topics requested for additional training

| **Descriptive Statistics** | | | | | | | | |
| --- | --- | --- | --- | --- | --- | --- | --- | --- |
|  | N | Mean | Std. Deviation | Minimum | Maximum | Percentiles | | |
|  |  |  |  |  |  | 25th | 50th (Median) | 75th |
| Number of topics requested for additional training | 114 | 3,1930 | 3,36004 | ,00 | 18,00 | ,0000 | 3,0000 | 6,0000 |
| 1=Doctor; 2=Nurse | 112 | 1,36 | ,481 | 1 | 2 | 1,00 | 1,00 | 2,00 |

| **Ranks** | | | | |
| --- | --- | --- | --- | --- |
|  | 1=Doctor; 2=Nurse | N | Mean Rank | Sum of Ranks |
| Number of topics requested for additional training | Doctor | 72 | 58,88 | 4239,50 |
|  | Nurse | 40 | 52,21 | 2088,50 |
|  | Total | 112 |  |  |

| **Test Statistics^a^** | |
| --- | --- |
|  | Number of topics requested for additional training |
| Mann-Whitney U | 1268,500 |
| Wilcoxon W | 2088,500 |
| Z | -1,056 |
| Asymp. Sig. (2-tailed) | ,291 |
| a. Grouping Variable: 1=Doctor; 2=Nurse | |

### Rating of pre-deployment training, knowledge and skills regarding injuries treated during deployment*

| **Descriptive Statistics** | | | | | | | | |
| --- | --- | --- | --- | --- | --- | --- | --- | --- |
|  | N | Mean | Std. Deviation | Minimum | Maximum | Percentiles | | |
|  |  |  |  |  |  | 25th | 50th (Median) | 75th |
| Were your pre-deployment training, knowledge and skills sufficient regarding the injuries you have treated during your last ICRC deployment? [My training, knowledge and skills were...] | 54 | 4,06 | 1,156 | 1 | 5 | 3,00 | 4,50 | 5,00 |
| 1=Doctor; 2=Nurse | 57 | 1,44 | ,501 | 1 | 2 | 1,00 | 1,00 | 2,00 |

| **Ranks** | | | | |
| --- | --- | --- | --- | --- |
|  | 1=Doctor; 2=Nurse | N | Mean Rank | Sum of Ranks |
| Were your pre-deployment training, knowledge and skills sufficient regarding the injuries you have treated during your last ICRC deployment? [My training, knowledge and skills were...] | Doctor | 30 | 32,03 | 961,00 |
|  | Nurse | 23 | 20,43 | 470,00 |
|  | Total | 53 |  |  |

| **Test Statistics^a^** | |
| --- | --- |
|  | Were your pre-deployment training, knowledge and skills sufficient regarding the injuries you have treated during your last ICRC deployment? [My training, knowledge and skills were...] |
| Mann-Whitney U | 194,000 |
| Wilcoxon W | 470,000 |
| Z | -2,933 |
| Asymp. Sig. (2-tailed) | ,003 |
| a. Grouping Variable: 1=Doctor; 2=Nurse | |

### Rating of pre-deployment training, knowledge and skills of colleagues*

| **Descriptive Statistics** | | | | | | | | |
| --- | --- | --- | --- | --- | --- | --- | --- | --- |
|  | N | Mean | Std. Deviation | Minimum | Maximum | Percentiles | | |
|  |  |  |  |  |  | 25th | 50th (Median) | 75th |
| In general, how do you consider the medical training, knowledge and skills of your direct colleagues during your last ICRC deployment? [My collegues training, knowledge and skills were...] | 54 | 4,13 | 1,100 | 1 | 5 | 3,00 | 5,00 | 5,00 |
| 1=Doctor; 2=Nurse | 57 | 1,44 | ,501 | 1 | 2 | 1,00 | 1,00 | 2,00 |

| **Ranks** | | | | |
| --- | --- | --- | --- | --- |
|  | 1=Doctor; 2=Nurse | N | Mean Rank | Sum of Ranks |
| In general, how do you consider the medical training, knowledge and skills of your direct colleagues during your last ICRC deployment? [My collegues training, knowledge and skills were...] | Doctor | 30 | 30,45 | 913,50 |
|  | Nurse | 23 | 22,50 | 517,50 |
|  | Total | 53 |  |  |

| **Test Statistics^a^** | |
| --- | --- |
|  | In general, how do you consider the medical training, knowledge and skills of your direct colleagues during your last ICRC deployment? [My collegues training, knowledge and skills were...] |
| Mann-Whitney U | 241,500 |
| Wilcoxon W | 517,500 |
| Z | -2,048 |
| Asymp. Sig. (2-tailed) | ,041 |
| a. Grouping Variable: 1=Doctor; 2=Nurse | |

### Rating of equipment to treat adult patients during deployment*

| **Descriptive Statistics** | | | | | | | | |
| --- | --- | --- | --- | --- | --- | --- | --- | --- |
|  | N | Mean | Std. Deviation | Minimum | Maximum | Percentiles | | |
|  |  |  |  |  |  | 25th | 50th (Median) | 75th |
| In the following locations, what did you think about the equipment you had to your disposal to treat/care for adult patients during your last ICRC deployment? [Prehospital] | 42 | 3,14 | 1,372 | 1 | 5 | 2,00 | 3,00 | 4,25 |
| In the following locations, what did you think about the equipment you had to your disposal to treat/care for adult patients during your last ICRC deployment? [ER] | 45 | 3,31 | 1,184 | 1 | 5 | 3,00 | 3,00 | 4,00 |
| In the following locations, what did you think about the equipment you had to your disposal to treat/care for adult patients during your last ICRC deployment? [OR] | 49 | 4,08 | 1,038 | 2 | 5 | 3,00 | 4,00 | 5,00 |
| In the following locations, what did you think about the equipment you had to your disposal to treat/care for adult patients during your last ICRC deployment? [ICU] | 43 | 3,21 | 1,440 | 1 | 5 | 2,00 | 3,00 | 5,00 |
| In the following locations, what did you think about the equipment you had to your disposal to treat/care for adult patients during your last ICRC deployment? [Follow-up] | 47 | 3,55 | 1,176 | 1 | 5 | 3,00 | 3,00 | 5,00 |
| 1=Doctor; 2=Nurse | 57 | 1,44 | ,501 | 1 | 2 | 1,00 | 1,00 | 2,00 |

| **Ranks** | | | | |
| --- | --- | --- | --- | --- |
|  | 1=Doctor; 2=Nurse | N | Mean Rank | Sum of Ranks |
| In the following locations, what did you think about the equipment you had to your disposal to treat/care for adult patients during your last ICRC deployment? [Prehospital] | Doctor | 22 | 22,11 | 486,50 |
|  | Nurse | 19 | 19,71 | 374,50 |
|  | Total | 41 |  |  |
| In the following locations, what did you think about the equipment you had to your disposal to treat/care for adult patients during your last ICRC deployment? [ER] | Doctor | 26 | 24,58 | 639,00 |
|  | Nurse | 18 | 19,50 | 351,00 |
|  | Total | 44 |  |  |
| In the following locations, what did you think about the equipment you had to your disposal to treat/care for adult patients during your last ICRC deployment? [OR] | Doctor | 28 | 27,96 | 783,00 |
|  | Nurse | 20 | 19,65 | 393,00 |
|  | Total | 48 |  |  |
| In the following locations, what did you think about the equipment you had to your disposal to treat/care for adult patients during your last ICRC deployment? [ICU] | Doctor | 24 | 20,42 | 490,00 |
|  | Nurse | 18 | 22,94 | 413,00 |
|  | Total | 42 |  |  |
| In the following locations, what did you think about the equipment you had to your disposal to treat/care for adult patients during your last ICRC deployment? [Follow-up] | Doctor | 27 | 26,85 | 725,00 |
|  | Nurse | 19 | 18,74 | 356,00 |
|  | Total | 46 |  |  |

| **Test Statistics^a^** | | | | | |
| --- | --- | --- | --- | --- | --- |
|  | In the following locations, what did you think about the equipment you had to your disposal to treat/care for adult patients during your last ICRC deployment? [Prehospital] | In the following locations, what did you think about the equipment you had to your disposal to treat/care for adult patients during your last ICRC deployment? [ER] | In the following locations, what did you think about the equipment you had to your disposal to treat/care for adult patients during your last ICRC deployment? [OR] | In the following locations, what did you think about the equipment you had to your disposal to treat/care for adult patients during your last ICRC deployment? [ICU] | In the following locations, what did you think about the equipment you had to your disposal to treat/care for adult patients during your last ICRC deployment? [Follow-up] |
| Mann-Whitney U | 184,500 | 180,000 | 183,000 | 190,000 | 166,000 |
| Wilcoxon W | 374,500 | 351,000 | 393,000 | 490,000 | 356,000 |
| Z | -,661 | -1,366 | -2,192 | -,682 | -2,137 |
| Asymp. Sig. (2-tailed) | ,508 | ,172 | ,028 | ,495 | ,033 |
| a. Grouping Variable: 1=Doctor; 2=Nurse | | | | | |

### Rating of equipment to treat paediatric patients during deployment

| **Descriptive Statistics** | | | | | | | | |
| --- | --- | --- | --- | --- | --- | --- | --- | --- |
|  | N | Mean | Std. Deviation | Minimum | Maximum | Percentiles | | |
|  |  |  |  |  |  | 25th | 50th (Median) | 75th |
| In the following locations, what did you think about the equipment you had to your disposal to treat/care for paediatric patients during your last ICRC deployment? [Prehospital] | 42 | 2,64 | 1,394 | 1 | 5 | 1,00 | 3,00 | 4,00 |
| In the following locations, what did you think about the equipment you had to your disposal to treat/care for paediatric patients during your last ICRC deployment? [ER] | 45 | 3,00 | 1,168 | 1 | 5 | 2,00 | 3,00 | 4,00 |
| In the following locations, what did you think about the equipment you had to your disposal to treat/care for paediatric patients during your last ICRC deployment? [OR] | 48 | 3,46 | 1,184 | 1 | 5 | 3,00 | 3,00 | 4,75 |
| In the following locations, what did you think about the equipment you had to your disposal to treat/care for paediatric patients during your last ICRC deployment? [ICU] | 40 | 2,90 | 1,355 | 1 | 5 | 2,00 | 3,00 | 4,00 |
| In the following locations, what did you think about the equipment you had to your disposal to treat/care for paediatric patients during your last ICRC deployment? [Follow-up] | 47 | 3,40 | 1,210 | 1 | 5 | 3,00 | 3,00 | 5,00 |
| 1=Doctor; 2=Nurse | 57 | 1,44 | ,501 | 1 | 2 | 1,00 | 1,00 | 2,00 |

| **Ranks** | | | | |
| --- | --- | --- | --- | --- |
|  | 1=Doctor; 2=Nurse | N | Mean Rank | Sum of Ranks |
| In the following locations, what did you think about the equipment you had to your disposal to treat/care for paediatric patients during your last ICRC deployment? [Prehospital] | Doctor | 23 | 22,24 | 511,50 |
|  | Nurse | 19 | 20,61 | 391,50 |
|  | Total | 42 |  |  |
| In the following locations, what did you think about the equipment you had to your disposal to treat/care for paediatric patients during your last ICRC deployment? [ER] | Doctor | 24 | 24,08 | 578,00 |
|  | Nurse | 20 | 20,60 | 412,00 |
|  | Total | 44 |  |  |
| In the following locations, what did you think about the equipment you had to your disposal to treat/care for paediatric patients during your last ICRC deployment? [OR] | Doctor | 27 | 26,67 | 720,00 |
|  | Nurse | 20 | 20,40 | 408,00 |
|  | Total | 47 |  |  |
| In the following locations, what did you think about the equipment you had to your disposal to treat/care for paediatric patients during your last ICRC deployment? [ICU] | Doctor | 22 | 19,80 | 435,50 |
|  | Nurse | 18 | 21,36 | 384,50 |
|  | Total | 40 |  |  |
| In the following locations, what did you think about the equipment you had to your disposal to treat/care for paediatric patients during your last ICRC deployment? [Follow-up] | Doctor | 26 | 26,37 | 685,50 |
|  | Nurse | 20 | 19,78 | 395,50 |
|  | Total | 46 |  |  |

| **Test Statistics^a^** | | | | | |
| --- | --- | --- | --- | --- | --- |
|  | In the following locations, what did you think about the equipment you had to your disposal to treat/care for paediatric patients during your last ICRC deployment? [Prehospital] | In the following locations, what did you think about the equipment you had to your disposal to treat/care for paediatric patients during your last ICRC deployment? [ER] | In the following locations, what did you think about the equipment you had to your disposal to treat/care for paediatric patients during your last ICRC deployment? [OR] | In the following locations, what did you think about the equipment you had to your disposal to treat/care for paediatric patients during your last ICRC deployment? [ICU] | In the following locations, what did you think about the equipment you had to your disposal to treat/care for paediatric patients during your last ICRC deployment? [Follow-up] |
| Mann-Whitney U | 201,500 | 202,000 | 198,000 | 182,500 | 185,500 |
| Wilcoxon W | 391,500 | 412,000 | 408,000 | 435,500 | 395,500 |
| Z | -,441 | -,946 | -1,604 | -,434 | -1,733 |
| Asymp. Sig. (2-tailed) | ,659 | ,344 | ,109 | ,665 | ,083 |
| Exact Sig. [2*(1-tailed Sig.)] |  |  |  | ,677^b^ |  |
| a. Grouping Variable: 1=Doctor; 2=Nurse; b. Not corrected for ties. | | | | | |
| Post-deployment rating of self-perceived preparedness to treat paediatric patients*  \| **Descriptive Statistics** \| \| \| \| \| \| \| \| \| \| --- \| --- \| --- \| --- \| --- \| --- \| --- \| --- \| --- \| \|  \| N \| Mean \| Std. Deviation \| Minimum \| Maximum \| Percentiles \| \| \| \| 25th \| 50th (Median) \| 75th \| \| In hindsight, how would you rate your preparedness prior to deployment to treat/care for paediatric trauma patients? [I was...] \| 53 \| 3,81 \| 1,161 \| 1 \| 5 \| 3,00 \| 4,00 \| 5,00 \| \| 1=Doctor; 2=Nurse \| 57 \| 1,44 \| ,501 \| 1 \| 2 \| 1,00 \| 1,00 \| 2,00 \| | | | | | |

| **Ranks** | | | | |
| --- | --- | --- | --- | --- |
|  | 1=Doctor; 2=Nurse | N | Mean Rank | Sum of Ranks |
| In hindsight, how would you rate your preparedness prior to deployment to treat/care for paediatric trauma patients? [I was...] | Doctor | 30 | 31,47 | 944,00 |
|  | Nurse | 22 | 19,73 | 434,00 |
|  | Total | 52 |  |  |

| **Test Statistics^a^** | |
| --- | --- |
|  | In hindsight, how would you rate your preparedness prior to deployment to treat/care for paediatric trauma patients? [I was...] |
| Mann-Whitney U | 181,000 |
| Wilcoxon W | 434,000 |
| Z | -2,879 |
| Asymp. Sig. (2-tailed) | ,004 |
| a. Grouping Variable: 1=Doctor; 2=Nurse | |

### Post-deployment rating of self-perceived preparedness to treat adult patients*

| **Descriptive Statistics** | | | | | | | | |
| --- | --- | --- | --- | --- | --- | --- | --- | --- |
|  | N | Mean | Std. Deviation | Minimum | Maximum | Percentiles | | |
|  |  |  |  |  |  | 25th | 50th (Median) | 75th |
| In hindsight, how would you rate your preparedness prior to deployment to treat/care for adult trauma patients? [I was...] | 54 | 4,31 | 1,043 | 1 | 5 | 4,00 | 5,00 | 5,00 |
| 1=Doctor; 2=Nurse | 57 | 1,44 | ,501 | 1 | 2 | 1,00 | 1,00 | 2,00 |

| **Ranks** | | | | |
| --- | --- | --- | --- | --- |
|  | 1=Doctor; 2=Nurse | N | Mean Rank | Sum of Ranks |
| In hindsight, how would you rate your preparedness prior to deployment to treat/care for adult trauma patients? [I was...] | Doctor | 29 | 32,16 | 932,50 |
|  | Nurse | 24 | 20,77 | 498,50 |
|  | Total | 53 |  |  |

| **Test Statistics^a^** | |
| --- | --- |
|  | In hindsight, how would you rate your preparedness prior to deployment to treat/care for adult trauma patients? [I was...] |
| Mann-Whitney U | 198,500 |
| Wilcoxon W | 498,500 |
| Z | -3,126 |
| Asymp. Sig. (2-tailed) | ,002 |
| a. Grouping Variable: 1=Doctor; 2=Nurse | |

### Need for peer-to-peer contact

| **Descriptive Statistics** | | | | | | | | |
| --- | --- | --- | --- | --- | --- | --- | --- | --- |
|  | N | Mean | Std. Deviation | Minimum | Maximum | Percentiles | | |
|  |  |  |  |  |  | 25th | 50th (Median) | 75th |
| Did you feel the need for a consistent contact person (peer-to-peer / colleague) on site, to talk about your experiences during your last ICRC deployment? [I felt this need...] | 54 | 3,56 | 1,313 | 1 | 5 | 3,00 | 4,00 | 5,00 |
| 1=Doctor; 2=Nurse | 57 | 1,44 | ,501 | 1 | 2 | 1,00 | 1,00 | 2,00 |

| **Ranks** | | | | |
| --- | --- | --- | --- | --- |
|  | 1=Doctor; 2=Nurse | N | Mean Rank | Sum of Ranks |
| Did you feel the need for a consistent contact person (peer-to-peer / colleague) on site, to talk about your experiences during your last ICRC deployment? [I felt this need...] | Doctor | 30 | 28,17 | 845,00 |
|  | Nurse | 24 | 26,67 | 640,00 |
|  | Total | 54 |  |  |

| **Test Statistics^a^** | |
| --- | --- |
|  | Did you feel the need for a consistent contact person (peer-to-peer / colleague) on site, to talk about your experiences during your last ICRC deployment? [I felt this need...] |
| Mann-Whitney U | 340,000 |
| Wilcoxon W | 640,000 |
| Z | -,362 |
| Asymp. Sig. (2-tailed) | ,717 |
| a. Grouping Variable: 1=Doctor; 2=Nurse | |

### Need for debriefing

| **Descriptive Statistics** | | | | | | | | |
| --- | --- | --- | --- | --- | --- | --- | --- | --- |
|  | N | Mean | Std. Deviation | Minimum | Maximum | Percentiles | | |
|  |  |  |  |  |  | 25th | 50th (Median) | 75th |
| Did you ever feel the need to debrief (in any form) within the team on your experiences during your last ICRC deployment? [I felt this need...] | 55 | 4,04 | 1,122 | 1 | 5 | 4,00 | 4,00 | 5,00 |
| 1=Doctor; 2=Nurse | 57 | 1,44 | ,501 | 1 | 2 | 1,00 | 1,00 | 2,00 |

| **Ranks** | | | | |
| --- | --- | --- | --- | --- |
|  | 1=Doctor; 2=Nurse | N | Mean Rank | Sum of Ranks |
| Did you ever feel the need to debrief (in any form) within the team on your experiences during your last ICRC deployment? [I felt this need...] | Doctor | 30 | 25,98 | 779,50 |
|  | Nurse | 24 | 29,40 | 705,50 |
|  | Total | 54 |  |  |

| **Test Statistics^a^** | |
| --- | --- |
|  | Did you ever feel the need to debrief (in any form) within the team on your experiences during your last ICRC deployment? [I felt this need...] |
| Mann-Whitney U | 314,500 |
| Wilcoxon W | 779,500 |
| Z | -,848 |
| Asymp. Sig. (2-tailed) | ,396 |
| a. Grouping Variable: 1=Doctor; 2=Nurse | |

### Need for professional psychological help

| **Descriptive Statistics** | | | | | | | | |
| --- | --- | --- | --- | --- | --- | --- | --- | --- |
|  | N | Mean | Std. Deviation | Minimum | Maximum | Percentiles | | |
|  |  |  |  |  |  | 25th | 50th (Median) | 75th |
| Did you ever feel the need for professional psychological help during your last ICRC deployment? [I felt this need...] | 53 | 1,98 | 1,263 | 1 | 5 | 1,00 | 1,00 | 3,00 |
| 1=Doctor; 2=Nurse | 57 | 1,44 | ,501 | 1 | 2 | 1,00 | 1,00 | 2,00 |

| **Ranks** | | | | |
| --- | --- | --- | --- | --- |
|  | 1=Doctor; 2=Nurse | N | Mean Rank | Sum of Ranks |
| Did you ever feel the need for professional psychological help during your last ICRC deployment? [I felt this need...] | Doctor | 29 | 24,76 | 718,00 |
|  | Nurse | 23 | 28,70 | 660,00 |
|  | Total | 52 |  |  |

| **Test Statistics^a^** | |
| --- | --- |
|  | Did you ever feel the need for professional psychological help during your last ICRC deployment? [I felt this need...] |
| Mann-Whitney U | 283,000 |
| Wilcoxon W | 718,000 |
| Z | -1,018 |
| Asymp. Sig. (2-tailed) | ,309 |
| a. Grouping Variable: 1=Doctor; 2=Nurse | |

### Impact of deployment on personal development

| **Descriptive Statistics** | | | | | | | | |
| --- | --- | --- | --- | --- | --- | --- | --- | --- |
|  | N | Mean | Std. Deviation | Minimum | Maximum | Percentiles | | |
|  |  |  |  |  |  | 25th | 50th (Median) | 75th |
| What effect did your last ICRC deployment have on your personal development? [My last ICRC deployment had a...] | 54 | 4,28 | 1,054 | 1 | 5 | 3,75 | 5,00 | 5,00 |
| 1=Doctor; 2=Nurse | 57 | 1,44 | ,501 | 1 | 2 | 1,00 | 1,00 | 2,00 |

| **Ranks** | | | | |
| --- | --- | --- | --- | --- |
|  | 1=Doctor; 2=Nurse | N | Mean Rank | Sum of Ranks |
| What effect did your last ICRC deployment have on your personal development? [My last ICRC deployment had a...] | Doctor | 30 | 28,55 | 856,50 |
|  | Nurse | 23 | 24,98 | 574,50 |
|  | Total | 53 |  |  |

| **Test Statistics^a^** | |
| --- | --- |
|  | What effect did your last ICRC deployment have on your personal development? [My last ICRC deployment had a...] |
| Mann-Whitney U | 298,500 |
| Wilcoxon W | 574,500 |
| Z | -,962 |
| Asymp. Sig. (2-tailed) | ,336 |
| a. Grouping Variable: 1=Doctor; 2=Nurse | |

### Impact of deployment on private situation

| **Descriptive Statistics** | | | | | | | | |
| --- | --- | --- | --- | --- | --- | --- | --- | --- |
|  | N | Mean | Std. Deviation | Minimum | Maximum | Percentiles | | |
|  |  |  |  |  |  | 25th | 50th (Median) | 75th |
| What effect did your last ICRC deployment have on your private situation at home? [My last ICRC deployment had a...] | 53 | 3,43 | 1,047 | 1 | 5 | 3,00 | 3,00 | 4,00 |
| 1=Doctor; 2=Nurse | 57 | 1,44 | ,501 | 1 | 2 | 1,00 | 1,00 | 2,00 |

| **Ranks** | | | | |
| --- | --- | --- | --- | --- |
|  | 1=Doctor; 2=Nurse | N | Mean Rank | Sum of Ranks |
| What effect did your last ICRC deployment have on your private situation at home? [My last ICRC deployment had a...] | Doctor | 28 | 28,75 | 805,00 |
|  | Nurse | 24 | 23,88 | 573,00 |
|  | Total | 52 |  |  |

| **Test Statistics^a^** | |
| --- | --- |
|  | What effect did your last ICRC deployment have on your private situation at home? [My last ICRC deployment had a...] |
| Mann-Whitney U | 273,000 |
| Wilcoxon W | 573,000 |
| Z | -1,239 |
| Asymp. Sig. (2-tailed) | ,216 |
| a. Grouping Variable: 1=Doctor; 2=Nurse | |

## **Grouping variable: profession (surgeon/anesthesiologist/nurse)**

### Weeks of deployment with the ICRC

**null : null**


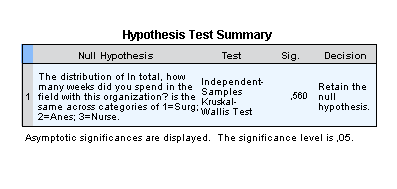


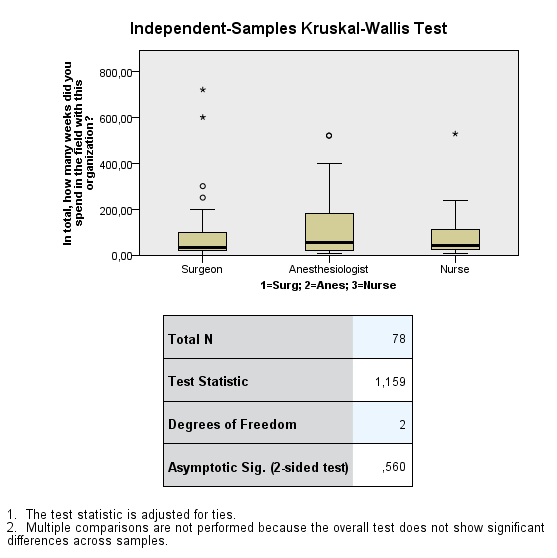


### Years of clinical experience

**null : null**


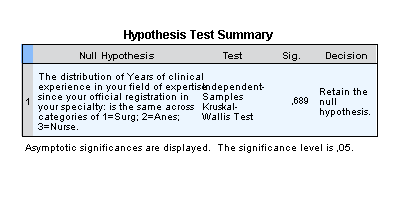


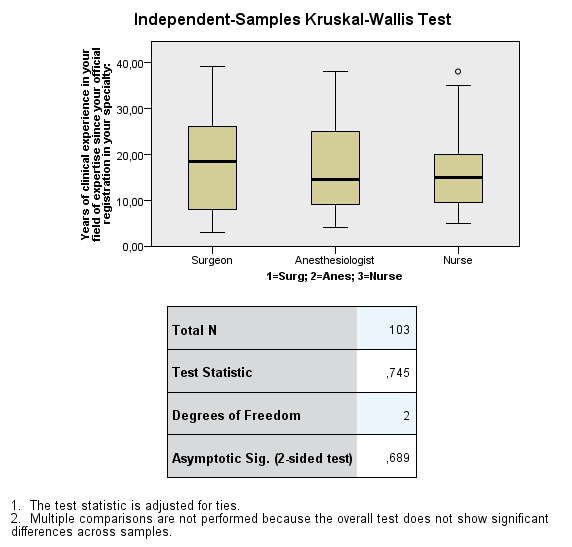


### Overview of pre- and post-deployment rating of self-perceived preparedness to treat paediatric and adult patients

Table 36. Self-perceived preparedness among surgeons, anaesthesiologists and nurses to treat paediatric and adult patients

| **Self-perceived preparedness** | **Surgeons** | **Anesthesiologists** | **Nurses** | **p-value** |
| --- | --- | --- | --- | --- |
| RATED PRE-DEPLOYMENT | | | | |
| Paediatric trauma (median, IQR)  Mean rank  Missing (N, %) | 4.0 (1.0)  43.66  4 (9.5%) | 5.0 (2.0)  48.50  7 (28.0%) | 4.0 (1.0)  33.50  14 (35.0%) | 0.070 |
| Adult trauma (median, IQR)  Mean rank  Missing (N, %) | 5.0 (1.0)  42.86  4 (9.5%) | 5.0 (0.3)  48.94  7 (28.0%) | 4.0 (1.0)  34.37  14 (35.0%) | 0.057 |
| RATED POST-DEPLOYMENT | | | | |
| Paediatric trauma (median, IQR)  Mean rank  Missing (N, %) | 4.0 (1.3)  29.32  1 (2.4%) | 5.0 (1.3)  34.17  1 (4.0%) | 3.0 (2.0)  19.32  3 (7.5%) | 0.053 |
| Adult trauma (median, IQR)  Mean rank  Missing (N, %) | 5.0 (1.0)  30.05  1 (2.4%) | 5.0 (0.0)  35.50  2 (8.0%) | 4.0 (2.0)  20.31  1 (2.5%) | *S-N 0.031*  *A-N 0.049*  S-A 1.000 |

*S Surgeon; A Anaesthesiologist; N Nurse*

### Pre-deployment rating of self-perceived preparedness to treat paediatric patients

**null : null**


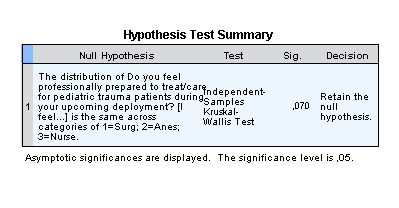


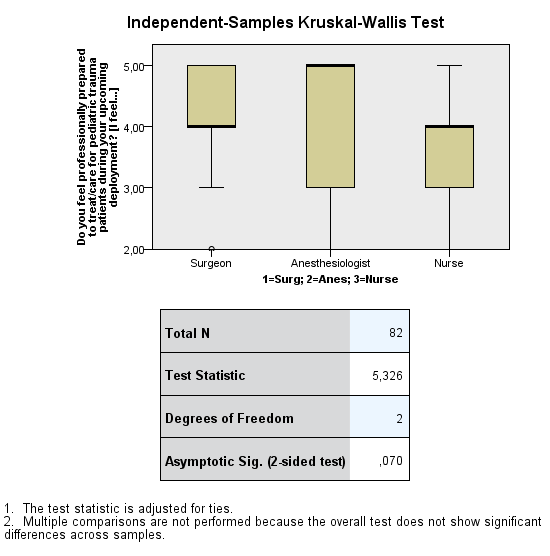


### Pre-deployment rating of self-perceived preparedness to treat adult patients

**null : null**


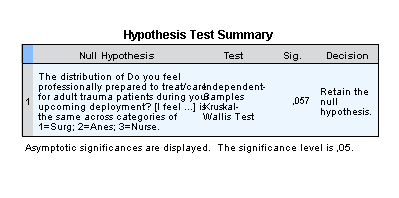


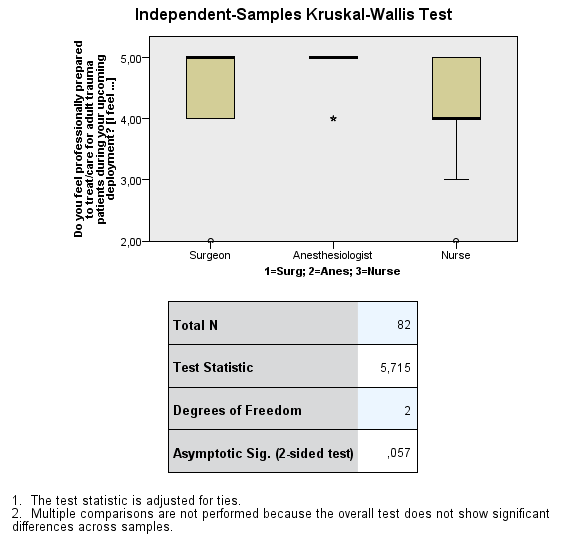


### Number of topics requested for additional training*

**null : null**


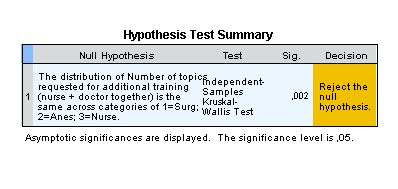


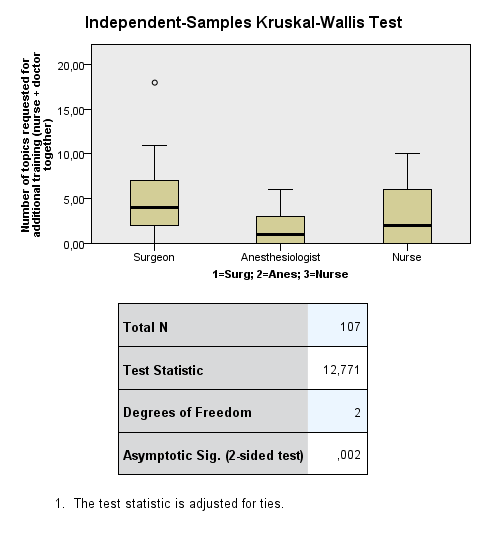


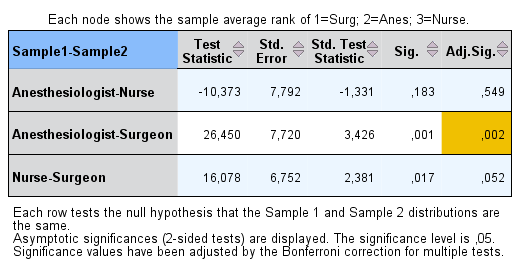


|  | **Surgeons** | **Anesthesiologists** | **Nurses** |
| --- | --- | --- | --- |
| Mean rank | 66.19 | 39.74 | 50.11 |

### Rating of pre-deployment training, knowledge and skills regarding injuries treated during deployment*

**null : null**


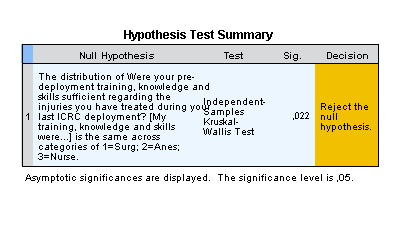


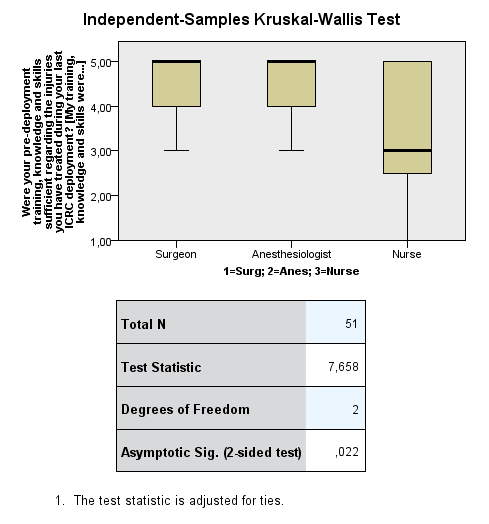


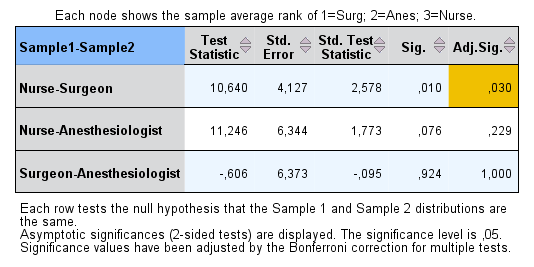


|  | **Surgeons** | **Anesthesiologists** | **Nurses** |
| --- | --- | --- | --- |
| Mean rank | 30.73 | 31.33 | 20.09 |

### Rating of pre-deployment training, knowledge and skills of colleagues

**null : null**


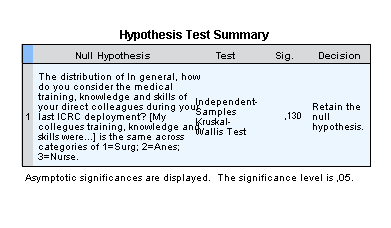


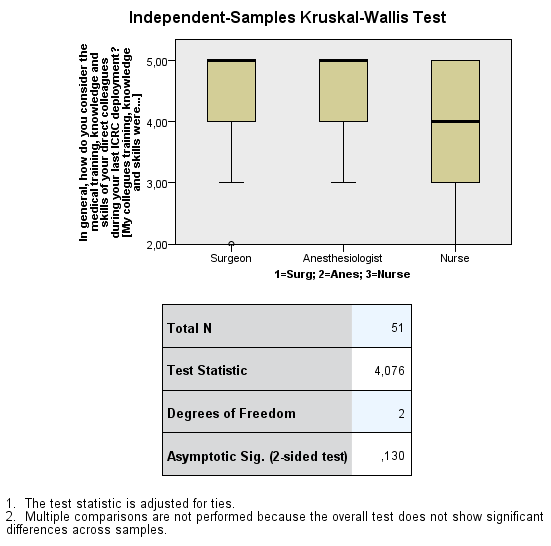


### Rating of equipment to treat adult patients during deployment

**null : null**


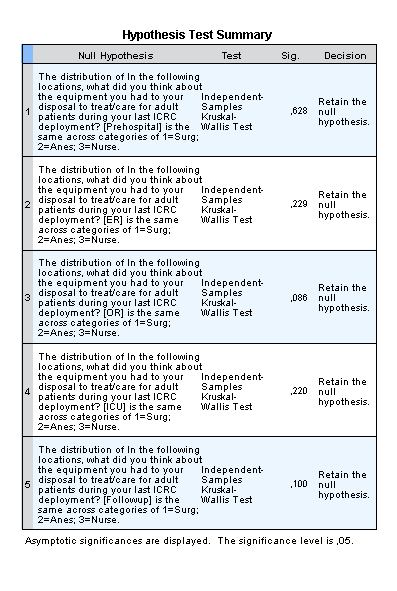


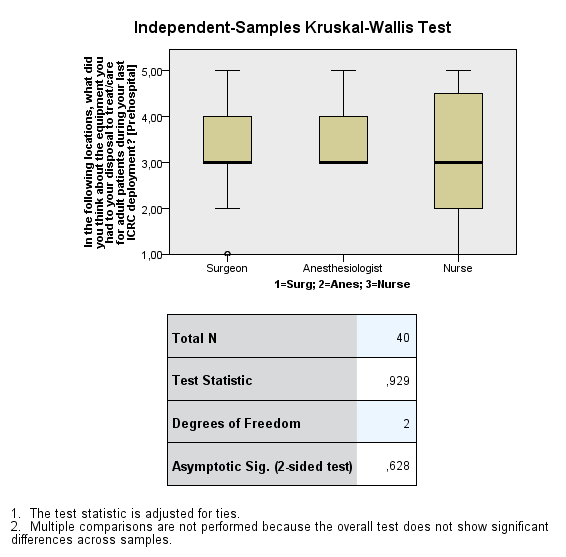


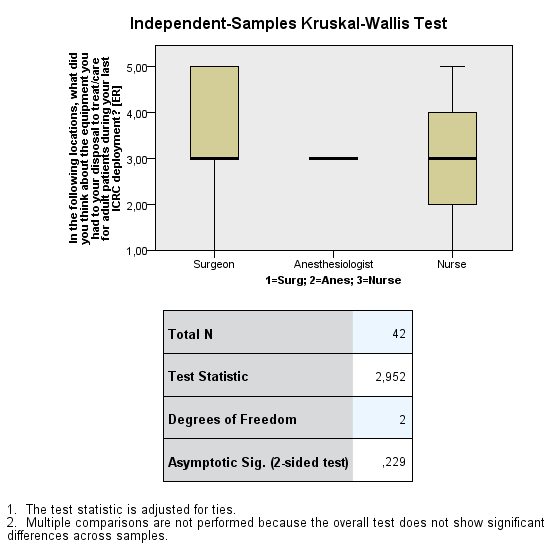


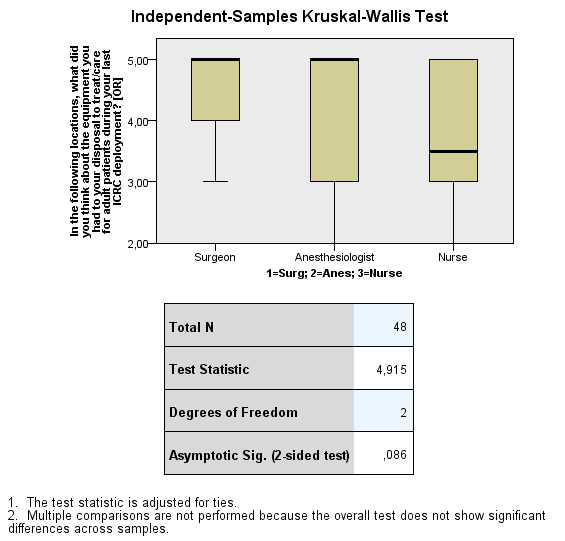


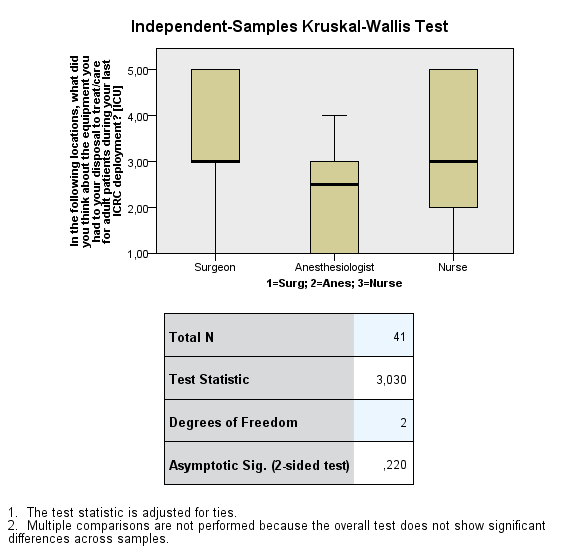


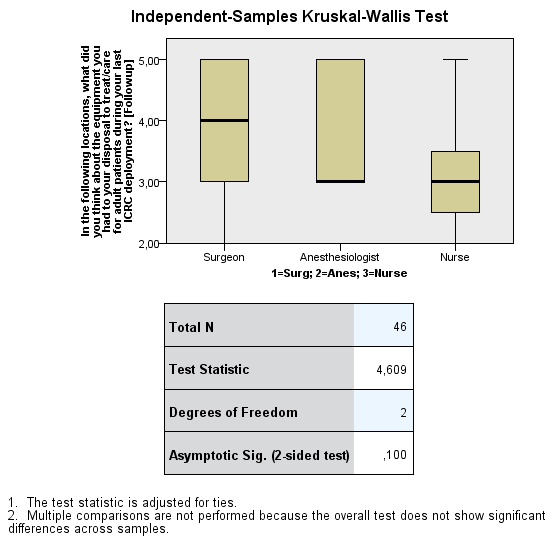


### Rating of equipment to treat paediatric patients during deployment

**null : null**


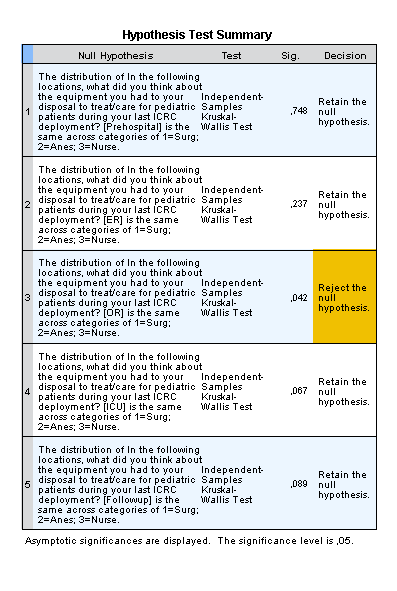


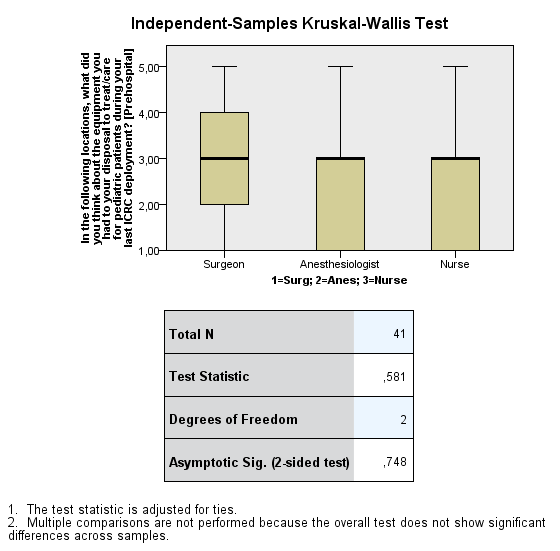


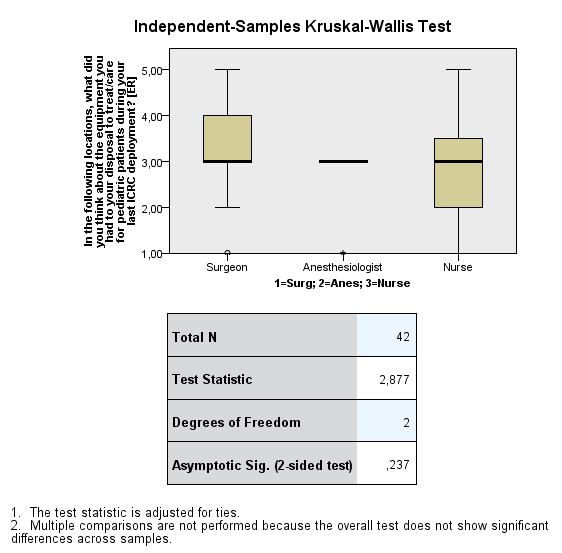


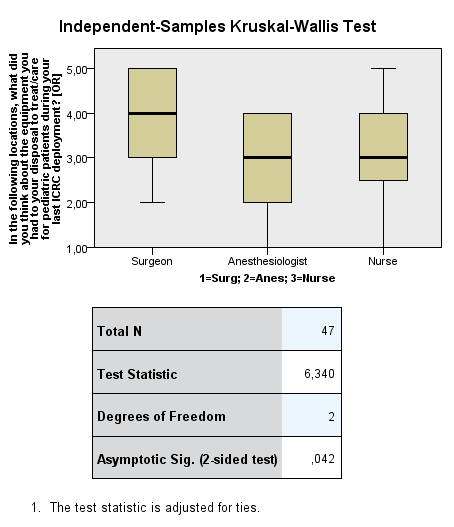


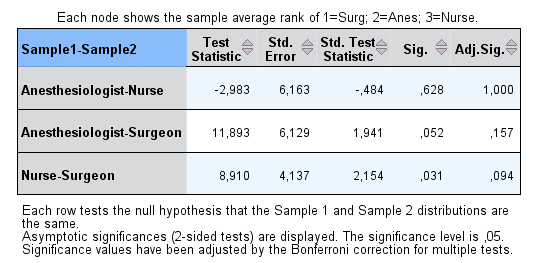


|  | **Surgeons** | **Anesthesiologists** | **Nurses** |
| --- | --- | --- | --- |
| Mean rank | 29.31 | 17.42 | 20.40 |


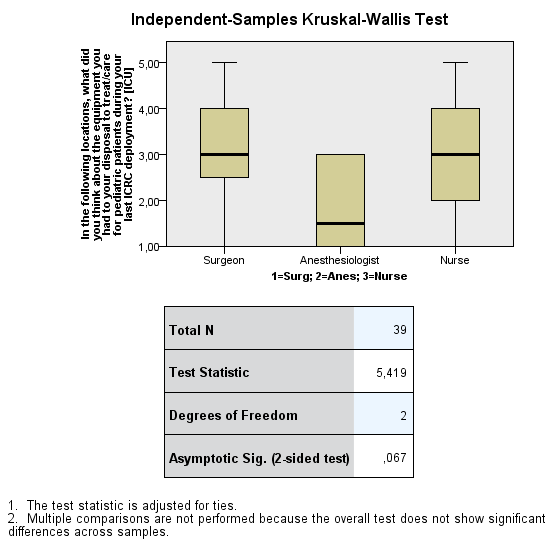


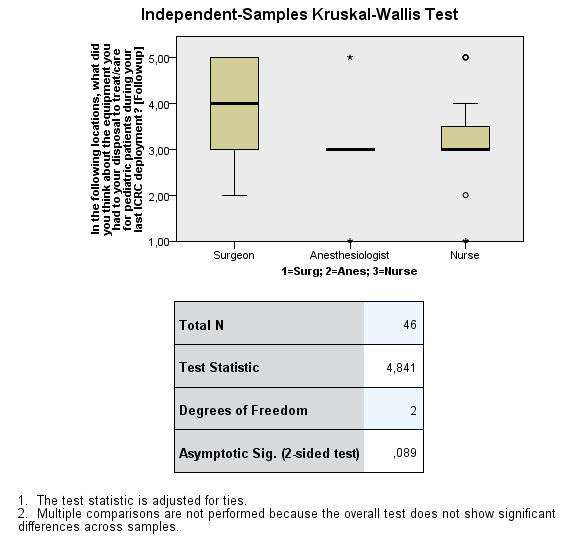


### Post-deployment rating of self-perceived preparedness to treat paediatric and adult patients

**null : null**


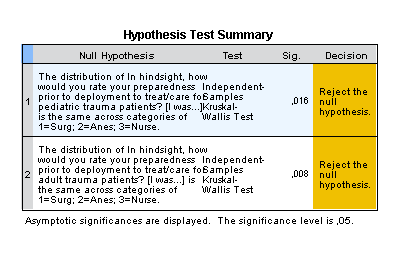


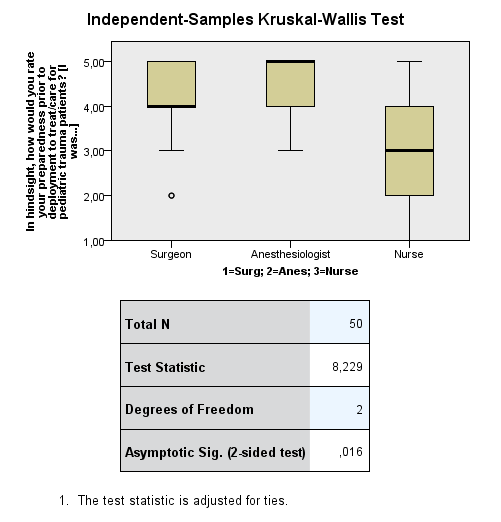


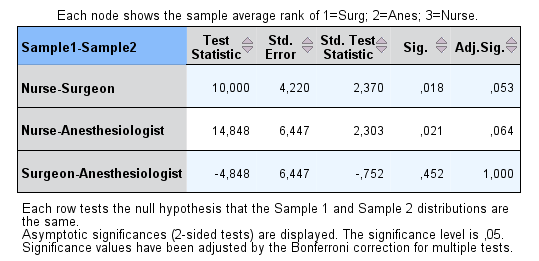


|  | **Surgeons** | **Anesthesiologists** | **Nurses** |
| --- | --- | --- | --- |
| Mean rank | 29.32 | 34.17 | 19.32 |


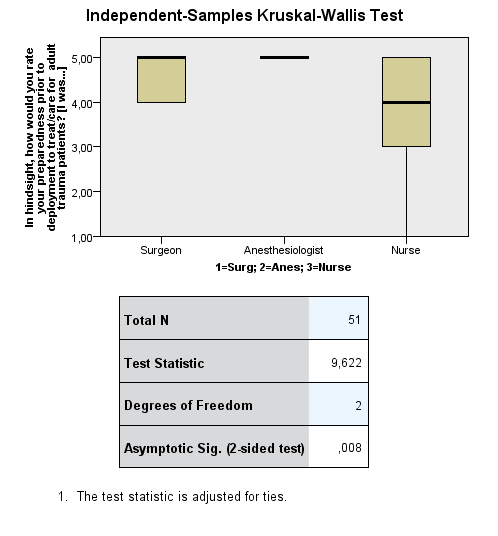


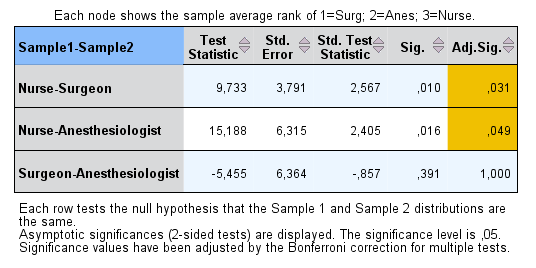


|  | **Surgeons** | **Anesthesiologists** | **Nurses** |
| --- | --- | --- | --- |
| Mean rank | 30.05 | 35.50 | 20.31 |

### Need for peer-to-peer contact, debriefing or professional psychological help during deployment

**null : null**


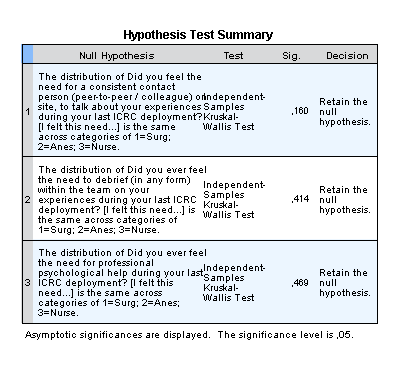


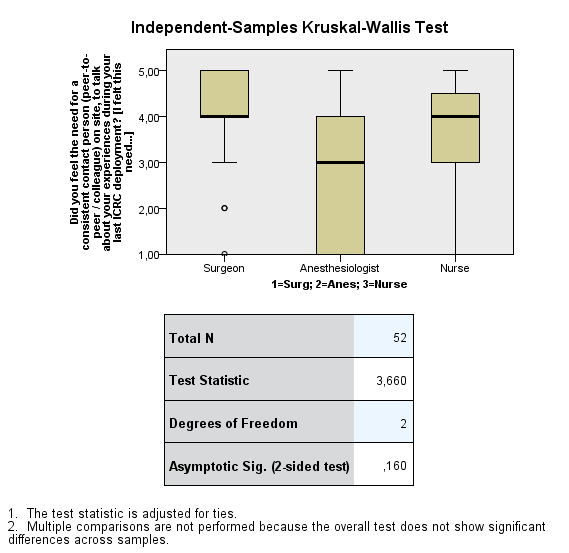


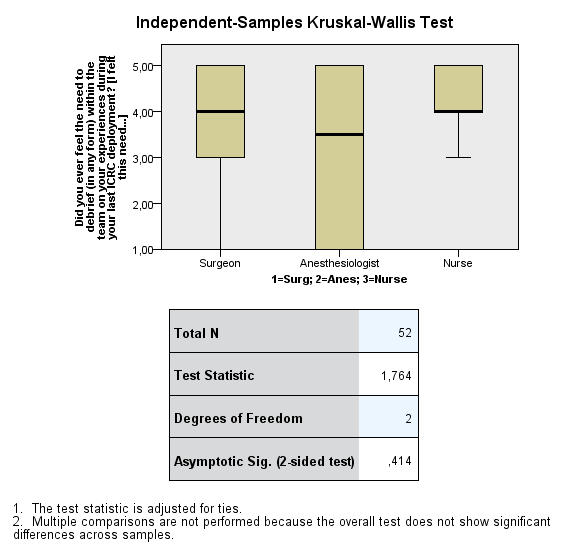


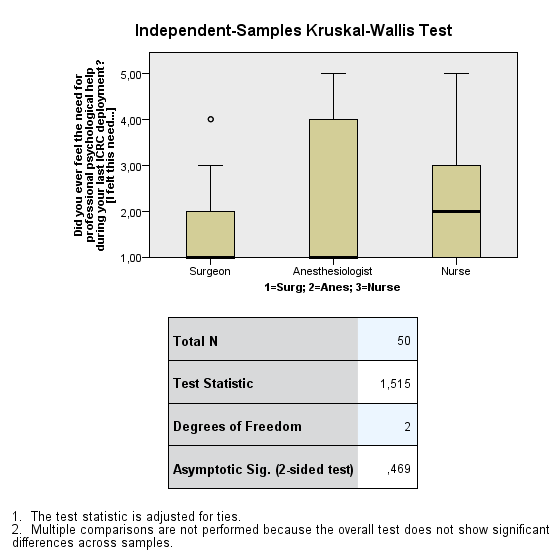


### Impact of deployment on personal development and private situation

**null : null**


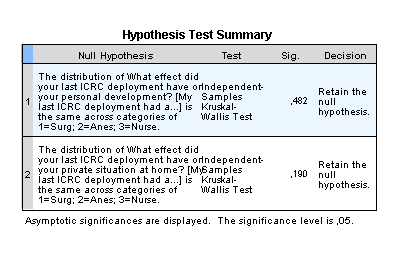


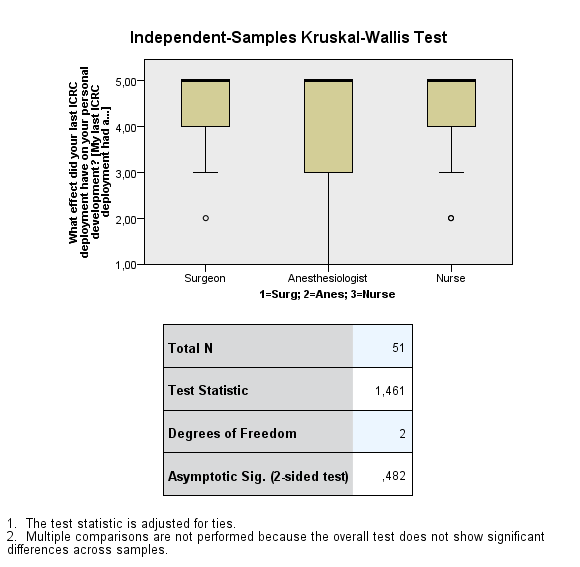


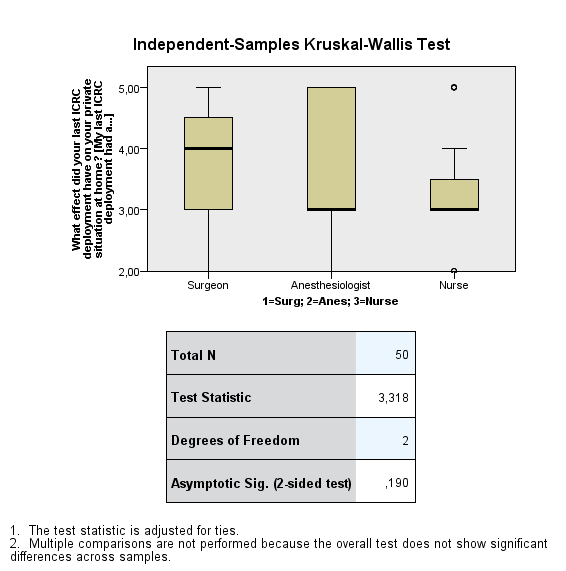


## **Grouping variable: clinical placement (yes/no)**

### Pre-deployment rating of self-perceived preparedness to treat paediatric and adult patients*

| **Descriptive Statistics** | | | | | |
| --- | --- | --- | --- | --- | --- |
|  | N | Mean | Std. Deviation | Minimum | Maximum |
| Do you feel professionally prepared to treat/care for paediatric trauma patients during your upcoming deployment? [I feel...] | 87 | 3,95 | ,914 | 2 | 5 |
| Do you feel professionally prepared to treat/care for adult trauma patients during your upcoming deployment? [I feel ...] | 87 | 4,54 | ,679 | 2 | 5 |
| Did you, as preparation for deployment, participate in a clinical placement in a trauma centre in an area with high rates of severe trauma injuries (expected to be somewhat similar as injuries seen on deployment)? | 88 | ,51 | ,625 | 0 | 2 |

| **Ranks** | | | | |
| --- | --- | --- | --- | --- |
|  | Did you, as preparation for deployment, participate in a clinical placement in a trauma centre in an area with high rates of severe trauma injuries (expected to be somewhat similar as injuries seen on deployment)? | N | Mean Rank | Sum of Ranks |
| Do you feel professionally prepared to treat/care for paediatric trauma patients during your upcoming deployment? [I feel...] | No | 48 | 36,89 | 1770,50 |
|  | Yes | 33 | 46,98 | 1550,50 |
|  | Total | 81 |  |  |
| Do you feel professionally prepared to treat/care for adult trauma patients during your upcoming deployment? [I feel ...] | No | 48 | 37,53 | 1801,50 |
|  | Yes | 33 | 46,05 | 1519,50 |
|  | Total | 81 |  |  |

| **Test Statistics^a^** | | |
| --- | --- | --- |
|  | Do you feel professionally prepared to treat/care for paediatric trauma patients during your upcoming deployment? [I feel...] | Do you feel professionally prepared to treat/care for adult trauma patients during your upcoming deployment? [I feel ...] |
| Mann-Whitney U | 594,500 | 625,500 |
| Wilcoxon W | 1770,500 | 1801,500 |
| Z | -2,001 | -1,864 |
| Asymp. Sig. (2-tailed) | ,045 | ,062 |
| a. Grouping Variable: Did you, as preparation for deployment, participate in a clinical placement in a trauma centre in an area with high rates of severe trauma injuries (expected to be somewhat similar as injuries seen on deployment)? | | |

### Post-deployment rating of self-perceived preparedness to treat paediatric and adult patients

| **Descriptive Statistics** | | | | | |
| --- | --- | --- | --- | --- | --- |
|  | N | Mean | Std. Deviation | Minimum | Maximum |
| In hindsight, how would you rate your preparedness prior to deployment to treat/care for paediatric trauma patients? [I was...] | 53 | 3,81 | 1,161 | 1 | 5 |
| In hindsight, how would you rate your preparedness prior to deployment to treat/care for adult trauma patients? [I was...] | 54 | 4,31 | 1,043 | 1 | 5 |
| Did you, as preparation for deployment, participate in a clinical placement in a trauma centre in an area with high rates of severe trauma injuries (expected to be somewhat similar as injuries seen on deployment)? | 88 | ,51 | ,625 | 0 | 2 |

| **Ranks** | | | | |
| --- | --- | --- | --- | --- |
|  | Did you, as preparation for deployment, participate in a clinical placement in a trauma centre in an area with high rates of severe trauma injuries (expected to be somewhat similar as injuries seen on deployment)? | N | Mean Rank | Sum of Ranks |
| In hindsight, how would you rate your preparedness prior to deployment to treat/care for paediatric trauma patients? [I was...] | No | 21 | 16,07 | 337,50 |
|  | Yes | 13 | 19,81 | 257,50 |
|  | Total | 34 |  |  |
| In hindsight, how would you rate your preparedness prior to deployment to treat/care for adult trauma patients? [I was...] | No | 21 | 15,79 | 331,50 |
|  | Yes | 13 | 20,27 | 263,50 |
|  | Total | 34 |  |  |

| **Test Statistics^a^** | | |
| --- | --- | --- |
|  | In hindsight, how would you rate your preparedness prior to deployment to treat/care for paediatric trauma patients? [I was...] | In hindsight, how would you rate your preparedness prior to deployment to treat/care for adult trauma patients? [I was...] |
| Mann-Whitney U | 106,500 | 100,500 |
| Wilcoxon W | 337,500 | 331,500 |
| Z | -1,118 | -1,590 |
| Asymp. Sig. (2-tailed) | ,264 | ,112 |
| Exact Sig. [2*(1-tailed Sig.)] | ,292^b^ | ,205^b^ |
| a. Grouping Variable: Did you, as preparation for deployment, participate in a clinical placement in a trauma centre in an area with high rates of severe trauma injuries (expected to be somewhat similar as injuries seen on deployment)? | | |
| b. Not corrected for ties. | | |

### Need for peer-to-peer contact, debriefing, and professional psychological help during deployment*

| **Descriptive Statistics** | | | | | |
| --- | --- | --- | --- | --- | --- |
|  | N | Mean | Std. Deviation | Minimum | Maximum |
| Did you feel the need for a consistent contact person (peer-to-peer / colleague) on site, to talk about your experiences during your last ICRC deployment? [I felt this need...] | 54 | 3,56 | 1,313 | 1 | 5 |
| Did you ever feel the need to debrief (in any form) within the team on your experiences during your last ICRC deployment? [I felt this need...] | 55 | 4,04 | 1,122 | 1 | 5 |
| Did you ever feel the need for professional psychological help during your last ICRC deployment? [I felt this need...] | 53 | 1,98 | 1,263 | 1 | 5 |
| Did you go on a clinical placement in a trauma centre as preparation for you most recent mission? | 130 | ,2692 | ,44528 | ,00 | 1,00 |

| **Ranks** | | | | |
| --- | --- | --- | --- | --- |
|  | Did you go on a clinical placement in a trauma centre as preparation for you most recent mission? | N | Mean Rank | Sum of Ranks |
| Did you feel the need for a consistent contact person (peer-to-peer / colleague) on site, to talk about your experiences during your last ICRC deployment? [I felt this need...] | No | 40 | 28,71 | 1148,50 |
|  | Yes | 14 | 24,04 | 336,50 |
|  | Total | 54 |  |  |
| Did you ever feel the need to debrief (in any form) within the team on your experiences during your last ICRC deployment? [I felt this need...] | No | 41 | 28,51 | 1169,00 |
|  | Yes | 14 | 26,50 | 371,00 |
|  | Total | 55 |  |  |
| Did you ever feel the need for professional psychological help during your last ICRC deployment? [I felt this need...] | No | 39 | 29,58 | 1153,50 |
|  | Yes | 14 | 19,82 | 277,50 |
|  | Total | 53 |  |  |

| **Test Statistics^a^** | | | |
| --- | --- | --- | --- |
|  | Did you feel the need for a consistent contact person (peer-to-peer / colleague) on site, to talk about your experiences during your last ICRC deployment? [I felt this need...] | Did you ever feel the need to debrief (in any form) within the team on your experiences during your last ICRC deployment? [I felt this need...] | Did you ever feel the need for professional psychological help during your last ICRC deployment? [I felt this need...] |
| Mann-Whitney U | 231,500 | 266,000 | 172,500 |
| Wilcoxon W | 336,500 | 371,000 | 277,500 |
| Z | -,995 | -,435 | -2,208 |
| Asymp. Sig. (2-tailed) | ,320 | ,664 | ,027 |
| a. Grouping Variable: Did you go on a clinical placement in a trauma centre as preparation for you most recent mission? | | | |

## **Grouping variable: previous deployments (yes/no)**

### Pre-deployment rating of self-perceived preparedness to treat paediatric and adult patients

| **Descriptive Statistics** | | | | | |
| --- | --- | --- | --- | --- | --- |
|  | N | Mean | Std. Deviation | Minimum | Maximum |
| Do you feel professionally prepared to treat/care for paediatric trauma patients during your upcoming deployment? [I feel...] | 87 | 3,95 | ,914 | 2 | 5 |
| Do you feel professionally prepared to treat/care for adult trauma patients during your upcoming deployment? [I feel ...] | 87 | 4,54 | ,679 | 2 | 5 |
| Any deployment with ICRC, MSF, armed forces, or other organization | 107 | ,9533 | ,21205 | ,00 | 1,00 |

| **Ranks** | | | | |
| --- | --- | --- | --- | --- |
|  | Any deployment with ICRC, MSF, armed forces, or other organization | N | Mean Rank | Sum of Ranks |
| Do you feel professionally prepared to treat/care for paediatric trauma patients during your upcoming deployment? [I feel...] | None | 5 | 38,50 | 192,50 |
|  | At least one previous deployment with any organization | 82 | 44,34 | 3635,50 |
|  | Total | 87 |  |  |
| Do you feel professionally prepared to treat/care for adult trauma patients during your upcoming deployment? [I feel ...] | None | 5 | 60,50 | 302,50 |
|  | At least one previous deployment with any organization | 82 | 42,99 | 3525,50 |
|  | Total | 87 |  |  |

| **Test Statistics^a^** | | |
| --- | --- | --- |
|  | Do you feel professionally prepared to treat/care for paediatric trauma patients during your upcoming deployment? [I feel...] | Do you feel professionally prepared to treat/care for adult trauma patients during your upcoming deployment? [I feel ...] |
| Mann-Whitney U | 177,500 | 122,500 |
| Wilcoxon W | 192,500 | 3525,500 |
| Z | -,529 | -1,764 |
| Asymp. Sig. (2-tailed) | ,597 | ,078 |
| a. Grouping Variable: Any deployment with ICRC, MSF, armed forces, or other organization | | |

### Post-deployment rating of self-perceived preparedness to treat paediatric and adult patients

| **Descriptive Statistics** | | | | | |
| --- | --- | --- | --- | --- | --- |
|  | N | Mean | Std. Deviation | Minimum | Maximum |
| In hindsight, how would you rate your preparedness prior to deployment to treat/care for paediatric trauma patients? [I was...] | 53 | 3,81 | 1,161 | 1 | 5 |
| In hindsight, how would you rate your preparedness prior to deployment to treat/care for adult trauma patients? [I was...] | 54 | 4,31 | 1,043 | 1 | 5 |
| Any deployment with ICRC, MSF, armed forces, or other organization | 41 | ,9268 | ,26365 | ,00 | 1,00 |

| **Ranks** | | | | |
| --- | --- | --- | --- | --- |
|  | Any deployment with ICRC, MSF, armed forces, or other organization | N | Mean Rank | Sum of Ranks |
| In hindsight, how would you rate your preparedness prior to deployment to treat/care for paediatric trauma patients? [I was...] | None | 3 | 17,50 | 52,50 |
|  | At least one previous deployment with any organization | 36 | 20,21 | 727,50 |
|  | Total | 39 |  |  |
| In hindsight, how would you rate your preparedness prior to deployment to treat/care for adult trauma patients? [I was...] | None | 3 | 20,83 | 62,50 |
|  | At least one previous deployment with any organization | 37 | 20,47 | 757,50 |
|  | Total | 40 |  |  |

| **Test Statistics^a^** | | |
| --- | --- | --- |
|  | In hindsight, how would you rate your preparedness prior to deployment to treat/care for paediatric trauma patients? [I was...] | In hindsight, how would you rate your preparedness prior to deployment to treat/care for adult trauma patients? [I was...] |
| Mann-Whitney U | 46,500 | 54,500 |
| Wilcoxon W | 52,500 | 757,500 |
| Z | -,417 | -,064 |
| Asymp. Sig. (2-tailed) | ,677 | ,949 |
| Exact Sig. [2*(1-tailed Sig.)] | ,709^b^ | ,962^b^ |
| a. Grouping Variable: Any deployment with ICRC, MSF, armed forces, or other organization | | |
| b. Not corrected for ties. | | |

### Number of topics requested for additional training*

| **Descriptive Statistics** | | | | | |
| --- | --- | --- | --- | --- | --- |
|  | N | Mean | Std. Deviation | Minimum | Maximum |
| Number of topics requested for additional training | 114 | 3,1930 | 3,36004 | ,00 | 18,00 |
| Any deployment with ICRC, MSF, armed forces, or other organization | 107 | ,9533 | ,21205 | ,00 | 1,00 |

| **Ranks** | | | | |
| --- | --- | --- | --- | --- |
|  | Any deployment with ICRC, MSF, armed forces, or other organization | N | Mean Rank | Sum of Ranks |
| Number of topics requested for additional training | None | 5 | 84,40 | 422,00 |
|  | At least one previous deployment with any organization | 102 | 52,51 | 5356,00 |
|  | Total | 107 |  |  |

| **Test Statistics^a^** | |
| --- | --- |
|  | Number of topics requested for additional training |
| Mann-Whitney U | 103,000 |
| Wilcoxon W | 5356,000 |
| Z | -2,271 |
| Asymp. Sig. (2-tailed) | ,023 |
| a. Grouping Variable: Any deployment with ICRC, MSF, armed forces, or other organization | |

## **Grouping variable: onboarding mission (yes/no)**

### Pre-deployment rating of self-perceived preparedness to treat paediatric and adult patients

| **Descriptive Statistics** | | | | | |
| --- | --- | --- | --- | --- | --- |
|  | N | Mean | Std. Deviation | Minimum | Maximum |
| Do you feel professionally prepared to treat/care for paediatric trauma patients during your upcoming deployment? [I feel...] | 87 | 3,95 | ,914 | 2 | 5 |
| Do you feel professionally prepared to treat/care for adult trauma patients during your upcoming deployment? [I feel ...] | 87 | 4,54 | ,679 | 2 | 5 |
| Did you go on an onboarding mission as preparation for your most recent mission? | 114 | ,2632 | ,44229 | ,00 | 1,00 |

| **Ranks** | | | | |
| --- | --- | --- | --- | --- |
|  | Did you go on an onboarding mission as preparation for your most recent mission? | N | Mean Rank | Sum of Ranks |
| Do you feel professionally prepared to treat/care for paediatric trauma patients during your upcoming deployment? [I feel...] | No or missing | 57 | 41,66 | 2374,50 |
|  | Yes | 30 | 48,45 | 1453,50 |
|  | Total | 87 |  |  |
| Do you feel professionally prepared to treat/care for adult trauma patients during your upcoming deployment? [I feel ...] | No or missing | 57 | 43,50 | 2479,50 |
|  | Yes | 30 | 44,95 | 1348,50 |
|  | Total | 87 |  |  |

| **Test Statistics^a^** | | |
| --- | --- | --- |
|  | Do you feel professionally prepared to treat/care for paediatric trauma patients during your upcoming deployment? [I feel...] | Do you feel professionally prepared to treat/care for adult trauma patients during your upcoming deployment? [I feel ...] |
| Mann-Whitney U | 721,500 | 826,500 |
| Wilcoxon W | 2374,500 | 2479,500 |
| Z | -1,257 | -,298 |
| Asymp. Sig. (2-tailed) | ,209 | ,765 |
| a. Grouping Variable: Did you go on an onboarding mission as preparation for your most recent mission? | | |

### Post-deployment rating of self-perceived preparedness to treat paediatric and adult patients

| **Descriptive Statistics** | | | | | |
| --- | --- | --- | --- | --- | --- |
|  | N | Mean | Std. Deviation | Minimum | Maximum |
| In hindsight, how would you rate your preparedness prior to deployment to treat/care for paediatric trauma patients? [I was...] | 53 | 3,81 | 1,161 | 1 | 5 |
| In hindsight, how would you rate your preparedness prior to deployment to treat/care for adult trauma patients? [I was...] | 54 | 4,31 | 1,043 | 1 | 5 |
| Did you go on an onboarding mission as preparation for your most recent mission? | 58 | ,1552 | ,36523 | ,00 | 1,00 |

| **Ranks** | | | | |
| --- | --- | --- | --- | --- |
|  | Did you go on an onboarding mission as preparation for your most recent mission? | N | Mean Rank | Sum of Ranks |
| In hindsight, how would you rate your preparedness prior to deployment to treat/care for paediatric trauma patients? [I was...] | No or missing | 44 | 26,73 | 1176,00 |
|  | Yes | 9 | 28,33 | 255,00 |
|  | Total | 53 |  |  |
| In hindsight, how would you rate your preparedness prior to deployment to treat/care for adult trauma patients? [I was...] | No or missing | 45 | 27,08 | 1218,50 |
|  | Yes | 9 | 29,61 | 266,50 |
|  | Total | 54 |  |  |

| **Test Statistics^a^** | | |
| --- | --- | --- |
|  | In hindsight, how would you rate your preparedness prior to deployment to treat/care for paediatric trauma patients? [I was...] | In hindsight, how would you rate your preparedness prior to deployment to treat/care for adult trauma patients? [I was...] |
| Mann-Whitney U | 186,000 | 183,500 |
| Wilcoxon W | 1176,000 | 1218,500 |
| Z | -,296 | -,511 |
| Asymp. Sig. (2-tailed) | ,767 | ,609 |
| Exact Sig. [2*(1-tailed Sig.)] | ,789^b^ |  |
| a. Grouping Variable: Did you go on an onboarding mission as preparation for your most recent mission? | | |
| b. Not corrected for ties. | | |

### Number of topics requested for additional training*

| **Descriptive Statistics** | | | | | |
| --- | --- | --- | --- | --- | --- |
|  | N | Mean | Std. Deviation | Minimum | Maximum |
| Number of topics requested for additional training | 114 | 3,1930 | 3,36004 | ,00 | 18,00 |
| Did you go on an onboarding mission as preparation for your most recent mission? | 114 | ,2632 | ,44229 | ,00 | 1,00 |

| **Ranks** | | | | |
| --- | --- | --- | --- | --- |
|  | Did you go on an onboarding mission as preparation for your most recent mission? | N | Mean Rank | Sum of Ranks |
| Number of topics requested for additional training | No | 84 | 53,92 | 4529,50 |
|  | Yes | 30 | 67,52 | 2025,50 |
|  | Total | 114 |  |  |

| **Test Statistics^a^** | |
| --- | --- |
|  | Number of topics requested for additional training |
| Mann-Whitney U | 959,500 |
| Wilcoxon W | 4529,500 |
| Z | -1,964 |
| Asymp. Sig. (2-tailed) | ,050 |
| a. Grouping Variable: Did you go on an onboarding mission as preparation for your most recent mission? | |

## **Grouping variable: country of education (low/middle/high income)**

### Pre-deployment rating of self-perceived preparedness to treat paediatric and adult patients

**null : null**


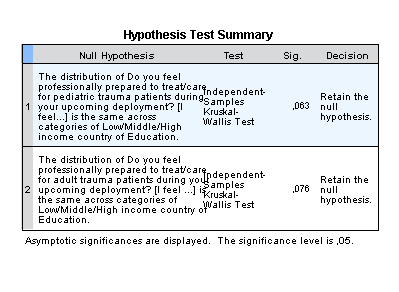


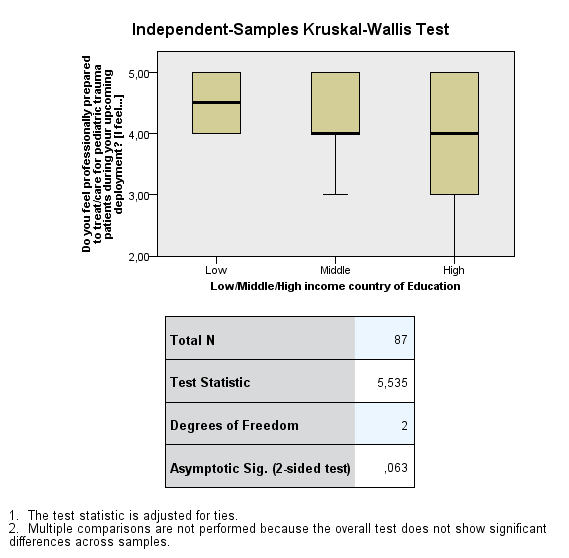


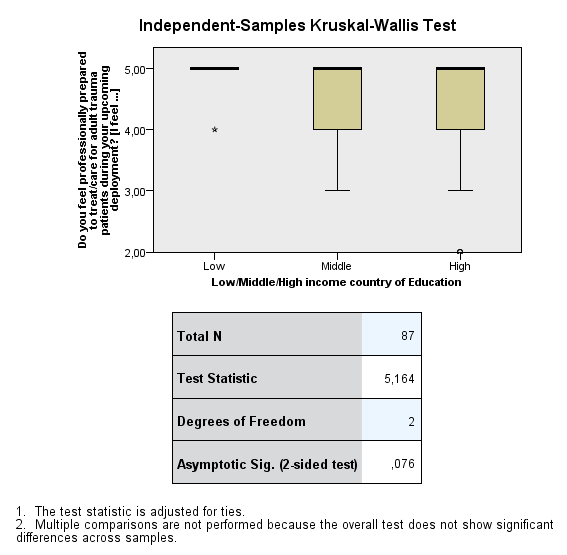


### Post-deployment rating of self-perceived preparedness to treat paediatric and adult patients

**null : null**


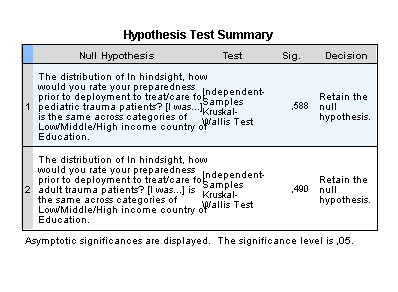


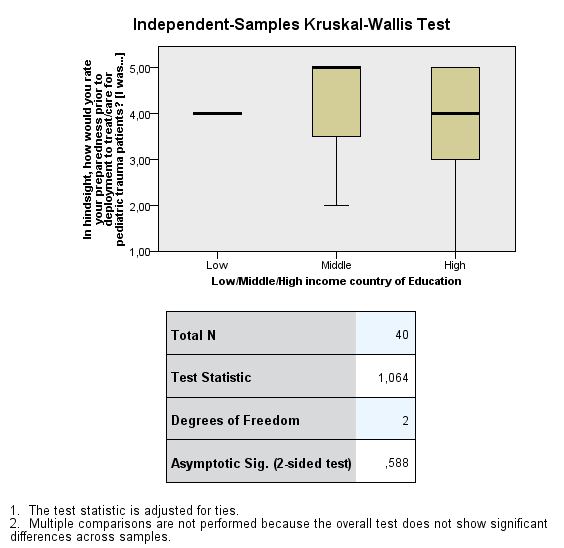


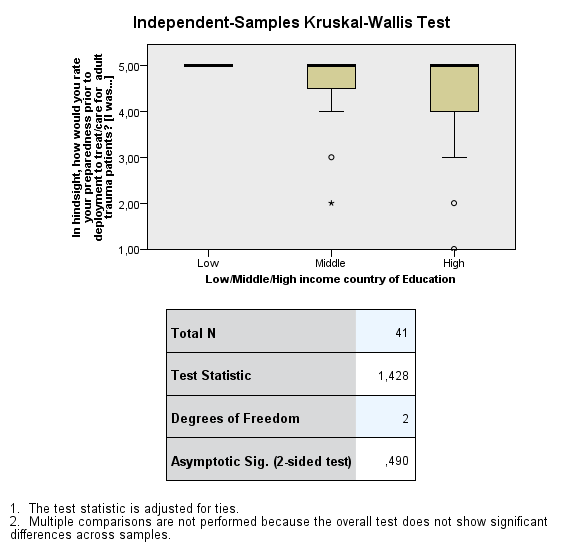


### Rating of equipment to treat adult and paediatric patients during deployment*

**null : null**


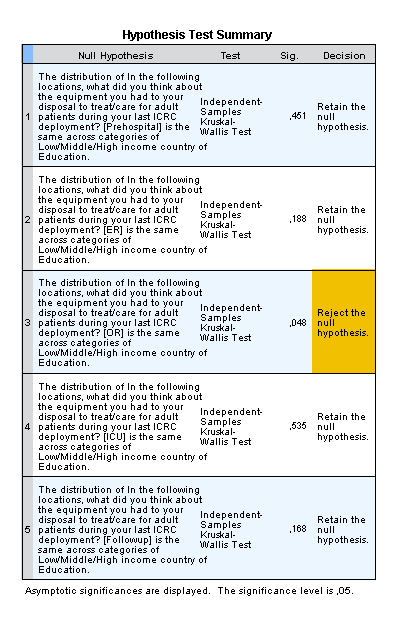


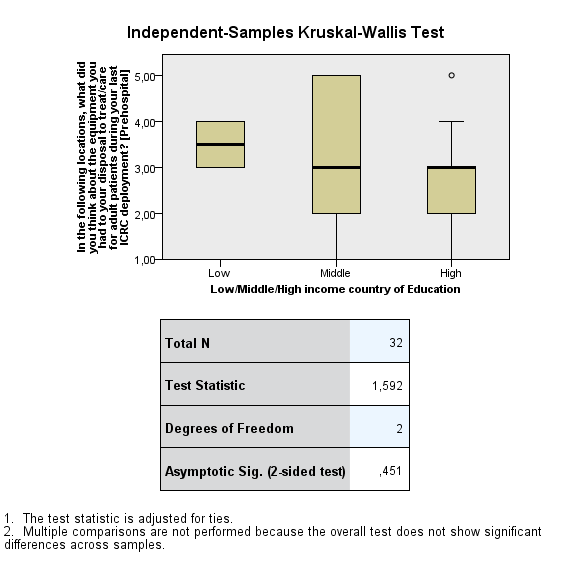


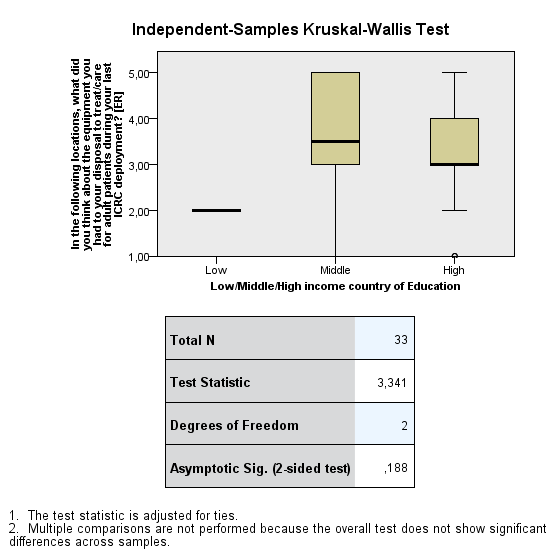


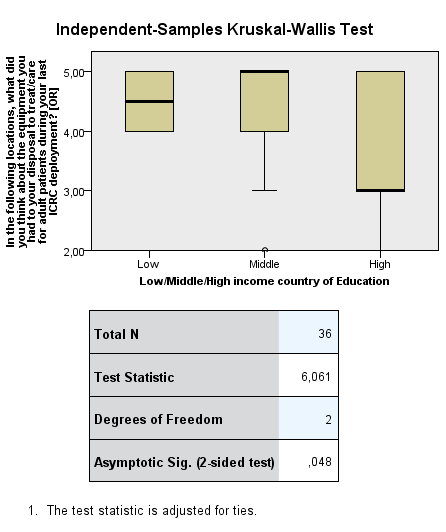

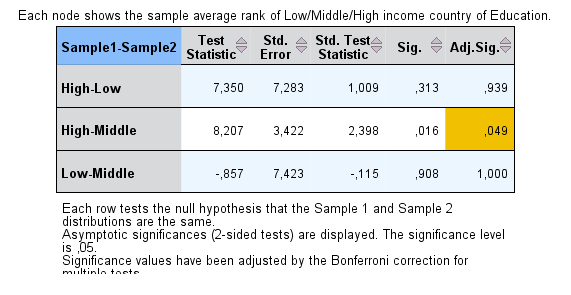


|  | **Low income** | **Middle income** | **High income** |
| --- | --- | --- | --- |
| Mean rank | 22.25 | 23.11 | 14.90 |


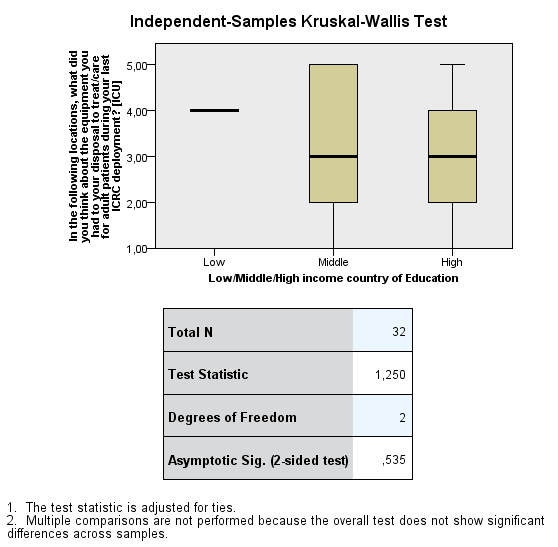


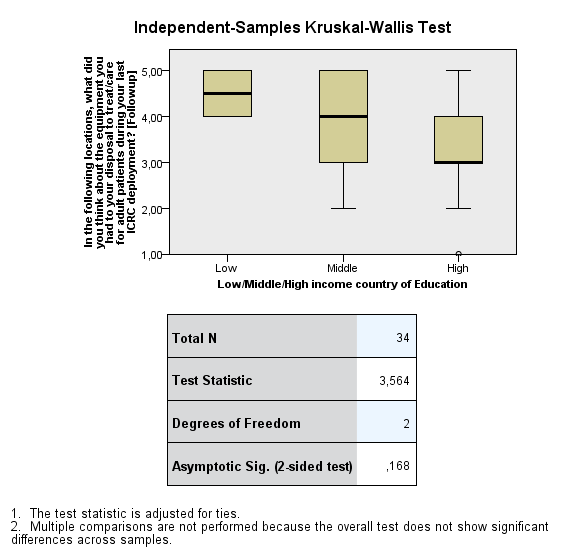


### Rating of equipment to treat paediatric patients during deployment*

**null : null**


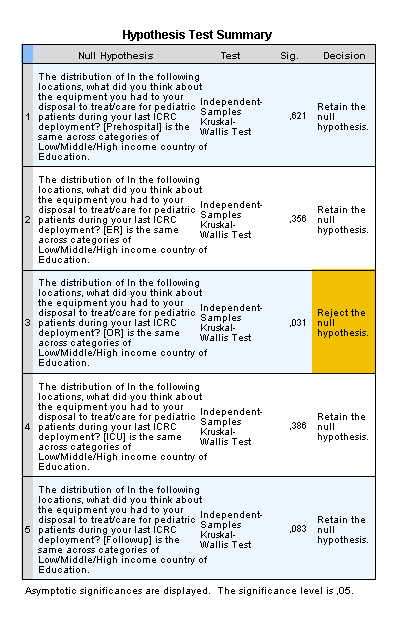


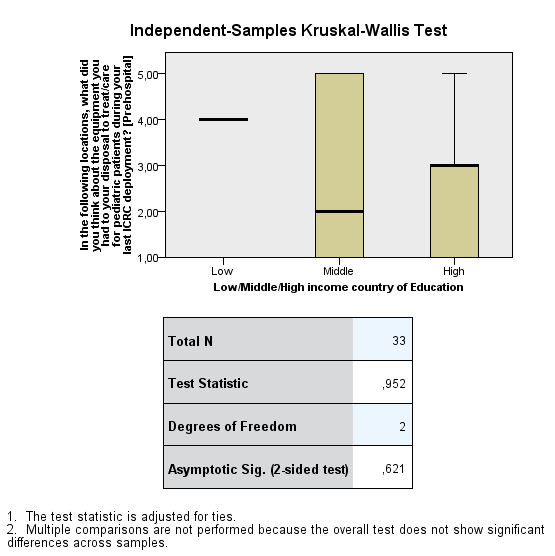

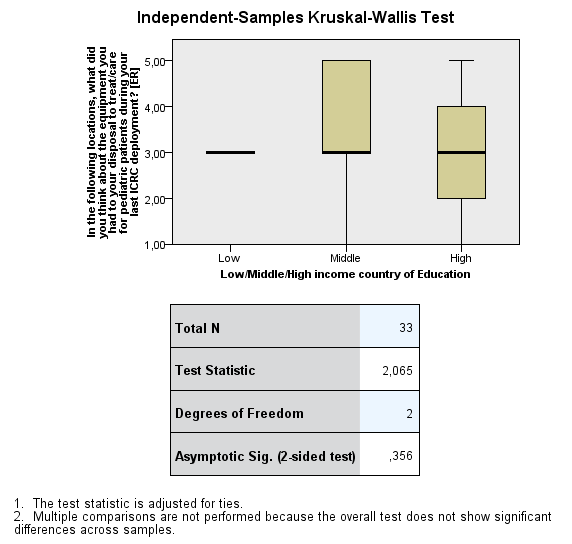


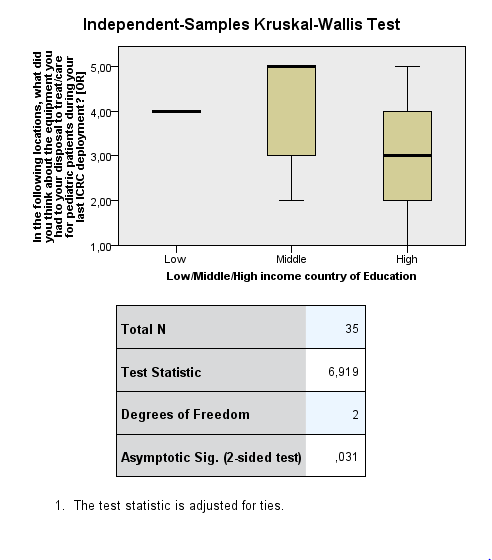


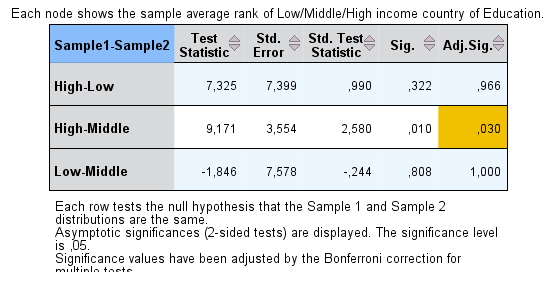


|  | **Low income** | **Middle income** | **High income** |
| --- | --- | --- | --- |
| Mean rank | 21.50 | 23.35 | 14.18 |


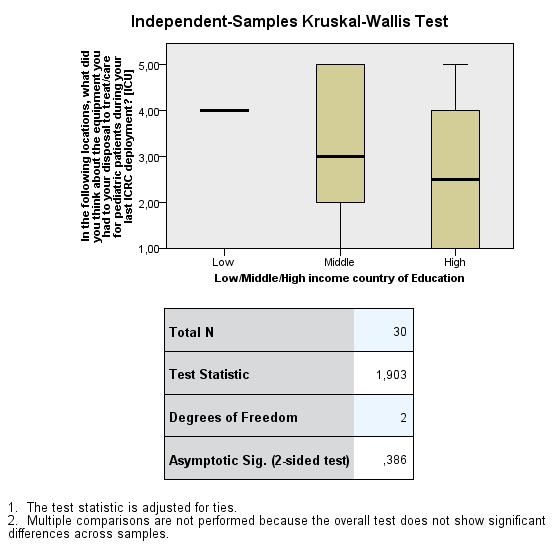


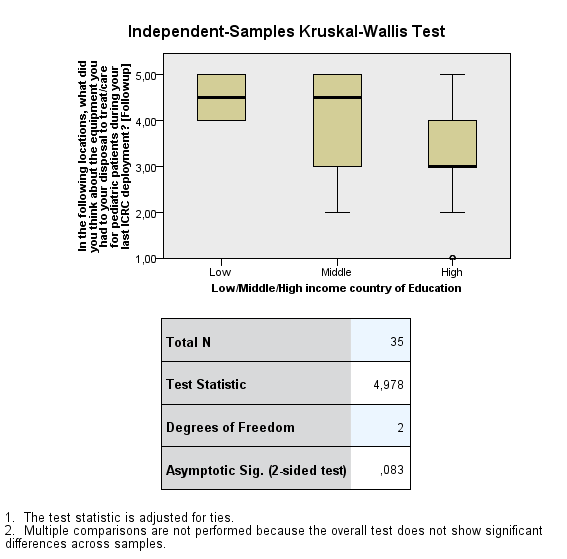


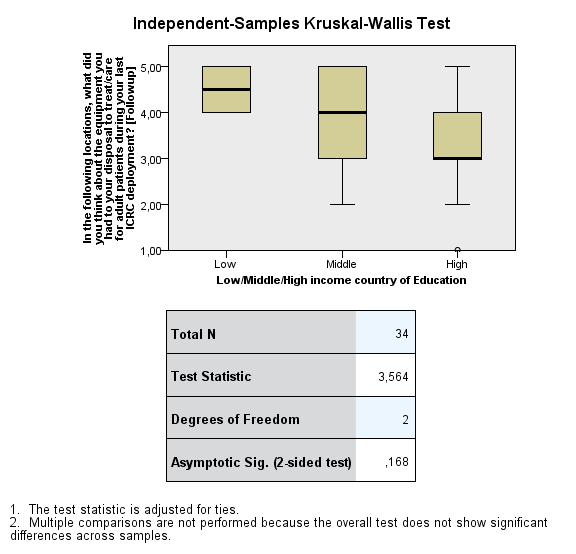


## **Grouping variable: matched or unmatched responses (dropouts)**

### Continuous variables*

| **Descriptive Statistics** | | | | | | | | |
| --- | --- | --- | --- | --- | --- | --- | --- | --- |
|  | N | Mean | Std. Deviation | Minimum | Maximum | Percentiles | | |
|  |  |  |  |  |  | 25th | 50th (Median) | 75th |
| Age (years): | 112 | 47,55 | 10,553 | 27 | 72 | 39,00 | 45,50 | 57,75 |
| Years of clinical experience in your field of expertise since your official registration in your specialty: | 108 | 16,86 | 9,499 | 3 | 39 | 9,00 | 15,00 | 22,75 |
| In total, how many weeks did you spend in the field with ICRC? | 81 | 101,91 | 146,283 | 0 | 720 | 21,00 | 40,00 | 128,00 |
| In total, how many weeks did you spend in the field with Armed Forces? | 20 | 63,80 | 55,113 | 8 | 200 | 13,25 | 55,00 | 115,00 |
| **In total, how many weeks did you spend in the field with MSF?** | **38** | **78,50** | **99,242** | **5** | **500** | **13,75** | **33,50** | **120,00** |
| In total, how many weeks did you spend in the field with other organizations? | 55 | 78,73 | 118,760 | 2 | 540 | 10,00 | 40,00 | 90,00 |
| Period until last onboarding mission with the ICRC | 58 | 2,4138 | 3,08951 | ,00 | 15,00 | ,0000 | 1,0000 | 4,0000 |
| Do you feel professionally prepared to treat/care for paediatric trauma patients during your upcoming deployment? [I feel...] | 87 | 3,95 | ,914 | 2 | 5 | 3,00 | 4,00 | 5,00 |
| Do you feel professionally prepared to treat/care for adult trauma patients during your upcoming deployment? [I feel ...] | 87 | 4,54 | ,679 | 2 | 5 | 4,00 | 5,00 | 5,00 |
| Number of topics requested for additional training | 116 | 3,1379 | 3,35675 | ,00 | 18,00 | ,0000 | 2,5000 | 5,7500 |

| **Ranks** | | | | |
| --- | --- | --- | --- | --- |
|  | Is the post-deployment survey matched to pre-deployment answers? | N | Mean Rank | Sum of Ranks |
| Age (years): | No match | 70 | 57,44 | 4020,50 |
|  | Match | 42 | 54,94 | 2307,50 |
|  | Total | 112 |  |  |
| Years of clinical experience in your field of expertise since your official registration in your specialty: | No match | 67 | 54,08 | 3623,50 |
|  | Match | 41 | 55,18 | 2262,50 |
|  | Total | 108 |  |  |
| In total, how many weeks did you spend in the field with ICRC? | No match | 52 | 41,38 | 2152,00 |
|  | Match | 29 | 40,31 | 1169,00 |
|  | Total | 81 |  |  |
| In total, how many weeks did you spend in the field with Armed Forces? | No match | 12 | 8,88 | 106,50 |
|  | Match | 8 | 12,94 | 103,50 |
|  | Total | 20 |  |  |
| **In total, how many weeks did you spend in the field with MSF?** | **No match** | **23** | **15,74** | **362,00** |
|  | **Match** | **15** | **25,27** | **379,00** |
|  | **Total** | **38** |  |  |
| In total, how many weeks did you spend in the field with other organizations? | No match | 35 | 29,44 | 1030,50 |
|  | Match | 20 | 25,48 | 509,50 |
|  | Total | 55 |  |  |
| Period until last onboarding mission with the ICRC | No match | 32 | 28,80 | 921,50 |
|  | Match | 26 | 30,37 | 789,50 |
|  | Total | 58 |  |  |
| Do you feel professionally prepared to treat/care for paediatric trauma patients during your upcoming deployment? [I feel...] | No match | 51 | 44,61 | 2275,00 |
|  | Match | 36 | 43,14 | 1553,00 |
|  | Total | 87 |  |  |
| Do you feel professionally prepared to treat/care for adult trauma patients during your upcoming deployment? [I feel ...] | No match | 51 | 45,07 | 2298,50 |
|  | Match | 36 | 42,49 | 1529,50 |
|  | Total | 87 |  |  |
| Number of topics requested for additional training | No match | 74 | 55,95 | 4140,50 |
|  | Match | 42 | 62,99 | 2645,50 |
|  | Total | 116 |  |  |

|  | Age (years): | Years of clinical experience in your field of expertise since your official registration in your specialty: | In total, how many weeks did you spend in the field with ICRC? | In total, how many weeks did you spend in the field with Armed Forces? |
| --- | --- | --- | --- | --- |
| Mann-Whitney U | 1404,500 | 1345,500 | 734,000 | 28,500 |
| Wilcoxon W | 2307,500 | 3623,500 | 1169,000 | 106,500 |
| Z | -,394 | -,177 | -,197 | -1,508 |
| Asymp. Sig. (2-tailed) | ,694 | ,859 | ,844 | ,131 |
| Exact Sig. [2*(1-tailed Sig.)] |  |  |  | ,135^b^ |
| a. Grouping Variable: Is the post-deployment survey matched to pre-deployment answers? | | | | |
| b. Not corrected for ties. | | | | |

|  | **In total, how many weeks did you spend in the field with MSF?** | In total, how many weeks did you spend in the field with other organizations? | Period until last onboarding mission with the ICRC |  |
| --- | --- | --- | --- | --- |
| Mann-Whitney U | 86,000 | 299,500 | 393,500 |  |
| Wilcoxon W | 362,000 | 509,500 | 921,500 |  |
| Z | -2,586 | -,884 | -,360 |  |
| **Asymp. Sig. (2-tailed)** | **,010** | ,377 | ,719 |  |
| **Exact Sig. [2*(1-tailed Sig.)]** | **,009^b^** |  |  |  |
| a. Grouping Variable: Is the post-deployment survey matched to pre-deployment answers? | | | | |
| b. Not corrected for ties. | | | | |

|  | Do you feel professionally prepared to treat/care for paediatric trauma patients during your upcoming deployment? [I feel...] | Do you feel professionally prepared to treat/care for adult trauma patients during your upcoming deployment? [I feel ...] | Number of topics requested for additional training |  |
| --- | --- | --- | --- | --- |
| Mann-Whitney U | 887,000 | 863,500 | 1365,500 |  |
| Wilcoxon W | 1553,000 | 1529,500 | 4140,500 |  |
| Z | -,282 | -,551 | -1,101 |  |
| Asymp. Sig. (2-tailed) | ,778 | ,582 | ,271 |  |
| Exact Sig. [2*(1-tailed Sig.)] |  |  |  |  |
| a. Grouping Variable: Is the post-deployment survey matched to pre-deployment answers? | | | | |
| b. Not corrected for ties. | | | | |

### Gender

| **Crosstab** | | | | | |
| --- | --- | --- | --- | --- | --- |
|  | | | Gender | | Total |
|  |  |  | Male | Female |  |
| Is the post-deployment survey matched to pre-deployment answers? | No match | Count | 46 | 24 | 70 |
|  |  | Adjusted Residual | -,6 | ,6 |  |
|  | Match | Count | 30 | 12 | 42 |
|  |  | Adjusted Residual | ,6 | -,6 |  |
| Total | | Count | 76 | 36 | 112 |

| **Chi-Square Tests** | | | | | |
| --- | --- | --- | --- | --- | --- |
|  | Value | df | Asymptotic Significance (2-sided) | Exact Sig. (2-sided) | Exact Sig. (1-sided) |
| Pearson Chi-Square | ,393^a^ | 1 | ,531 |  |  |
| Continuity Correction^b^ | ,175 | 1 | ,676 |  |  |
| Likelihood Ratio | ,397 | 1 | ,529 |  |  |
| Fisher's Exact Test |  |  |  | ,676 | ,340 |
| Linear-by-Linear Association | ,389 | 1 | ,533 |  |  |
| N of Valid Cases | 112 |  |  |  |  |
| a. 0 cells (0,0%) have expected count less than 5. The minimum expected count is 13,50. | | | | | |
| b. Computed only for a 2x2 table | | | | | |

### Country of primary medical education

| **Crosstabulation** | | | | | |
| --- | --- | --- | --- | --- | --- |
|  | | | Is the post-deployment survey matched to pre-deployment answers? | | Total |
|  |  |  | No match | Match |  |
| Low/Middle/High income country of Education | Low | Count | 5 | 3 | 8 |
|  |  | Adjusted Residual | ,0 | ,0 |  |
|  | Middle | Count | 26 | 15 | 41 |
|  |  | Adjusted Residual | ,0 | ,0 |  |
|  | High | Count | 41 | 24 | 65 |
|  |  | Adjusted Residual | ,0 | ,0 |  |
| Total | | Count | 72 | 42 | 114 |

| **Chi-Square Tests** | | | | | | |
| --- | --- | --- | --- | --- | --- | --- |
|  | Value | df | Asymptotic Significance (2-sided) | Exact Sig. (2-sided) | Exact Sig. (1-sided) | Point Probability |
| Pearson Chi-Square | ,003^a^ | 2 | ,999 | 1,000 |  |  |
| Likelihood Ratio | ,003 | 2 | ,999 | 1,000 |  |  |
| Fisher's Exact Test | ,093 |  |  | 1,000 |  |  |
| Linear-by-Linear Association | ,000^b^ | 1 | 1,000 | 1,000 | ,558 | ,123 |
| N of Valid Cases | 114 |  |  |  |  |  |
| a. 1 cells (16,7%) have expected count less than 5. The minimum expected count is 2,95. | | | | | | |
| b. The standardized statistic is ,000. | | | | | | |

| **Crosstabulation** | | | | | |
| --- | --- | --- | --- | --- | --- |
|  | | | Is the post-deployment survey matched to pre-deployment answers? | | Total |
|  |  |  | No match | Match |  |
| Which continent did you get your education | Europe | Count | 29 | 22 | 51 |
|  |  | Adjusted Residual | -1,3 | 1,3 |  |
|  | North America | Count | 6 | 1 | 7 |
|  |  | Adjusted Residual | 1,3 | -1,3 |  |
|  | South America | Count | 6 | 4 | 10 |
|  |  | Adjusted Residual | -,2 | ,2 |  |
|  | Asia | Count | 8 | 7 | 15 |
|  |  | Adjusted Residual | -,8 | ,8 |  |
|  | Africa | Count | 19 | 8 | 27 |
|  |  | Adjusted Residual | ,9 | -,9 |  |
|  | Oceania | Count | 4 | 0 | 4 |
|  |  | Adjusted Residual | 1,6 | -1,6 |  |
| Total | | Count | 72 | 42 | 114 |

| **Chi-Square Tests** | | | | | | |
| --- | --- | --- | --- | --- | --- | --- |
|  | Value | df | Asymptotic Significance (2-sided) | Exact Sig. (2-sided) | Exact Sig. (1-sided) | Point Probability |
| Pearson Chi-Square | 6,001^a^ | 5 | ,306 | ,311 |  |  |
| Likelihood Ratio | 7,567 | 5 | ,182 | ,231 |  |  |
| Fisher's Exact Test | 5,481 |  |  | ,354 |  |  |
| Linear-by-Linear Association | 1,602^b^ | 1 | ,206 | ,214 | ,113 | ,020 |
| N of Valid Cases | 114 |  |  |  |  |  |
| a. 5 cells (41,7%) have expected count less than 5. The minimum expected count is 1,47. | | | | | | |
| b. The standardized statistic is -1,266. | | | | | | |

### Profession

| **Crosstabulation** | | | | | |
| --- | --- | --- | --- | --- | --- |
|  | | | Is the post-deployment survey matched to pre-deployment answers? | | Total |
|  |  |  | No match | Match |  |
| Position in field: | (Registered) nurse | Count | 28 | 15 | 43 |
|  |  | Adjusted Residual | -,4 | ,4 |  |
|  | Anaesthesiologist | Count | 22 | 5 | 27 |
|  |  | Adjusted Residual | 1,8 | -1,8 |  |
|  | Emergency Room physician | Count | 3 | 2 | 5 |
|  |  | Adjusted Residual | -,3 | ,3 |  |
|  | Surgeon | Count | 29 | 18 | 47 |
|  |  | Adjusted Residual | -1,0 | 1,0 |  |
|  | Other | Count | 1 | 0 | 1 |
|  |  | Adjusted Residual | ,7 | -,7 |  |
|  | OT nurse | Count | 3 | 2 | 5 |
|  |  | Adjusted Residual | -,3 | ,3 |  |
| Total | | Count | 86 | 42 | 128 |

| **Chi-Square Tests** | | | | | | |
| --- | --- | --- | --- | --- | --- | --- |
|  | Value | df | Asymptotic Significance (2-sided) | Exact Sig. (2-sided) | Exact Sig. (1-sided) | Point Probability |
| Pearson Chi-Square | 3,950^a^ | 5 | ,557 | ,593 |  |  |
| Likelihood Ratio | 4,497 | 5 | ,480 | ,584 |  |  |
| Fisher's Exact Test | 4,325 |  |  | ,517 |  |  |
| Linear-by-Linear Association | ,420^b^ | 1 | ,517 | ,525 | ,279 | ,041 |
| N of Valid Cases | 128 |  |  |  |  |  |
| a. 6 cells (50,0%) have expected count less than 5. The minimum expected count is33. | | | | | | |
| b. The standardized statistic is ,648. | | | | | | |

### Previous deployments

| **Crosstabulation** | | | | | |
| --- | --- | --- | --- | --- | --- |
|  | | | Is the post-deployment survey matched to pre-deployment answers? | | Total |
|  |  |  | No match | Match |  |
| How many deployments have you done with the ICRC? | 0 | Count | 12 | 12 | 24 |
|  |  | Adjusted Residual | -1,4 | 1,4 |  |
|  | 1 | Count | 9 | 6 | 15 |
|  |  | Adjusted Residual | -,2 | ,2 |  |
|  | 2 | Count | 6 | 8 | 14 |
|  |  | Adjusted Residual | -1,6 | 1,6 |  |
|  | 3 | Count | 10 | 2 | 12 |
|  |  | Adjusted Residual | 1,6 | -1,6 |  |
|  | 4 | Count | 5 | 2 | 7 |
|  |  | Adjusted Residual | ,5 | -,5 |  |
|  | 5 or more | Count | 25 | 11 | 36 |
|  |  | Adjusted Residual | 1,1 | -1,1 |  |
| Total | | Count | 67 | 41 | 108 |

| **Chi-Square Tests** | | | | | | |
| --- | --- | --- | --- | --- | --- | --- |
|  | Value | df | Asymptotic Significance (2-sided) | Exact Sig. (2-sided) | Exact Sig. (1-sided) | Point Probability |
| Pearson Chi-Square | 7,102^a^ | 5 | ,213 | ,216 |  |  |
| Likelihood Ratio | 7,311 | 5 | ,199 | ,229 |  |  |
| Fisher's Exact Test | 6,935 |  |  | ,221 |  |  |
| Linear-by-Linear Association | 3,166^b^ | 1 | ,075 | ,083 | ,042 | ,008 |
| N of Valid Cases | 108 |  |  |  |  |  |
| a. 3 cells (25,0%) have expected count less than 5. The minimum expected count is 2,66. | | | | | | |
| b. The standardized statistic is -1,779. | | | | | | |

| **Crosstabulation** | | | | | |
| --- | --- | --- | --- | --- | --- |
|  | | | Is the post-deployment survey matched to pre-deployment answers? | | Total |
|  |  |  | No match | Match |  |
| How many deployments have you done with the armed forces? | 0 | Count | 55 | 33 | 88 |
|  |  | Adjusted Residual | ,2 | -,2 |  |
|  | 1 | Count | 5 | 2 | 7 |
|  |  | Adjusted Residual | ,5 | -,5 |  |
|  | 2 | Count | 3 | 1 | 4 |
|  |  | Adjusted Residual | ,5 | -,5 |  |
|  | 3 | Count | 1 | 0 | 1 |
|  |  | Adjusted Residual | ,8 | -,8 |  |
|  | 5 or more | Count | 3 | 5 | 8 |
|  |  | Adjusted Residual | -1,5 | 1,5 |  |
| Total | | Count | 67 | 41 | 108 |

| **Chi-Square Tests** | | | | | | |
| --- | --- | --- | --- | --- | --- | --- |
|  | Value | df | Asymptotic Significance (2-sided) | Exact Sig. (2-sided) | Exact Sig. (1-sided) | Point Probability |
| Pearson Chi-Square | 3,213^a^ | 4 | ,523 | ,583 |  |  |
| Likelihood Ratio | 3,504 | 4 | ,477 | ,607 |  |  |
| Fisher's Exact Test | 3,068 |  |  | ,597 |  |  |
| Linear-by-Linear Association | 1,021^b^ | 1 | ,312 | ,354 | ,177 | ,034 |
| N of Valid Cases | 108 |  |  |  |  |  |
| a. 8 cells (80,0%) have expected count less than 5. The minimum expected count is ,38. | | | | | | |
| b. The standardized statistic is 1,010. | | | | | | |

| **Crosstabulation** | | | | | |
| --- | --- | --- | --- | --- | --- |
|  | | | Is the post-deployment survey matched to pre-deployment answers? | | Total |
|  |  |  | No match | Match |  |
| How many deployments have you done with Médecins Sans Frontières (MSF)? | 0 | Count | 42 | 26 | 68 |
|  |  | Adjusted Residual | ,0 | ,0 |  |
|  | 1 | Count | 5 | 2 | 7 |
|  |  | Adjusted Residual | ,5 | -,5 |  |
|  | 2 | Count | 4 | 2 | 6 |
|  |  | Adjusted Residual | ,3 | -,3 |  |
|  | 3 | Count | 5 | 2 | 7 |
|  |  | Adjusted Residual | ,5 | -,5 |  |
|  | 4 | Count | 2 | 0 | 2 |
|  |  | Adjusted Residual | 1,1 | -1,1 |  |
|  | 5 or more | Count | 8 | 9 | 17 |
|  |  | Adjusted Residual | -1,4 | 1,4 |  |
| Total | | Count | 66 | 41 | 107 |

| **Chi-Square Tests** | | | | | | |
| --- | --- | --- | --- | --- | --- | --- |
|  | Value | df | Asymptotic Significance (2-sided) | Exact Sig. (2-sided) | Exact Sig. (1-sided) | Point Probability |
| Pearson Chi-Square | 3,406^a^ | 5 | ,638 | ,674 |  |  |
| Likelihood Ratio | 4,072 | 5 | ,539 | ,645 |  |  |
| Fisher's Exact Test | 2,994 |  |  | ,727 |  |  |
| Linear-by-Linear Association | ,396^b^ | 1 | ,529 | ,534 | ,281 | ,034 |
| N of Valid Cases | 107 |  |  |  |  |  |
| a. 8 cells (66,7%) have expected count less than 5. The minimum expected count is ,77. | | | | | | |
| b. The standardized statistic is ,629. | | | | | | |

| **Crosstabulation** | | | | | |
| --- | --- | --- | --- | --- | --- |
|  | | | Is the post-deployment survey matched to pre-deployment answers? | | Total |
|  |  |  | No match | Match |  |
| How many deployments have you done with other organizations than the ICRC, MSF or the armed forces? | 0 | Count | 29 | 21 | 50 |
|  |  | Adjusted Residual | -,7 | ,7 |  |
|  | 1 | Count | 15 | 8 | 23 |
|  |  | Adjusted Residual | ,4 | -,4 |  |
|  | 2 | Count | 8 | 4 | 12 |
|  |  | Adjusted Residual | ,4 | -,4 |  |
|  | 3 | Count | 0 | 2 | 2 |
|  |  | Adjusted Residual | -1,8 | 1,8 |  |
|  | 4 | Count | 2 | 1 | 3 |
|  |  | Adjusted Residual | ,2 | -,2 |  |
|  | 5 or more | Count | 12 | 5 | 17 |
|  |  | Adjusted Residual | ,8 | -,8 |  |
| Total | | Count | 66 | 41 | 107 |

| **Chi-Square Tests** | | | | | | |
| --- | --- | --- | --- | --- | --- | --- |
|  | Value | df | Asymptotic Significance (2-sided) | Exact Sig. (2-sided) | Exact Sig. (1-sided) | Point Probability |
| Pearson Chi-Square | 4,356^a^ | 5 | ,499 | ,525 |  |  |
| Likelihood Ratio | 4,996 | 5 | ,416 | ,532 |  |  |
| Fisher's Exact Test | 3,949 |  |  | ,568 |  |  |
| Linear-by-Linear Association | ,500^b^ | 1 | ,480 | ,515 | ,260 | ,034 |
| N of Valid Cases | 107 |  |  |  |  |  |
| a. 5 cells (41,7%) have expected count less than 5. The minimum expected count is ,77. | | | | | | |
| b. The standardized statistic is -,707. | | | | | | |

| **Crosstabulation** | | | | | |
| --- | --- | --- | --- | --- | --- |
|  | | | Is the post-deployment survey matched to pre-deployment answers? | | Total |
|  |  |  | No match | Match |  |
| Any deployment with ICRC, MSF, armed forces, or other organization | None | Count | 2 | 3 | 5 |
|  |  | Adjusted Residual | -1,0 | 1,0 |  |
|  | At least one previous deployment with any organization | Count | 64 | 38 | 102 |
|  |  | Adjusted Residual | 1,0 | -1,0 |  |
| Total | | Count | 66 | 41 | 107 |

| **Chi-Square Tests** | | | | | | |
| --- | --- | --- | --- | --- | --- | --- |
|  | Value | df | Asymptotic Significance (2-sided) | Exact Sig. (2-sided) | Exact Sig. (1-sided) | Point Probability |
| Pearson Chi-Square | 1,043^a^ | 1 | ,307 | ,369 | ,285 |  |
| Continuity Correction^b^ | ,303 | 1 | ,582 |  |  |  |
| Likelihood Ratio | 1,007 | 1 | ,316 | ,369 | ,285 |  |
| Fisher's Exact Test |  |  |  | ,369 | ,285 |  |
| Linear-by-Linear Association | 1,034^c^ | 1 | ,309 | ,369 | ,285 | ,215 |
| N of Valid Cases | 107 |  |  |  |  |  |
| a. 2 cells (50,0%) have expected count less than 5. The minimum expected count is 1,92. | | | | | | |
| b. Computed only for a 2x2 table | | | | | | |
| c. The standardized statistic is -1,017. | | | | | | |

### Deployment preparations

| **Crosstabulation** | | | | | |
| --- | --- | --- | --- | --- | --- |
|  | | | Is the post-deployment survey matched to pre-deployment answers? | | Total |
|  |  |  | No match | Match |  |
| Did you, as preparation for deployment, participate in a clinical placement in a trauma centre in an area with high rates of severe trauma injuries (expected to be somewhat similar as injuries seen on deployment)? | No | Count | 28 | 21 | 49 |
|  |  | Adjusted Residual | -,2 | ,2 |  |
|  | Yes | Count | 19 | 14 | 33 |
|  |  | Adjusted Residual | -,1 | ,1 |  |
|  | N/A | Count | 4 | 2 | 6 |
|  |  | Adjusted Residual | ,4 | -,4 |  |
| Total | | Count | 51 | 37 | 88 |

| **Chi-Square Tests** | | | | | | |
| --- | --- | --- | --- | --- | --- | --- |
|  | Value | df | Asymptotic Significance (2-sided) | Exact Sig. (2-sided) | Exact Sig. (1-sided) | Point Probability |
| Pearson Chi-Square | ,202^a^ | 2 | ,904 | ,947 |  |  |
| Likelihood Ratio | ,207 | 2 | ,902 | ,947 |  |  |
| Fisher's Exact Test | ,239 |  |  | 1,000 |  |  |
| Linear-by-Linear Association | ,101^b^ | 1 | ,750 | ,863 | ,444 | ,131 |
| N of Valid Cases | 88 |  |  |  |  |  |
| a. 2 cells (33,3%) have expected count less than 5. The minimum expected count is 2,52. | | | | | | |
| b. The standardized statistic is -,318. | | | | | | |

### Last time worked in regular hospital

| **Crosstabulation** | | | | | |
| --- | --- | --- | --- | --- | --- |
|  | | | Is the post-deployment survey matched to pre-deployment answers? | | Total |
|  |  |  | No match | Match |  |
| When was the last time you have worked in a regular hospital in your home country? | Up until the moment of deployment | Count | 26 | 25 | 51 |
|  |  | Adjusted Residual | -1,7 | 1,7 |  |
|  | Between 1 to 6 months before deployment | Count | 6 | 4 | 10 |
|  |  | Adjusted Residual | ,1 | -,1 |  |
|  | Between 6 months to 1 year before deployment | Count | 4 | 1 | 5 |
|  |  | Adjusted Residual | 1,0 | -1,0 |  |
|  | More than 1 year before deployment | Count | 12 | 6 | 18 |
|  |  | Adjusted Residual | ,8 | -,8 |  |
|  | I have not worked in a regular hospital | Count | 3 | 0 | 3 |
|  |  | Adjusted Residual | 1,5 | -1,5 |  |
| Total | | Count | 51 | 36 | 87 |

| **Chi-Square Tests** | | | | | | |
| --- | --- | --- | --- | --- | --- | --- |
|  | Value | df | Asymptotic Significance (2-sided) | Exact Sig. (2-sided) | Exact Sig. (1-sided) | Point Probability |
| Pearson Chi-Square | 4,775^a^ | 4 | ,311 | ,333 |  |  |
| Likelihood Ratio | 5,948 | 4 | ,203 | ,290 |  |  |
| Fisher's Exact Test | 4,203 |  |  | ,390 |  |  |
| Linear-by-Linear Association | 3,530^b^ | 1 | ,060 | ,062 | ,035 | ,011 |
| N of Valid Cases | 87 |  |  |  |  |  |
| a. 5 cells (50,0%) have expected count less than 5. The minimum expected count is 1,24. | | | | | | |
| b. The standardized statistic is -1,879. | | | | | | |

## **Correlation with: years of clinical experience**

### Pre-deployment rating of self-perceived preparedness to treat paediatric and adult patients

| **Correlations** | | | | | |
| --- | --- | --- | --- | --- | --- |
|  | | |  | Do you feel professionally prepared to treat/care for paediatric trauma patients during your upcoming deployment? [I feel...] | Do you feel professionally prepared to treat/care for adult trauma patients during your upcoming deployment? [I feel ...] |
| Spearman's rho | Years of clinical experience in your field of expertise since your official registration in your specialty: | Correlation Coefficient | 1.000 | .053 | -.144 |
|  |  | Sig. (2-tailed) | . | .625 | .182 |
|  |  | N | 108 | 87 | 87 |
| **. Correlation is significant at the 0.01 level (2-tailed). | | | | | |

### Post-deployment rating of self-perceived preparedness to treat paediatric and adult patients

| **Correlations** | | | | |
| --- | --- | --- | --- | --- |
|  | | | In hindsight, how would you rate your preparedness prior to deployment to treat/care for paediatric trauma patients? [I was...] | In hindsight, how would you rate your preparedness prior to deployment to treat/care for adult trauma patients? [I was...] |
| Spearman's rho | Years of clinical experience in your field of expertise since your official registration in your specialty: | Correlation Coefficient | .271 | .077 |
|  |  | Sig. (2-tailed) | .095 | .637 |
|  |  | N | 39 | 40 |
| **. Correlation is significant at the 0.01 level (2-tailed). | | | | |
